# An aggregation induced enhanced emission-active salicylaldehyde Schiff base: a selective fluorescent sensor for picric acid

**DOI:** 10.1039/d6ra05626a

**Published:** 2026-09-04

**Authors:** Amarsinh R. Mainak, Prabhakar A. Chandanwale, Fatemah M. Al-Dousari, Aabid Hamid, Pranjali S. Kewate, Radhakrishnan M. Tigote, Noorullah Baig, Sheik Saleem Pasha, Ali Shuaib, Sidram R. Pujari

**Affiliations:** a Department of Chemistry, D. B. F. Dayanand College of Arts and Science Solapur Maharashtra 413002 India pujari_aarush@yahoo.co.in armainak12@gmail.com pranjalikewate123@gmail.com; b Department of Chemistry, Sangola Mahavidyalaya Sangola Maharashtra 413002 India chandanwaleprabhakar@gmail.com; c Department of Physics, College of Science, Kuwait University, Sabah Al Salem University City P.O. Box 5969 Safat 13060 Shadadiya Kuwait noor.at.gust@gmail.com fatemah.aldousari@ku.edu.kw; d Department of Chemistry, University of Kashmir Hazratbal – 190006 Jammu & Kashmir India aabidhamid98@gmail.com; e Department of Chemistry, Dr Babasaheb Ambedkar Marathwada University Sub Campus Dharashiv Maharashtra 413501 India rmtigote.chemobad@bamu.ac.in; f CSIR-Central Salt and Marine Chemicals Research Institute Bhavnagar Gujarat 364002 India saleemifplabc@gmail.com; g Biomedical Engineering Unit, Department of Physiology, College of Medicine, Kuwait University P.O. Box 24923 Safat-13110 Kuwait ali.shuaib@ku.edu.kw

## Abstract

In recent luminescent materials research, the development of multifunctional aggregation induced enhanced emission (AIEE)-active molecules through simple and efficient synthetic routes has attracted considerable attention. In this study, a new fluorescence-active organic sensor is synthesized *via* a straightforward Schiff-base condensation between a simple diamine, *N*^1^-tritylethane-1,2-diamine (1), and 5-(*tert*-butyl)-2-hydroxyisophthalaldehyde. The resulting compound, 4-(*tert*-butyl)-2-((*E*)-((2-(tritylamino)ethyl)imino)methyl)-6-((*E*)-((2-(tritylamino)ethyl)imino)methyl)phenol (2), was fully characterized and evaluated as a selective fluorescent probe for the detection of picric acid (PA), a widely used and highly mutagenic nitroaromatic explosive. The sensor exhibits high selectivity and sensitivity toward PA over other aromatic explosives in methanol, addressing critical environmental, health, and security concerns associated with PA contamination and misuse. Quantitative fluorescence quenching studies reveal that the sensing behavior follows the Stern–Volmer model, affording a Stern–Volmer constant *K*_SV_ = 6.309 × 10^4^ M^−1^ and a detection limit of 0.756 µM. Time-resolved fluorescence lifetime measurements strongly support the proposed quenching mechanism, indicating that PA is sensed through a combination of static (ground-state ion-pair/charge-transfer) and dynamic (collisional, PET-driven) quenching, consistent with the upward-curving Stern–Volmer plot and the decrease in fluorescence lifetime upon PA addition. Furthermore, the sensor demonstrates pronounced aggregation induced enhanced emission (AIEE), retaining strong emission in the aggregated state. To demonstrate practical applicability, a portable paper-based sensing platform was successfully fabricated, enabling rapid and visual detection of PA. Overall, this work presents a simple yet effective molecular design strategy for AIEE-active fluorescent sensors with potential applications in explosive detection, environmental monitoring, and portable security screening technologies.

## Abstract 2

A novel PET-based fluorophore was developed for selective sensing of nitroaromatic explosives. It showed pronounced selectivity for picric acid (PA), enabling detection down to 0.756 µM using steady-state and time-resolved fluorescence techniques.

## Graphical abstract

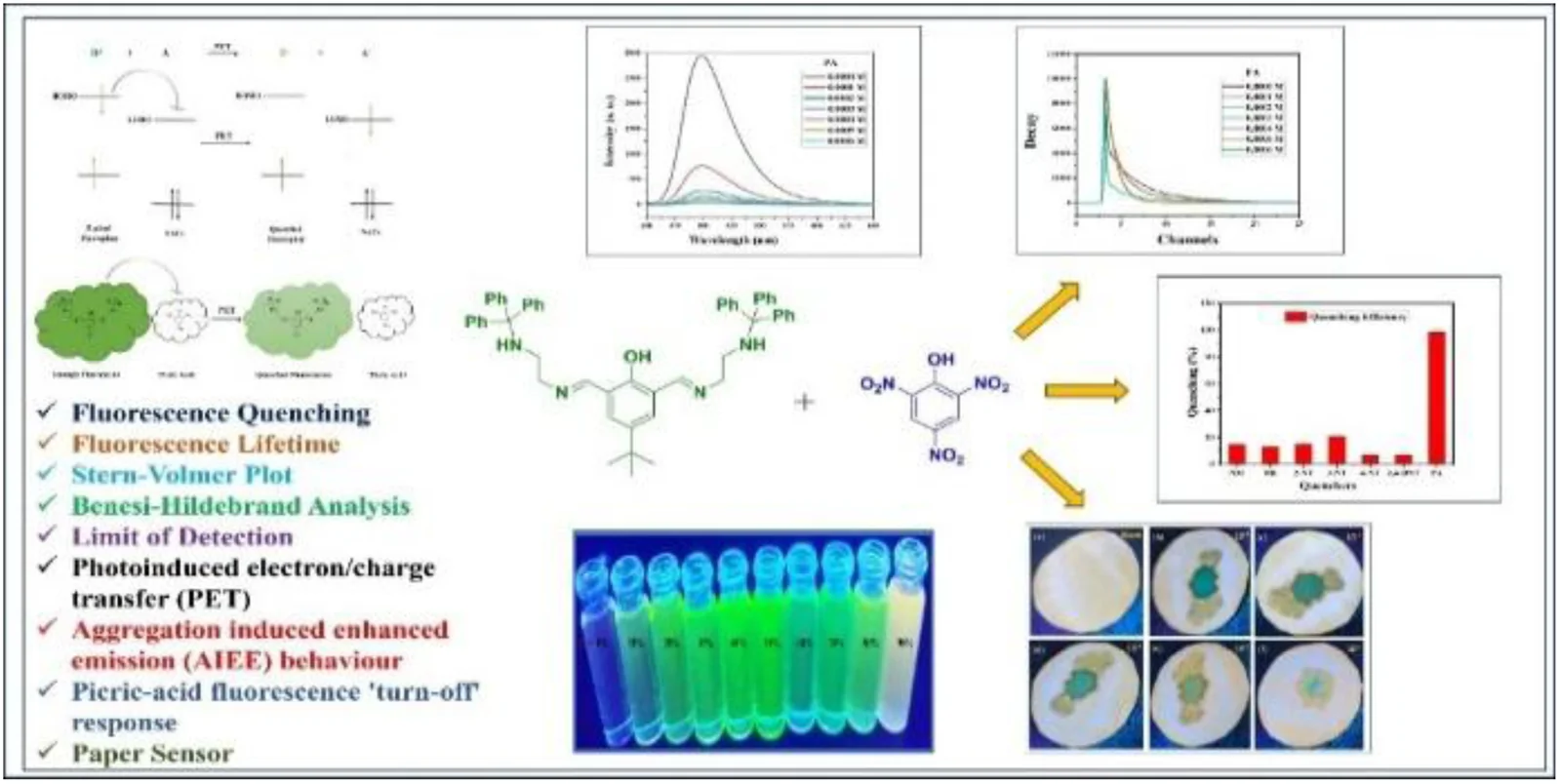

## 1 Introduction

The development of efficient fluorescence-based sensing strategies for nitroaromatic explosives has attracted considerable attention in recent years, particularly for the selective detection of picric acid (PA).^1–7^ Despite significant progress, reports of small-molecule fluorescent chemosensors that simultaneously exhibit high sensitivity and excellent selectivity toward PA-without relying on ion-assisted fluorescence enhancement—remain relatively scarce.^8–10^

The reliable detection of explosive materials continues to pose a major challenge due to the increasing prevalence of sophisticated terrorist activities worldwide. Nitro-substituted aromatic compounds constitute a key class of energetic materials. Among them, 2,4,6-trinitrophenol (TNP), commonly known as picric acid (PA), was extensively used as a high-power explosive prior to World War I owing to its superior detonation performance compared to 2,4,6-trinitrotoluene (TNT) and RDX.^3,11^ Beyond its military applications, PA is widely employed in the manufacture of rocket propellants, dyes, pharmaceuticals, and specialty chemicals.^12,13^ However, its exceptional aqueous solubility renders PA a major environmental contaminant in regions affected by military activity or industrialization. Exposure to PA poses serious health risks, including cyanosis, anemia, carcinogenicity, irritation of the skin and eyes, and damage to the liver, kidneys, and respiratory system. Consequently, the development of analytical techniques capable of detecting trace levels of PA in aqueous media is critically important, particularly at war zones and military installations.^14–16^

A variety of analytical methodologies have been explored for the detection of nitroaromatic explosives, including electrochemical sensing,^17,18^ surface-enhanced Raman spectroscopy,^19^ infrared and Raman spectroscopy,^20^ surface plasmon resonance,^21^ solid-phase microextraction,^22^ and mass spectrometry.^23^ However, the widespread deployment of these techniques is often hindered by inherent limitations such as expensive and sophisticated instrumentation, lengthy analysis times, multi-step sample preparation, and inadequate sensitivity or selectivity. These drawbacks underscore the urgent need for rapid, cost-effective, highly selective, and ultra-sensitive detection platforms for mutagenic explosives such as PA.^24,25^

Fluorescence-based sensing has emerged as a particularly attractive approach owing to its high sensitivity, operational simplicity, and real-time detection capability. Numerous π-electron-rich fluorophores have been designed for nitroaromatic detection, with small organic molecules offering distinct advantages over polymeric systems, including straightforward synthesis, tunable photophysical properties, and multiple fluorescence quenching pathways. In contrast to polymeric sensors-where static quenching dominates due to multiple weak host–guest interactions-small-molecule fluorophores typically operate through dynamic (collisional) quenching mechanisms. These sensing systems can be broadly classified into inorganic and organic categories, with emerging functional organic molecules demonstrating considerable promise. Strategies such as molecular self-assembly, oligo-fluorophore construction, and matrix doping have been employed to enhance analyte binding and promote exciton migration.^26–32^

Electron-rich fluorophores interact efficiently with electron-deficient nitroaromatic explosives through donor–acceptor (D–A) interactions. The strong electron-withdrawing nature of nitro groups lowers the energy of the vacant π orbitals in nitroaromatics, facilitating photoinduced electron transfer (PET), fluorescence resonance energy transfer (FRET), or electron-exchange-mediated energy transfer processes, which result in effective fluorescence quenching.^14,33,34^

Motivated by these considerations, we report herein a new fluorescence sensing platform based on a rationally designed small organic molecule. Building on our previous work, we synthesized a novel Schiff-base-derived fluorophore, 4-(*tert*-butyl)-2-((*E*)-((2-(tritylamino)ethyl)imino)methyl)-6-((*E*)-((2-(tritylamino)ethyl)imino)methyl)phenol (2), *via* a simple and efficient synthetic route. The fluorescence quenching behavior of sensor (2) in methanol (MeOH) was systematically investigated in the presence of various nitroaromatic and non-aromatic analytes, including nitromethane (NM), nitrobenzene (NB), picric acid (PA), 2-nitrotoluene (2-NT), 3-nitrotoluene (3-NT), 4-nitrotoluene (4-NT), and 2,4-dinitrotoluene (2,4-DNT). Key photophysical parameters—such as fluorescence lifetimes (*τ*_0_), quenching rate constants (*k*_q_), Stern–Volmer constants (*K*_SV_), quenching efficiencies, and limits of detection-were determined to elucidate the underlying quenching mechanism. In addition, aggregation induced enhanced emission (AIEE) behavior was examined, and a simple paper-based sensor was developed for the rapid and visual detection of PA, highlighting the practical applicability of the proposed sensing platform.

## 2 Experimental section

### 2.1 Materials

Ethylenediamine, trityl chloride and 5-(*tert*-butyl)-2-hydroxyisophthalaldehyde (TBi), were obtained from Sigma-Aldrich Chemical Company Ltd. UV-grade solvents, including dichloromethane (DCM), hexane, tetrahydrofuran (THF), chloroform, acetone, methanol (MeOH), dimethyl sulfoxide (DMSO), dimethylformamide (DMF), and acetonitrile, along with NaCl, triethylamine, and acetic acid, were purchased from Sigma-Aldrich Chemicals Private Limited. Nitromethane, nitrobenzene, 2-nitrotoluene, 3-nitrotoluene, 4-nitrotoluene, 2,4-dinitrotoluene, and picric acid of analytical reagent (AR) grade were supplied by TCI Chemicals Pvt. Ltd.

### 2.2 Characterization

^1^H NMR (400 MHz) and ^13^C NMR (101 MHz) spectra were recorded on a Bruker AC spectrometer using CDCl_3_ as the solvent and tetramethyl silane (TMS) as an internal standard. Chemical shifts and coupling constants are expressed in *δ* parts per million (ppm) and in hertz (Hz) respectively. The absorption spectra of solutions were recorded at room temperature, on UV-visible spectrophotometer (UV-3600 SHIMADZU) using 1.00 cm quartz cell. The steady-state fluorescence spectra of solutions were recorded on PC based spectro-fluorophotometer (JASCO Model-FP-750, Japan). In all the spectroscopic measurements quartz cuvettes having 1 cm width and 5 cm height are used. Fluorescence lifetime measurements were carried out using an ISS Chronos-BH (ISS-90021) time correlated single photon counting spectrometer (USA). The pulsed light emitting diode with excitation wavelength 448 nm operating at 8 MHz frequency is used as an excitation source. The fluorescence decay curves were recorded setting the emission wavelength at 500 nm and decay curves were fitted biexponentially measure the fluorescence lifetime.

### 2.3 Synthesis

Synthesis of [4-(*tert*-butyl)-2-((*E*)-((2-(tritylamino)ethyl)imino)methyl)-6-((*E*)-((2-(tritylamino)ethyl)imino)methyl)phenol] (2):

A mixture of 5-(*tert*-butyl)-2-hydroxyisophthalaldehyde (TBi) (0.485 mmol, 0.10 g), *N*^1^-tritylethane-1,2-diamine (1) (0.82 mmol, 0.247 g), and a catalytic amount of acetic acid (AcOH) was taken in a round-bottom flask and dissolved in methanol (5 mL). The reaction mixture was stirred under reflux, during which a light-yellow precipitate formed within 30 min. The solid was isolated by filtration and dried under reduced pressure in a vacuum oven for 15 min. Recrystallization of the crude product from methanol afforded the desired compound as a yellow solid in 90% yield. ^1^H NMR (400 MHz, CDCl_3,_ ppm): *δ* 10.58 (s, 1H, OH), 8.67 (s, 2H, HC=N) 7.72 (s, 2H, Ar–H), 7.51–7.46 (m, 12H, Ar–H), 7.32–7.17 (m, 18H, Ar–H), 3.80–3.76 (br-t, 4H, CH_2_), 2.54–2.51 (br-t, 4H, CH_2_), 1.35 (s, 9H, *tert*-butyl–CH_3_).^13^C NMR (101 MHz, CDCl_3,_ ppm): *δ* 146.18, 145.93, 141.20, 128.79, 128.67, 128.10, 127.99, 126.61, 126.41, 70.97, 61.09, 44.40, 34.30, 31.55; FTIR (KBr, cm^−1^): 3406.18 cm^−1^ (–OH), 2920.71 cm^−1^ (=NH), 2851 cm^−1^ (CH_2_–NH–CH_2_), 1635.67 cm^−1^ (C=C), 1121.87 cm^−1^ (C–O bending). ESI-HRMS: calculated for M^+^ C_54_H_54_N_4_O: 775.0532; found 775.0532.

## 3 Results and discussion

### 3.1 Synthesis

**Scheme 1:**
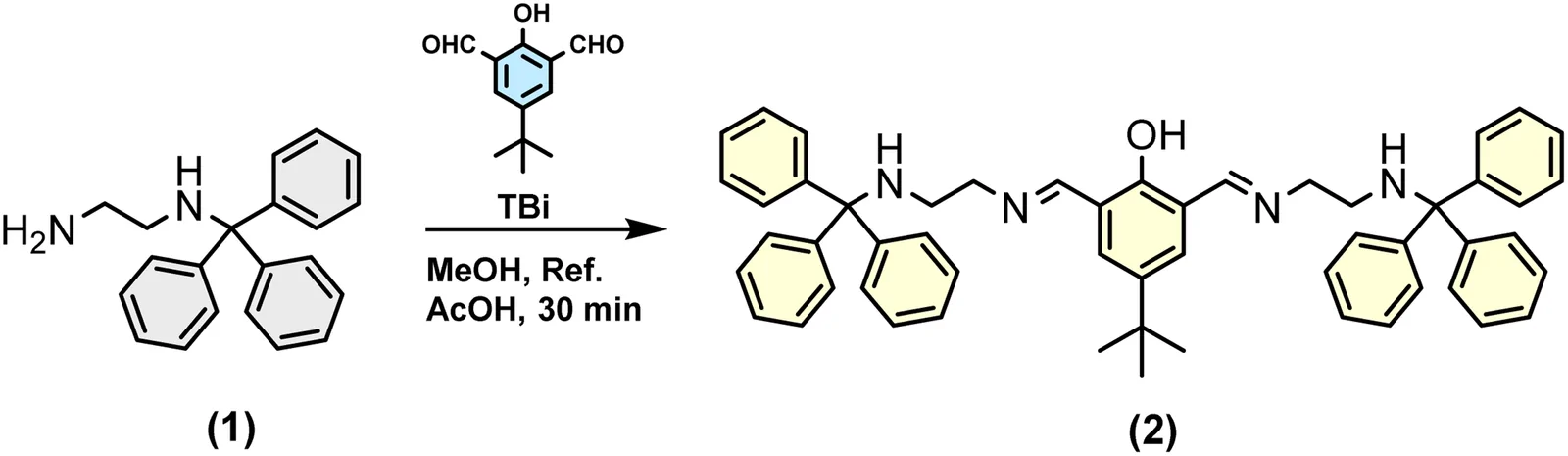
Synthesis of fluorescent sensor (compound-2).

*N* ^1^-Tritylethane-1,2-diamine (1) was synthesized according to a previously reported procedure^35^ (Scheme S1 and Fig. S1–S3 in the SI). The target fluorescent sensor (2) was prepared by attaching compound (1) to the aldehyde groups of commercially available 5-(*tert*-butyl)-2-hydroxyisophthalaldehyde (TBi) through a typical condensation reaction. The reaction was carried out in methanol under reflux conditions using a catalytic amount of acetic acid (AcOH), affording the target compound, 4-(*tert*-butyl)-2-((*E*)-((2-(tritylamino)ethyl)imino)methyl)-6-((*E*)-((2-(tritylamino)ethyl)imino)methyl)phenol (2), within 30 min and in excellent yield (90%) (Scheme 1). The obtained compound (2) exhibited high solubility in common organic solvents, including chloroform, dichloromethane (DCM), and tetrahydrofuran (THF), enabling straightforward structural characterization by ^1^H and ^13^C NMR spectroscopy, high-resolution electrospray ionization mass spectrometry (ESI-HRMS), and FT-IR spectroscopy (see Fig. S4–S7, in the SI file).

**Fig. 1:**
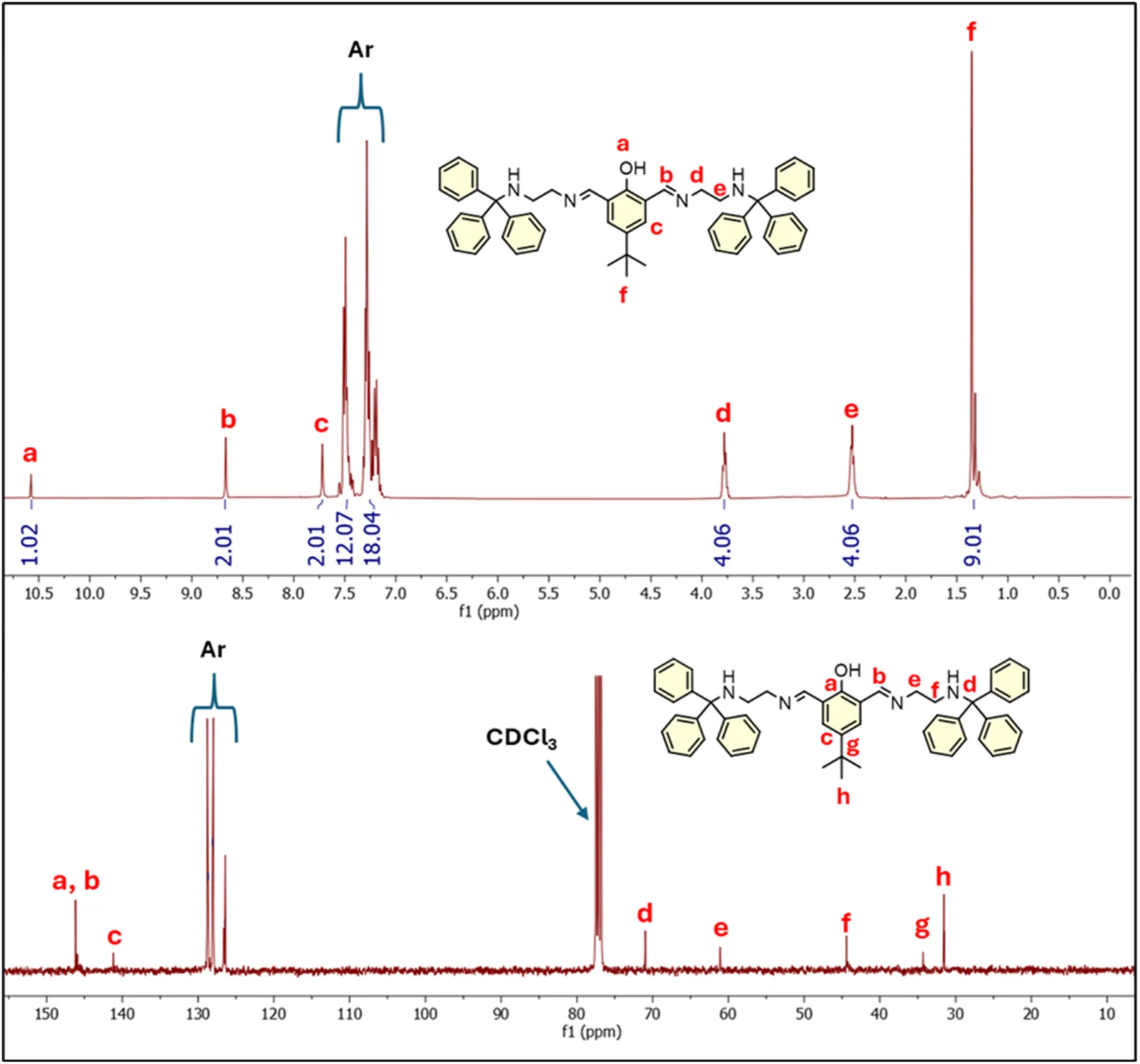
^1^H- (top) and ^13^C- (bottom) NMR of 2 recorded in CDCl_3_.

Fig. 1 portrays the ^1^H-and ^13^C-NMR spectra of the target compound 2, where the spectrum clearly confirmed the presence of all of the desired peaks, namely, the fingerprint chemical shifts of the hydroxyl (–OH), and imine (HC=N), group at 10.58 ppm, and 8.67 ppm, respectively (*c.f.* peaks labeled a, b in Fig. 1). The chemical shifts in the ^1^H-NMR spectrum of 2 ranging from 7.72 ppm to 7.17 ppm are assigned to the aromatic protons (*c.f.* peaks labeled c, Ar in Fig. 1), while those detected at 3.78 ppm and 2.53 ppm are attributed to the characteristic methylene (–CH_2_–) protons of the diamine groups (*c.f.* peaks labeled d, e in Fig. 1). Likewise, the chemical shifts observed at 1.35 ppm are assigned to the methyl (–CH_3_) protons of the tertiary butyl group (*c.f.* peaks labeled f in Fig. 1). ^13^C-NMR spectrum of 2 displays all the expected aromatic peaks in the range of 128.7–126.4 ppm in addition to the chemical shifts of all the aliphatic carbons such as trityl, diamine and tertiary butyl groups at 70.9 ppm, 61.0 ppm, 44.4 ppm, 34.3 ppm, and 31.5 ppm (Fig. 1 and S5 in the SI file). In addition, the high purity of 2 was confirmed ESI-HRMS (Fig. S6 in the SI file).

### 3.2 Photophysical properties

**Fig. 2:**
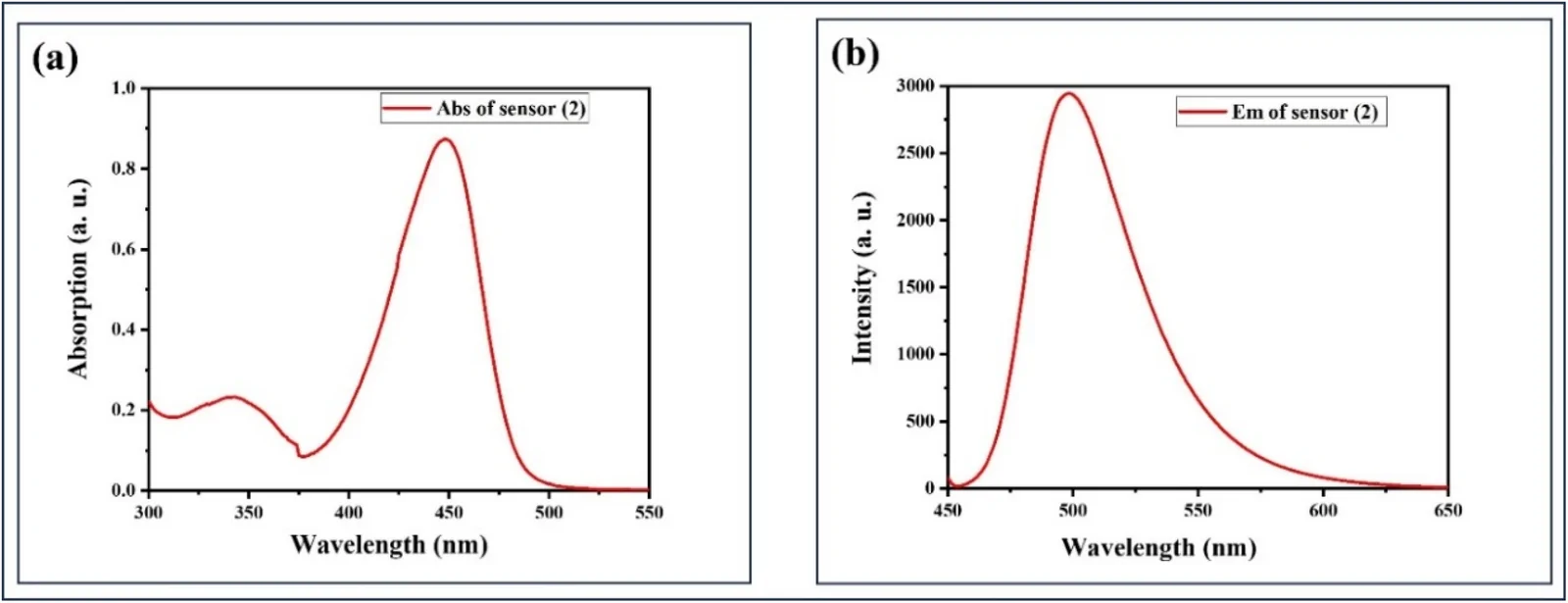
Absorption (a) and emission spectrum (b) of sensor (2).

The photophysical properties of sensor 2 were examined using UV-visible absorption and fluorescence emission spectroscopy. In methanol, the absorption spectrum displays a broad and intense band centered at 448 nm, along with a weaker band at 348 nm (Fig. 2a). The high-energy absorption band is attributed to localized π–π* transitions within the aromatic framework, whereas the lower-energy, intense band is ascribed to an intramolecular charge transfer (ICT) transition originating from the electron-donating tritylamine moieties to the electron-accepting imine group.^36,37^

The fluorescence emission spectrum of sensor 2 (1 × 10^−4^ M), recorded in methanol at 298 K, exhibits a broad emission band with a maximum at 500 nm (Fig. 2b). This red-shifted emission relative to the absorption maximum is consistent with relaxation from an ICT-excited state, indicating significant electronic delocalization in the excited state. The broad emission profile further suggests conformational flexibility of the molecular framework in solution, which is characteristic of Schiff-base-derived fluorophores.^36,38^

### 3.3 Detection of nitroaromatic explosives

**Fig. 3:**
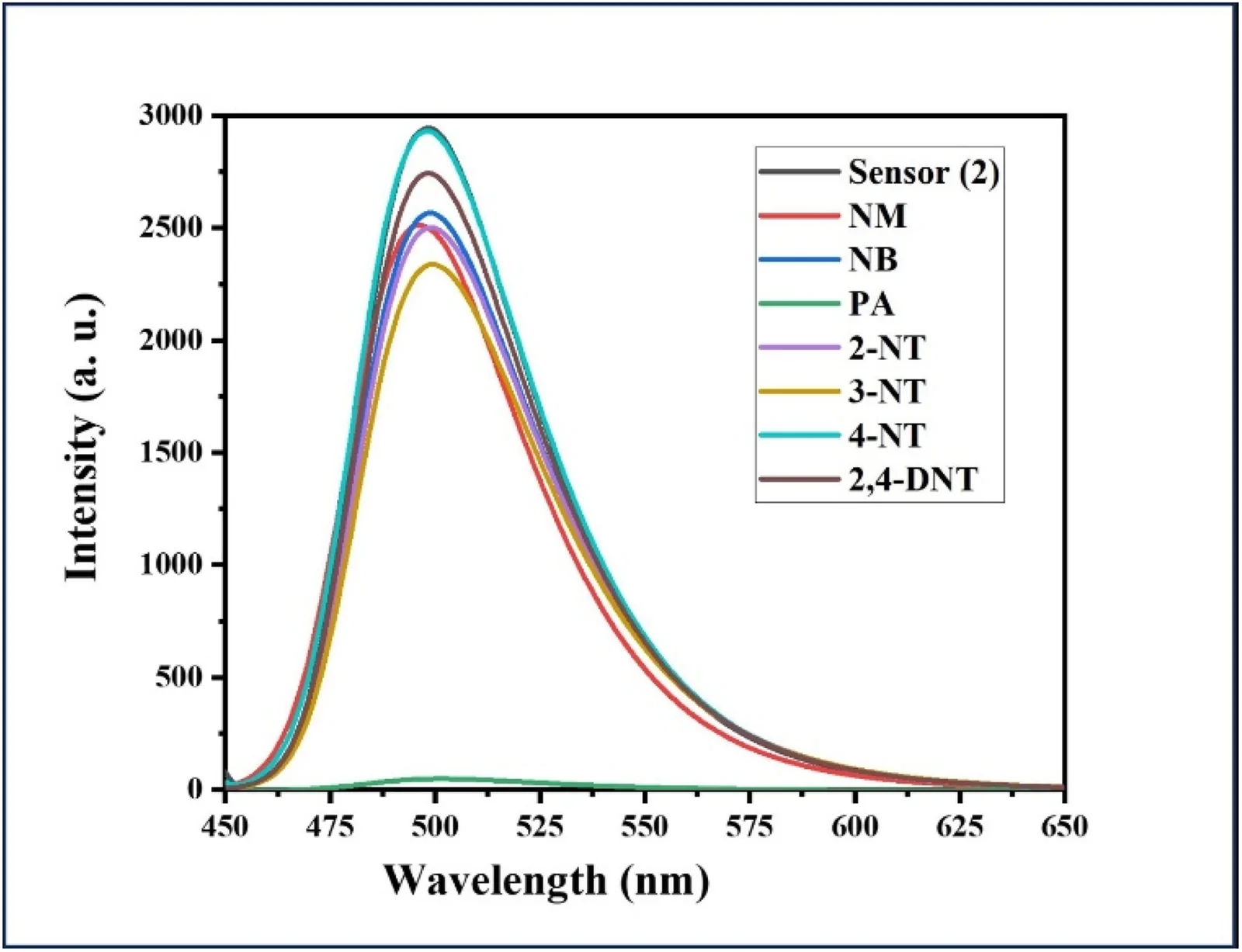
Fluorescence spectra of sensor (2) with *c* = 1 × 10^−4^ M in MeOH upon the addition of 5 × 10^−4^ M of different aromatic/non-aromatic compounds (*λ*_ex_ = 448 nm).

The fluorescence response of sensor 2 was evaluated in methanol at 298 K in the presence and absence of various aromatic and non-aromatic analytes. Upon the addition of nitroaromatic and aliphatic interferents (5 × 10^−4^ M), including nitromethane (NM), nitrobenzene (NB), 2-nitrotoluene (2-NT), 3-nitrotoluene (3-NT), 4-nitrotoluene (4-NT), and 2,4-dinitrotoluene (2,4-DNT), no appreciable change in the fluorescence intensity of sensor 2 was observed. In sharp contrast, the introduction of picric acid (PA, 5 × 10^−4^ M) resulted in a pronounced decrease in emission intensity, indicative of highly efficient fluorescence quenching (Fig. 3).

**Fig. 4:**
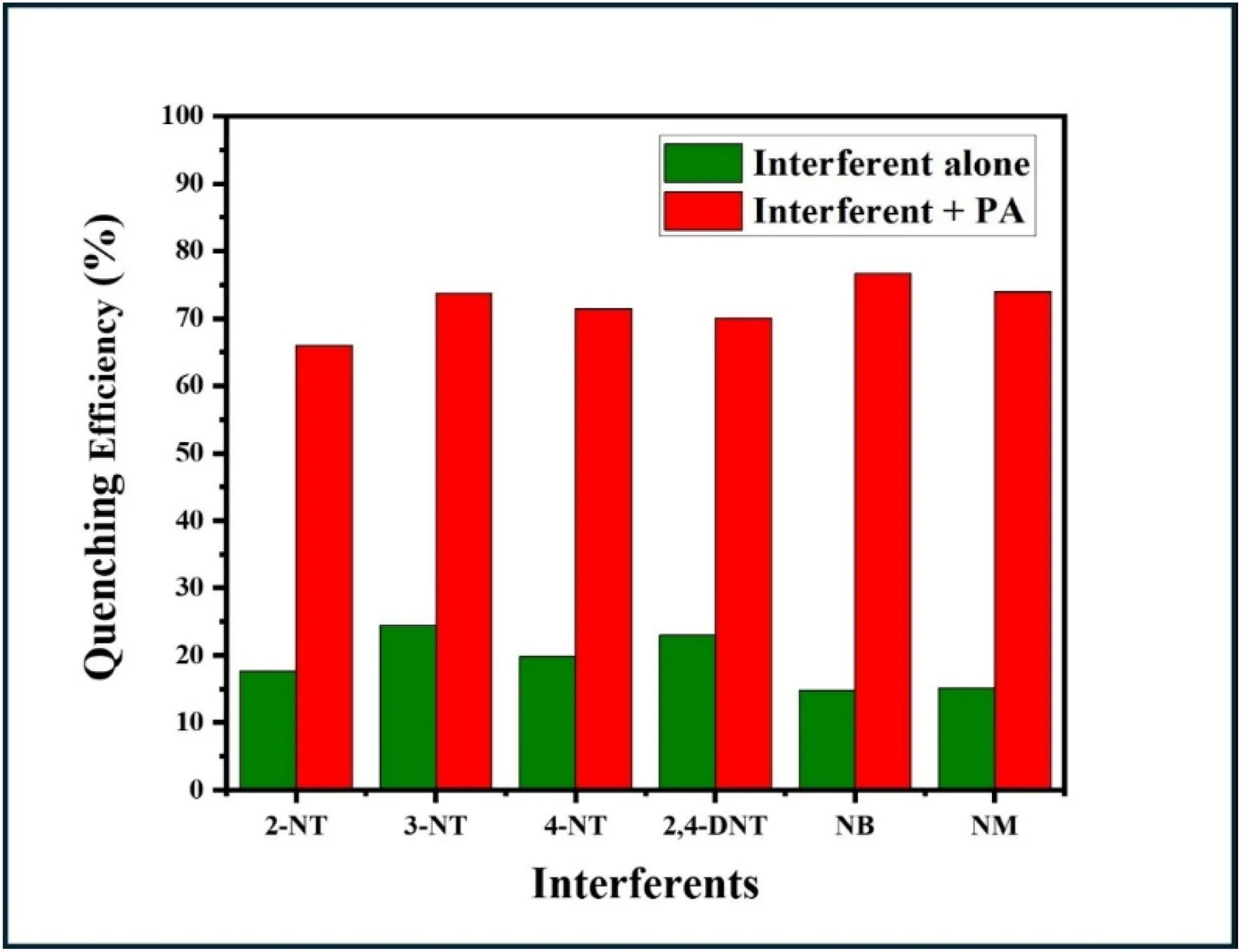
Competitive interference study of sensor (2) toward PA in the presence of potentially interfering nitro compounds. Sensor (2), PA, and each interferent were used at 1 × 10^−4^ M.

The comparatively low sensitivity of sensor 2 toward nitromethane (NM), nitrobenzene (NB), 2-nitrotoluene (2-NT), 3-nitrotoluene (3-NT), 4-nitrotoluene (4-NT), and 2,4-dinitrotoluene (2,4-DNT) was further elucidated through a systematic comparative analysis (Fig. S8–S13, in the SI file). Among all analytes examined, picric acid (PA) was identified as the most efficient fluorescence quencher for sensor 2. This pronounced preference for PA prompted a further evaluation of the sensor's selectivity under competitive conditions, where the potential influence of structurally related nitro compounds on the PA-induced fluorescence response was systematically examined. Accordingly, sensor (2), PA, and each potentially interfering species (2-NT, 3-NT, 4-NT, 2,4-DNT, NB, and NM) were employed at a concentration of 1 × 10^−4^ M. As shown in Fig. 4, the individual interferents produced only relatively minor fluorescence quenching. In sharp contrast, the addition of PA in the presence of each interferent resulted in significantly enhanced quenching efficiencies of ∼66–76%. These results demonstrate that the presence of structurally related nitro compounds has only a limited effect on the fluorescence response of sensor (2) toward PA, thereby confirming its good selectivity and robustness under competitive conditions.^39,40^ Based on these results, subsequent investigations were focused exclusively on PA.

### 3.4 pH-dependent studies on sensor (2)

To assess the intrinsic pH sensitivity of sensor (2) in the absence of PA, fluorescence measurements were performed over pH 1–6. As shown in Fig. S14 in the SI file, sensor (2) exhibited a well-defined emission band centered at ∼500 nm, with no appreciable shift in the emission maximum across the investigated pH range. These results indicate that the fluorophore's protonation state influences its emissive properties. This control study provides an important baseline for interpreting fluorescence changes induced by PA, distinguishing analyte-induced responses from intrinsic pH effects.^41,42^

To further clarify the extent to which PA-induced changes in the solution environment contribute to the observed fluorescence response, the pH of the sensor (2) solution was monitored before and after the addition of increasing concentrations of PA. The initial pH of the sensor (2) solution was 10.73 and progressively decreased to 7.54 upon increasing the PA concentration (Fig. S15 and Table S1 in the SI file), indicating that PA addition substantially modifies the acid–base environment of the sensing system. Importantly, the pH-dependent fluorescence measurements performed in the absence of PA confirmed that sensor (2) possesses an inherent pH-responsive emissive behavior. Nevertheless, the PA titration experiments revealed a consistent and pronounced decrease in fluorescence intensity with increasing PA concentration, indicating that the observed sensing response cannot be attributed solely to changes in solution pH and that direct interactions between sensor (2) and PA make a significant contribution to the fluorescence quenching. Thus, these pH measurements provide an important control for assessing the role of solution acidity/basicity and support the involvement of direct probe–analyte interactions in the fluorescence response toward PA.^40,43^

### 3.5 Practical applicability in real-water samples

Having established the contribution of both intrinsic pH effects and direct probe–PA interactions, the sensing performance of sensor (2) was subsequently evaluated in more complex aqueous matrices to assess its practical applicability. Accordingly, laboratory tap water and river water collected from the Bhima River, Maharashtra, India, were spiked with PA at concentrations ranging from 0 to 6.0 × 10^−4^ M, and the corresponding fluorescence responses were recorded. As shown in Fig. S16a and b in the SI, a progressive decrease in fluorescence intensity was observed with increasing PA concentration in both tap-water and river-water matrices. The corresponding quenching efficiencies reached 87.40% in tap water and 96.95% in river water, compared with 98.80% in methanol (Table S2 in the SI file).^4–6^ The substantial quenching observed in both aqueous matrices demonstrates that sensor (2) retains a strong fluorescence response toward PA even in the presence of potentially interfering components present in real-water samples (Table S2 in the SI file).^44–46^

**Fig. 5:**
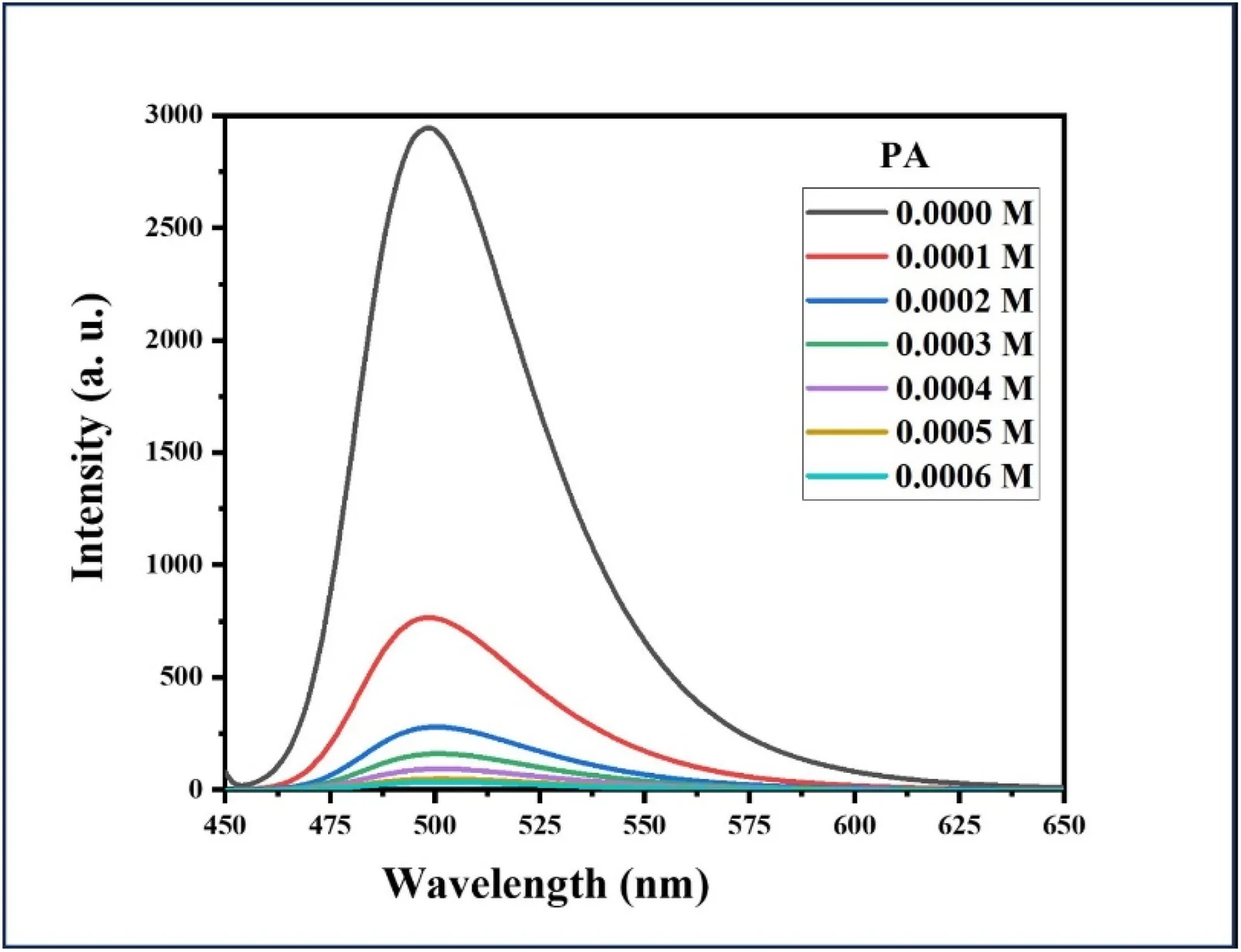
Fluorescence titration study of sensor (2) (10^−4^ M) with different amounts of PA in MeOH at *λ*_ex_ = 448 nm.

To quantitatively characterize the fluorescence response and to establish the sensing performance of sensor (2) toward PA under controlled conditions, fluorescence titration experiments were subsequently performed in methanol at 298 K. As illustrated in Fig. 5. A rapid and pronounced quenching of fluorescence intensity was observed, markedly exceeding that induced by the other tested quenchers. These results clearly demonstrate the high sensitivity and effective detection capability of sensor 2 toward picric acid in methanol.

The observed fluorescence quenching behavior can be rationalized based on two plausible mechanisms (static and collisional quenching).^47^ The first involves a conventional donor–acceptor photoinduced electron transfer (PET) process, in which electrons are transferred from the excited state of sensor (2) to the lowest unoccupied molecular orbitals (LUMOs) of the quencher. Nitro group-containing analytes are well known to be effective fluorescence quenchers due to the strong electron-withdrawing nature of the nitro groups, which substantially stabilize and lower the LUMO energy levels of the acceptor molecules. The resulting reduction in the LUMO energy facilitates efficient electron transfer from the photoexcited fluorophore, thereby promoting fluorescence quenching.^33,48,49^

**Fig. 6:**
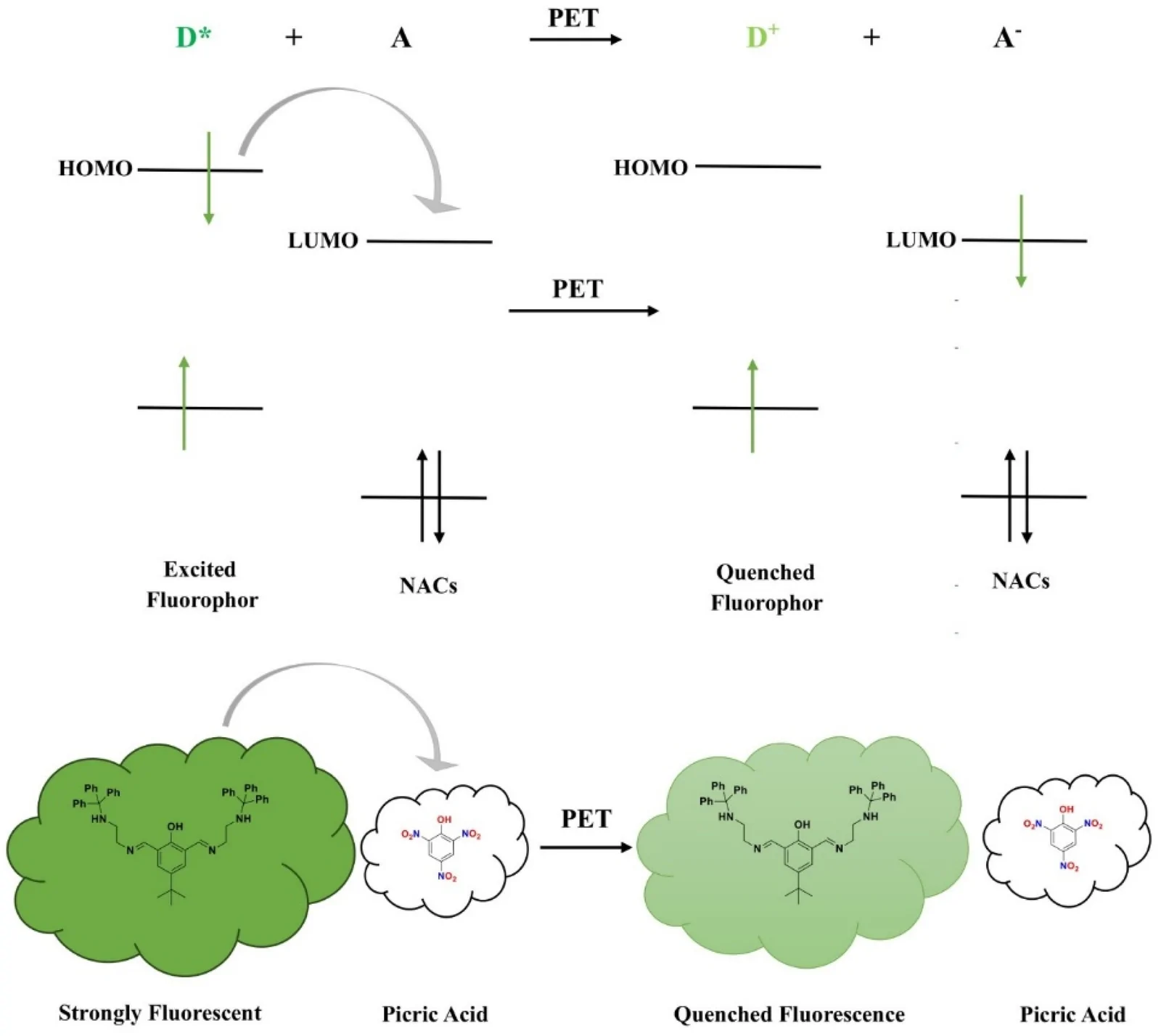
Schematic representation of photoinduced electron transfer (PET) process.

A schematic illustration of the PET mechanism is depicted in Fig. 6, where the excited fluorophore (donor, D) transfers an electron to the ground-state explosive analyte (acceptor, A). This photoinduced electron transfer pathway provides a reasonable explanation for the efficient quenching behavior observed for picric acid.^50^

### 3.6 Fluorescence response under acidic conditions

Given the importance of evaluating the sensor response under conditions that may affect its protonation state, the influence of an acidic environment on the fluorescence behavior of sensor (2) was subsequently investigated. Accordingly, additional fluorescence measurements were performed by introducing increasing concentrations of acetic acid. As shown in Fig. S17 in the SI file, the emission maximum remained centered at approximately 500 nm, whereas the fluorescence intensity exhibited moderate variations with increasing acetic acid concentration. At the highest concentration investigated (5.0 × 10^−4^ M), a discernible decrease in fluorescence intensity was observed. This behavior suggests that the photophysical response of sensor (2) is moderately sensitive to increasingly acidic conditions, which may be associated with protonation of the relevant functional groups.^51^

### 3.7 Quenching efficiency

The effective quenching of sensor (2) was investigated using the quenching efficiency (*η*) by eqn (1):^52^1
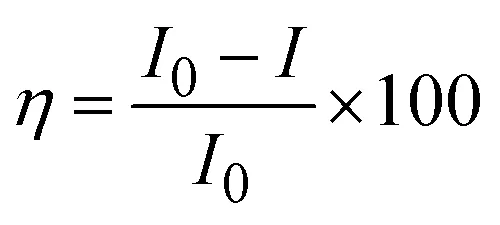


**Fig. 7:**
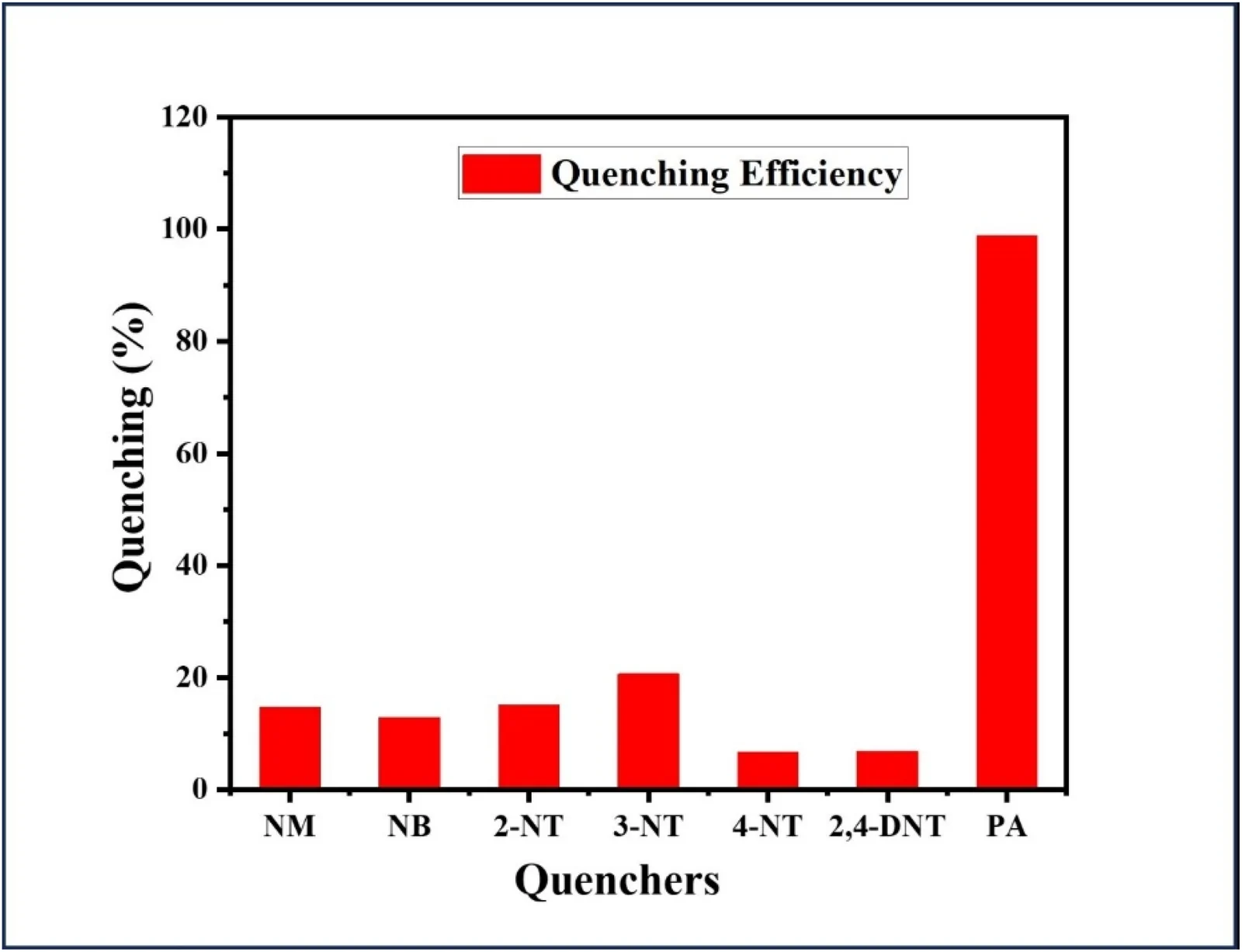
Fluorescence quenching efficiency (*η*) of sensor (2) (1 × 10^−4^ M), in MeOH toward the various analytes (5 × 10^−4^ M: NM, NB, 2-NT, 3-NT, 4-NT, 2,4-DNT and PA), calculated using eqn (1) (*λ*_ex_ = 448 nm).

The quenching efficiency (*η*) of sensor (2) by nitromethane (NM), nitrobenzene (NB), picric acid (PA), 2-nitrotoluene(2-NT), 3-nitrotoluene(3-NT), 4-nitrotoluene(4-NT), and 2,4-dinitrotoluene (2,4-DNT) is displayed in Fig. 7.

No discernible difference in fluorescence quenching efficiency was observed between aromatic and non-aromatic nitro analytes. Conspicuously, sensor (2) exhibited an exceptionally high quenching efficiency of 98.80% toward picric acid (PA) (Fig. 7), clearly establishing PA as the most effective quencher among all analytes investigated.

This pronounced selectivity can be attributed to strong electrostatic interactions between the protonated fluorophore and the picrate anion. Owing to its very low p*K*_a_ value (p*K*_a_ = 0.42), picric acid readily protonates basic sites within the fluorophore, such as amine functionalities or heterocyclic nitrogen atoms. Furthermore, PA remains predominantly in its dissociated form in methanol, thereby facilitating the formation of stable electrostatic interactions between the positively charged sensor and the picrate anion.^53,54^ These interactions bring the electron-rich fluorophore and the electron-deficient picrate anion into proximity, significantly enhancing the efficiency of electron or energy transfer processes and, consequently, the sensitivity of the probe toward PA.^55,56^

**Scheme 2:**
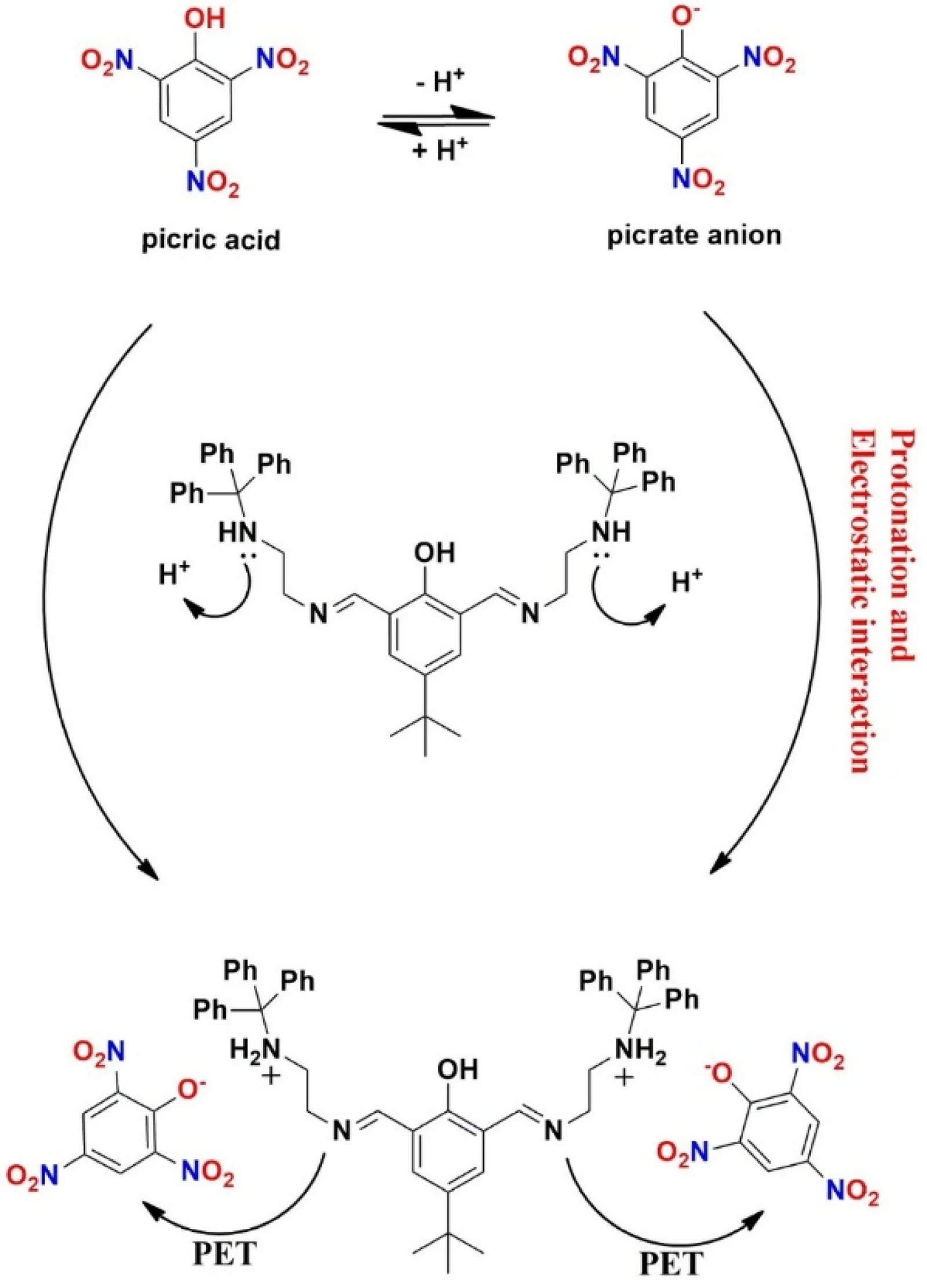
Schematic representation of protonation and electrostatic interaction.

A generalized mechanistic pathway underlying the selective recognition of picric acid by such fluorophores is illustrated in Scheme 2. In contrast, other nitroaromatic analytes exhibit relatively inefficient electron or energy transfer, primarily due to their weaker acidity and limited ability to protonate the basic sites of the fluorophore.^53^

### 3.8 Fluorescence lifetime measurements

A clear understanding of the underlying quenching processes is essential for elucidating the sensing behavior of fluorescent materials. Static and collisional quenching mechanisms can be differentiated using time-resolved fluorescence measurements by monitoring the decay profiles of the sensing material at varying quencher concentrations.^52^ In the case of static quenching, the fluorescence lifetime remains unchanged despite increasing quencher concentration. In contrast, collisional quenching introduces an additional non-radiative relaxation pathway, leading to a measurable decrease in fluorescence lifetime.^57–60^

**Fig. 8:**
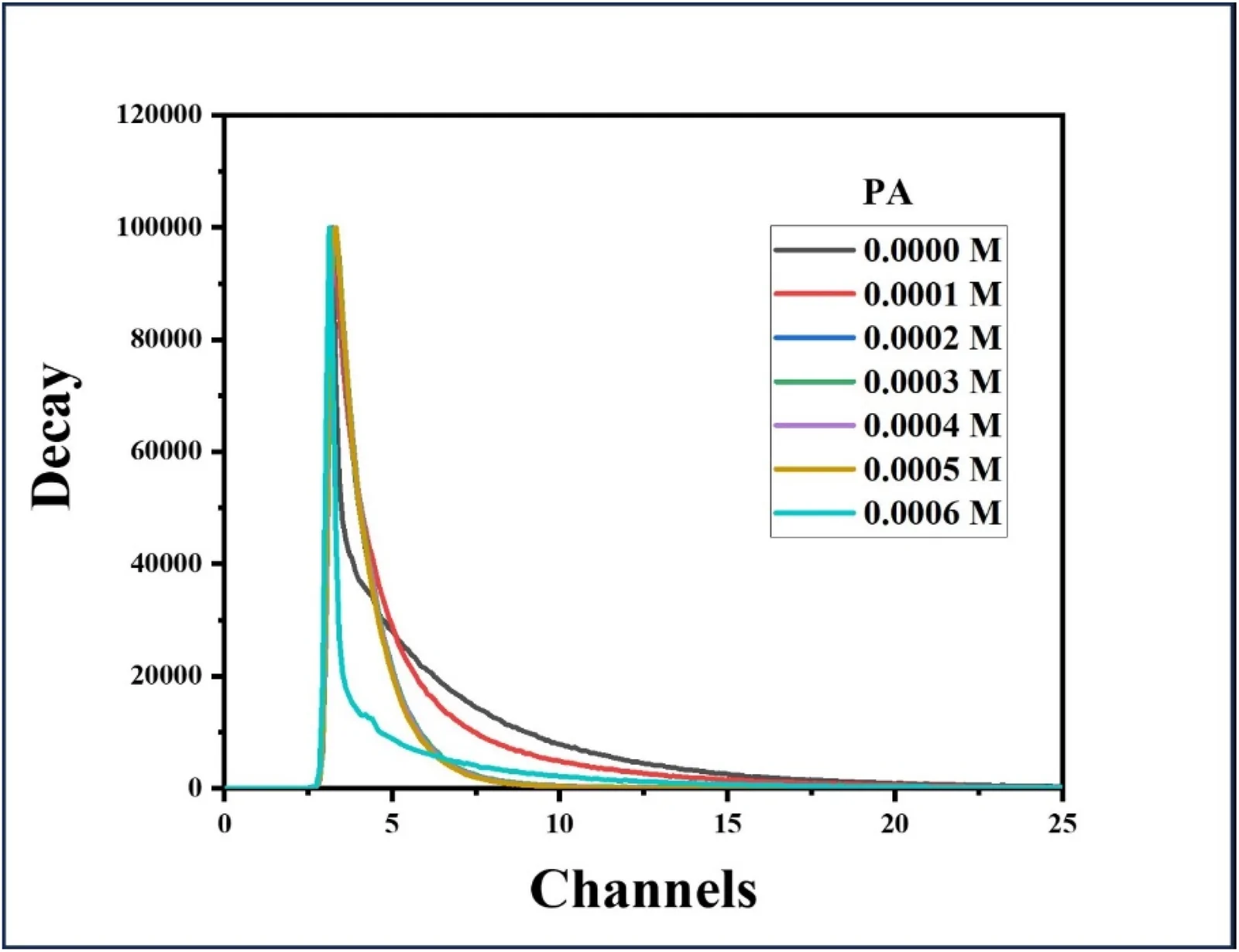
Fluorescence decay profiles of sensor (2) (1 × 10^−4^ M), in MeOH with increasing picric acid (0–6 × 10^−4^ M); *λ*_ex_ = 448 nm, *λ*_em_ = 500 nm. Decays were fit to a bi-exponential model (parameters in Table S3 in the SI file).

To confirm the quenching mechanism, time-resolved fluorescence quenching experiments were carried out at room temperature in the presence of PA at different concentrations ranging from 1 × 10^−4^ M to 6 × 10^−4^ M. The corresponding decay curves are presented in Fig. 8. All fluorescence decay profiles were best fitted using a bi-exponential decay model. The extracted fluorescence lifetime parameters are summarized in Table S3 in the SI file.

A decrease in fluorescence lifetime is observed with increasing quencher concentration (Fig. 8), indicating a significant dynamic (collisional) contribution to the quenching; as shown in Section 3.8, however, the magnitude of the quenching parameters reveals that a static, ground-state component also operates.^14,60^

### 3.9 Steady-state Stern–Volmer plot analysis

**Fig. 9:**
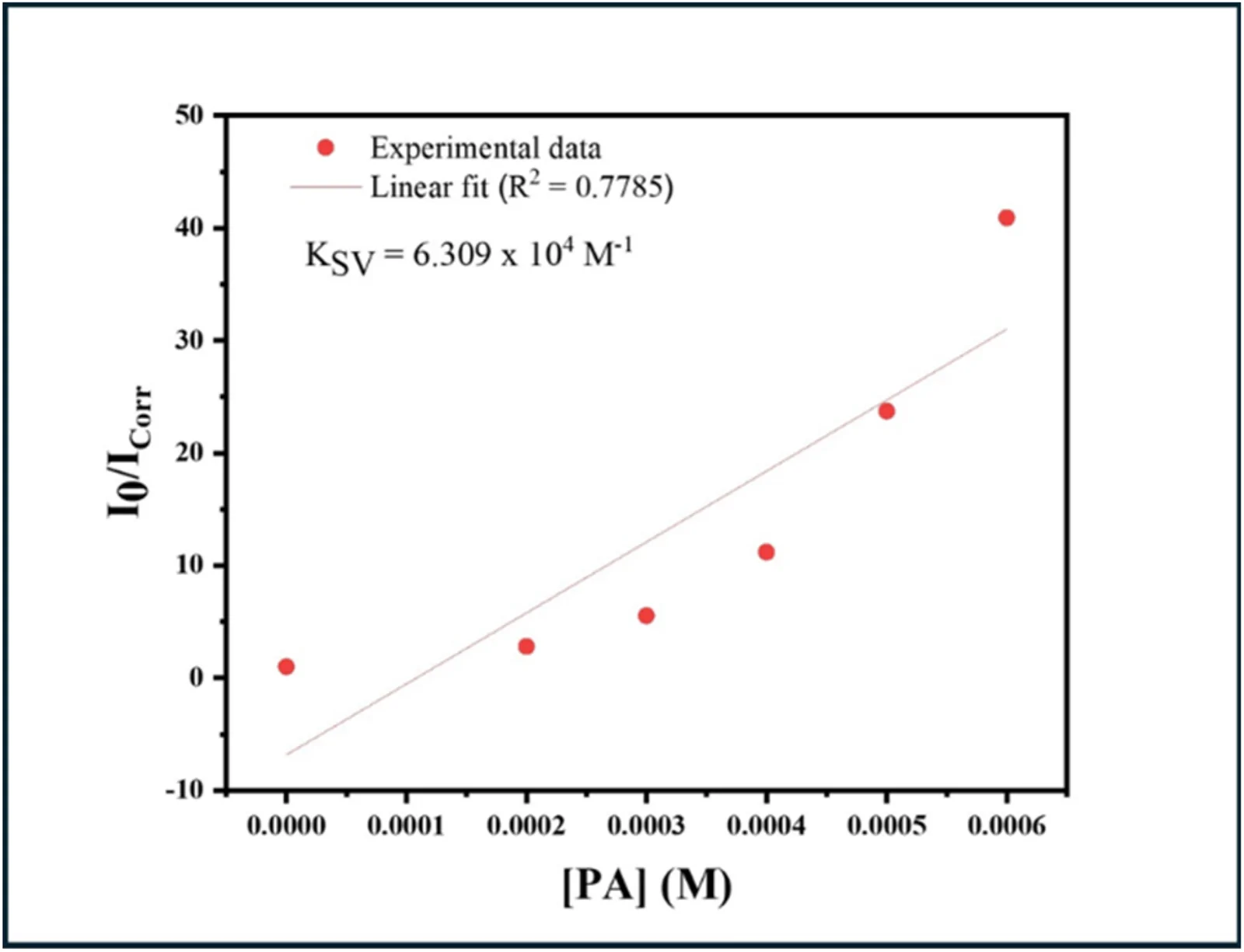
Steady-state Stern–Volmer plot of sensor (2) luminophores with picric acid.

To elucidate the quenching processes operative in sensor (2), Stern–Volmer (S–V) analysis was performed for picric acid by plotting the fluorescence intensity ratio (*I*_0_/*I*) as a function of quencher concentration [Q] (eqn (2)).^52^2
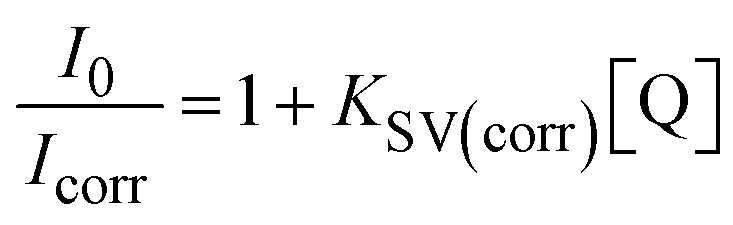
where, [Q] denotes the concentration of the quencher, *K*_SV_ is the Stern–Volmer constant, *I*_0_ represents the emission intensity of sensor (2) in the absence of quencher, and *I* corresponds to the emission intensity in the presence of the quencher. The Stern–Volmer constant (*K*_SV_) was determined from the slope of the initial, low-[PA] linear region of the *I*_0_/*I versus* [Q] plot and is related to the quenching parameters through *K*_SV_ = *k*_q_·*τ*_0_, where *k*_q_ is the Stern–Volmer quenching rate constant and *τ*_0_ is the intrinsic fluorescence lifetime of sensor (2) in the absence of quencher. The corresponding Stern–Volmer plots are presented in Fig. 9.^58,61,62^

The fluorescence quenching rate parameter (*k*_q_) was estimated using eqn (3) (ref. 52) and the experimental values of *K*_SV_ and *τ*_0_.3
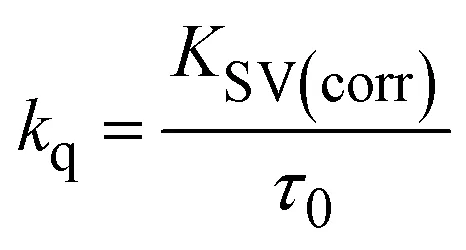
where *τ*_0_ is the fluorophore's lifetime in absence of quencher. The Stern–Volmer plot's slope was used to compute the Stern–Volmer quenching constant. The quenching constant (*K*_SV_) for sensor (2) was found to be 6.309 × 10^4^ M^−1^, indicating the sensor's high sensitivity to PA. Using the intrinsic lifetime (*τ*_o_) of 1.08 ns, the bimolecular quenching rate constant (*k*_q_) was calculated from eqn (3) as 5.8 × 10^13^ M^−1^ s^−1^. This value is roughly three orders of magnitude larger than the diffusion-controlled limit in fluid solution (≈10^10^ M^−1^ s^−1^),^38^ which indicates that the quenching cannot be purely dynamic (collisional) and that a substantial static (ground-state) component operates. Consistent with this, the steady-state Stern–Volmer plot shows an upward (positive) curvature, whereas a purely dynamic process would give a linear plot; such positive deviations are characteristic of combined static-and-dynamic (sphere-of-action) quenching.^57–65^ Taken together with the ground-state association inferred from the Benesi–Hildebrand analysis (Section 3.10) and the decrease in fluorescence lifetime (Section 3.8), the data indicate that PA is recognized through a combination of (i) ground-state ion-pair (charge-transfer) complex formation between the protonated sensor and picrate and (ii) excited-state, PET-driven collisional quenching. A primary/secondary inner-filter contribution arising from the strong absorption of PA at the excitation (448 nm) and emission (500 nm) wavelengths may further augment the apparent quenching and should be evaluated by absorbance-based correction.^61–63,66,67^

### 3.10 Benesi–Hildebrand analysis

Using the Benesi–Hildebrand analysis study, the association constant and binding stoichiometry were estimated. Eqn (4) was used to calculate the binding stoichiometry and association constants based on the fluorescence spectral changes, as detailed in the literature.^52,68,69^

4 1/(*F*_0_ − *F*_icorr_) = 1/{*K*_a_ × (*F*_0_ − *F*_Min_) × [Q]} + 1/(*F*_0_ − *F*_Min_)

**Fig. 10:**
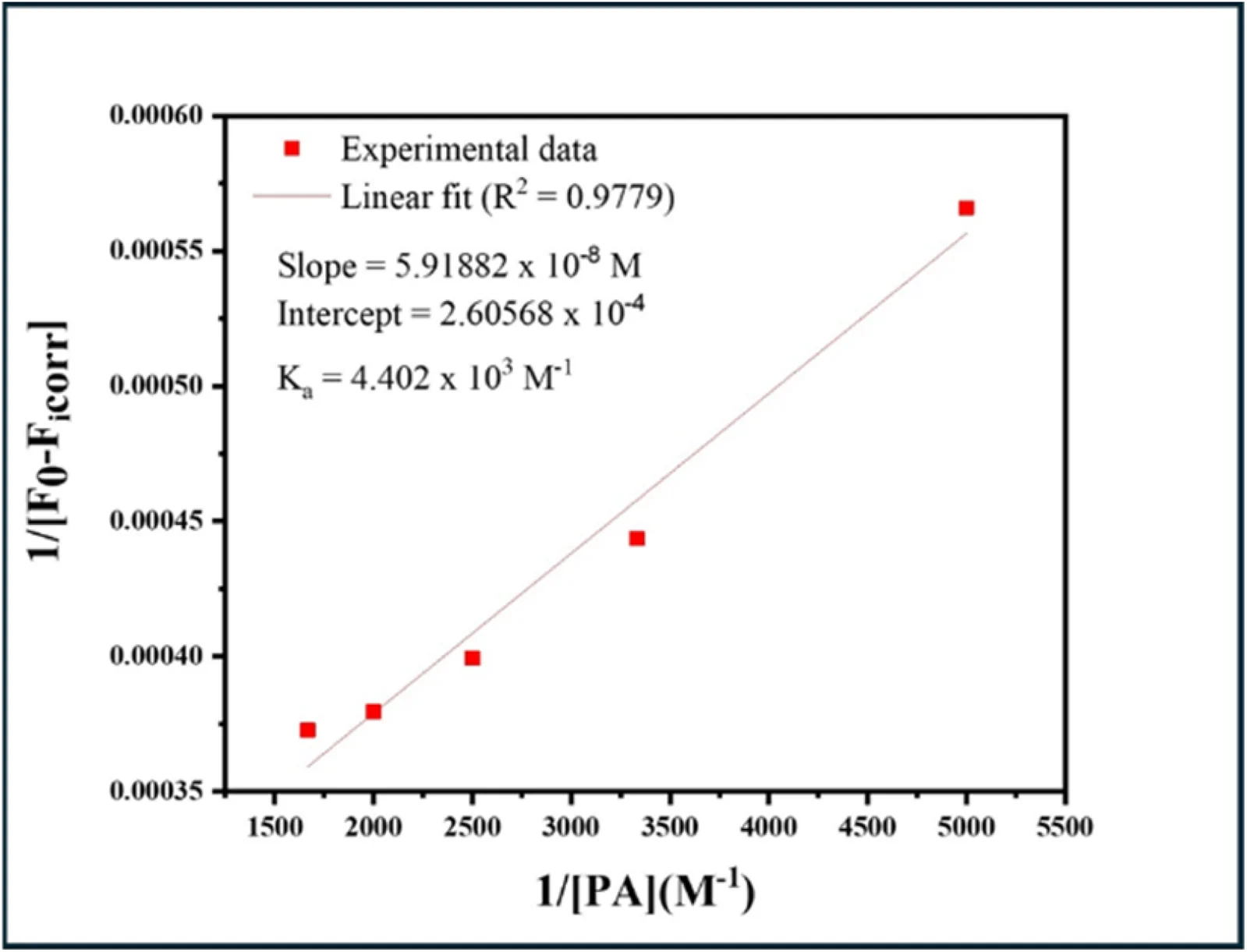
Benesi–Hildebrand plot of sensor (2) in response to PA.

Here, *F*_Min_ represents the fluorescence intensity recorded in the presence of an excess of quencher, *F*_i_ corresponds to the fluorescence intensity measured at varying quencher concentrations, and *F*_0_ denotes the intrinsic fluorescence intensity of the fluorophore. The binding stoichiometry between the fluorophore and the quencher was elucidated from the Benesi–Hildebrand plot of 1/(*F*_0_ − *F*_i_) *versus* 1/[Q] (Fig. 10; note the plot is linear only in the low-1/[Q] region and deviates at high 1/[Q], consistent with non-simple 1 : 1 binding) 1/(*F*_0_ − *F*_i_)1/[Q]. This analysis was further employed to evaluate the association constant of the fluorophore–quencher complex. From the Benesi–Hildebrand plot, the association constant (*K*_a_) for the sensor–picrate complex was calculated to be *K*_a_ = 4.402 × 10^3^ M^−1^. The measurable association constant confirms ground-state complexation between PA and the sensor, corroborating the static contribution to quenching identified in Section 3.9.

### 3.11 Job's plots analysis

**Fig. 11:**
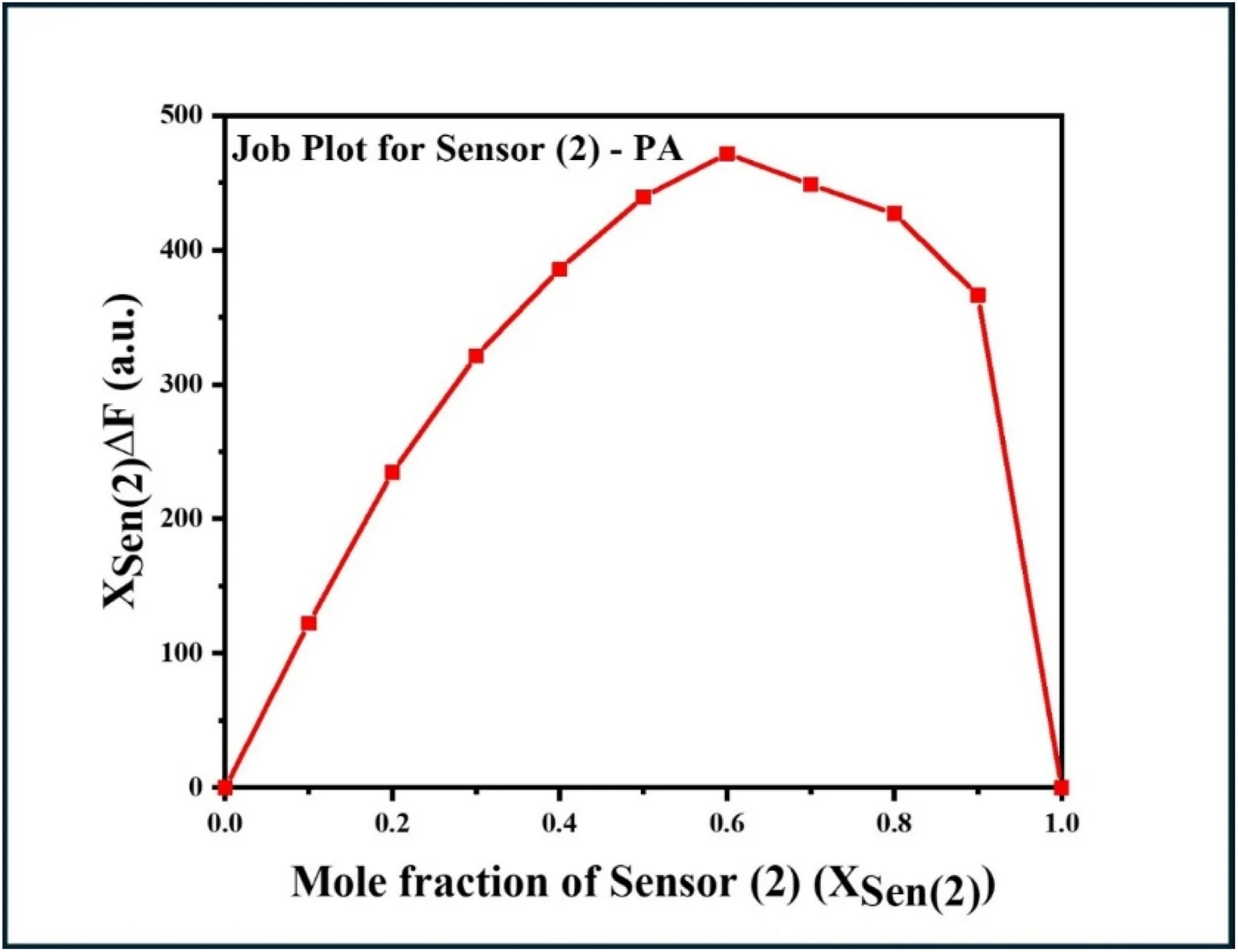
Job's plots for the sensor (2)–PA systems constructed by plotting *X*_Sen(__2__)_Δ*F* against the mole fraction of sensor (2) (*X*_Sen(__2__)_).

The binding stoichiometry between sensor (2) and picric acid (PA) was investigated using the method of continuous variation (Job's plot). Equimolar stock solutions of sensor (2) and PA, each having a concentration of 1.0 × 10^−5^ M, were mixed in different volume ratios while maintaining a constant total volume of 5.0 mL. Thus, the total concentration of sensor (2) and PA remained constant throughout the experiment. The change in fluorescence intensity was calculated using the eqn (5):

5 Δ*F* = *F*_0_ − *F*

where *F*_0_ is the fluorescence intensity of sensor (2) in the absence of PA, and *F* is the fluorescence intensity of the sensor (2)–PA mixture. The Job's plot was constructed by plotting *X*_Sen(__2__)_Δ*F* against the mole fraction of sensor (2) (*X*_Sen(__2__)_). The plot exhibited a broad maximum in the *X*_Sen(__2__)_ = 0.5–0.6 region (Fig. 11 and Table S4 in the SI file), indicating that the interaction between sensor (2) and PA is predominantly consistent with a 1 : 1 binding stoichiometry. The slight shift of the observed maximum from the ideal mole fraction of 0.5 may be attributed to experimental uncertainty, the relatively large mole-fraction interval, and the broad nature of the fluorescence response. Therefore, the Job's plot supports the assumption of 1 : 1 complex formation between sensor (2) and PA used in the Benesi–Hildebrand analysis.^39,70,71^

To further corroborate complex formation, ESI-MS analysis was conducted, revealing spectral signatures consistent with the presence of a probe–PA adduct (Fig. S18a–c in the SI file) and thereby lending additional support to the proposed 1 : 1 binding interaction. Collectively, the convergent evidence from the Job's plot, ESI-MS analysis, and Benesi–Hildebrand treatment substantiates the assigned 1 : 1 stoichiometry of the probe–PA complex.

### 3.12 Chemical stability of sensor (2) upon PA addition

To evaluate the chemical robustness of sensor (2) throughout the PA-sensing process, UV-vis absorption spectra were acquired both before and after PA addition. As depicted in Fig. S19 in the SI file, progressive addition of PA induced changes in the intensity and profile of the absorption bands; however, the core spectral signatures characteristic of the probe scaffold remained intact, with no indication of wholesale breakdown of the chromophore. These spectral alterations are thus attributed predominantly to probe–PA interaction and the consequent perturbation of the electronic environment, rather than to structural degradation. Collectively, these findings indicate that the Schiff-base sensing platform retains adequate stability under the experimental conditions employed, and that the observed fluorescence response toward PA originates chiefly from probe–PA association rather than decomposition of the sensor.^72,73^

### 3.13 Determination of the limit of detection (LOD)

To gain deeper insight into the quenching behavior, the limits of detection (LODs) for the investigated analytes were determined from the slopes of the calibration plots derived from the linear dependence of the fluorescence intensity of sensor (2) at 500 nm on the corresponding analyte concentrations. The detection limits were evaluated from fluorescence titration experiments, with the standard deviation of the blank (*σ*) obtained from 15 independent measurements of the fluorescence emission spectrum of sensor (2) in methanol. Calibration curves were constructed by plotting the emission intensity at 500 nm as a function of analyte concentration, and the LOD values were subsequently calculated using eqn (5):^51,52,68,69^

6 Detection limit = 3 × *σ*/*K*

The limit of detection (LOD) was calculated using the eqn (6), where *σ* is the standard deviation of 15 repeated blank fluorescence measurements and *S* is the slope of the Stern–Volmer calibration plot. The normalized standard deviation of the blank response was 0.01589, while the slope was 6.309 × 10^4^ M^−1^. The LOD was calculated to be 7.56 × 10^−7^ M (0.756 µM).

The resulting micromolar-level LOD is comparable to that of several reported small-molecule PA probes (Table 1), while the present sensor offers a simple, high-yield synthesis and a convenient solid-state readout.

**Table 1:** Compares the picric-acid sensing performance of sensor (2) with representative fluorescent PA probes reported in the literature

Entry	Sensor/probe	Solvent/medium	LOD (µM)	*K*_SV_ (M^−1^)	Reference
1	Sensor (2)	Methanol	0.756	6.309 × 10^4^	This work
2	Fluorescent probe	Aqueous/organic	2.18	4.3 × 10^3^	74
3	Pyrene Schiff base	DMF/water	0.162	9.2 × 10^3^	75
4	Organic chromophore	Acetonitrile	19.0	9.9 × 10^6^	76
5	Pyrene-based fluorescent probe	Aqueous buffer	0.87	—	77
6	BODIPY fluorescent probe	THF/water	0.131	2.1 × 10^6^	78
7	Polymer-based sensor	Organic solvent	0.0018	—	79
8	Pyrene based chemosensor	Aqueous	0.063	—	80
9	Polymer nanoparticles	Interface	0.026	—	81
10	Triphenylamine sensor	THF	0.20	—	82
11	Coordination complex	H_2_O/THF	0.0344	1.92 × 10^5^	83

### 3.14 Visual detection of PA in solution under UV illumination

**Fig. 12:**
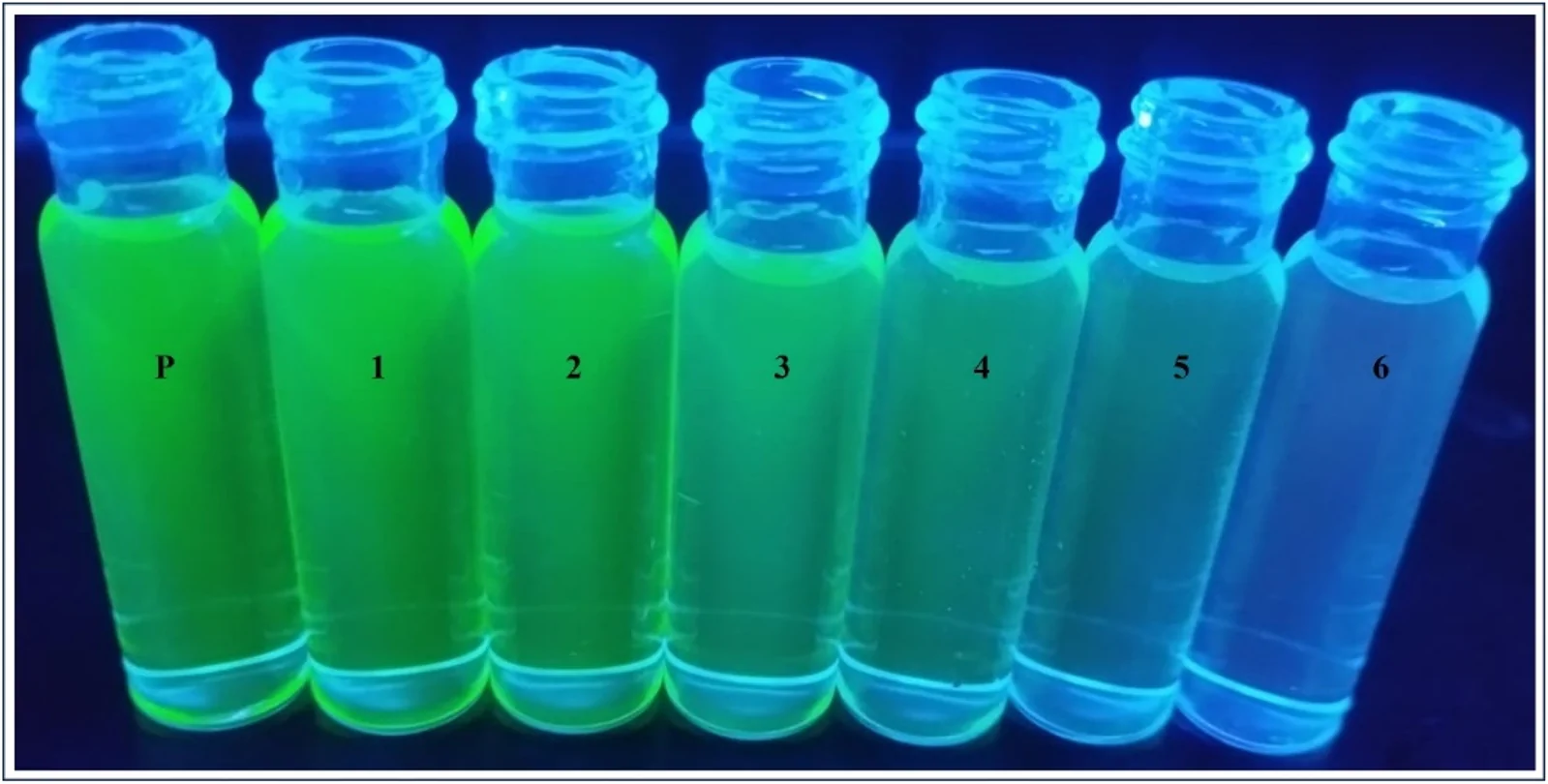
Visual color changes of sensor (2) are observed with increasing quantity of PA (P = pure sensor; 1–6 = increasing PA concentration) in MeOH, from left to right, under 365 nm UV light. [PA concentrations for 1–6 are 1 × 10^−4^, 2 × 10^−4^, 3 × 10^−4^, 4 × 10^−4^, 5 × 10^−4^, and 6 × 10^−4^ M, respectively].

Rapid and visual detection is essential for enhancing the practical applicability of the probe as a portable sensing platform.^2^ Accordingly, visual detection of picric acid (PA) was investigated in methanolic solution, where the addition of PA to sensor (2) induced a distinct color change under UV irradiation (Fig. 12), enabling straightforward naked-eye recognition.

### 3.15 Time-dependent fluorescence measurements

**Fig. 13:**
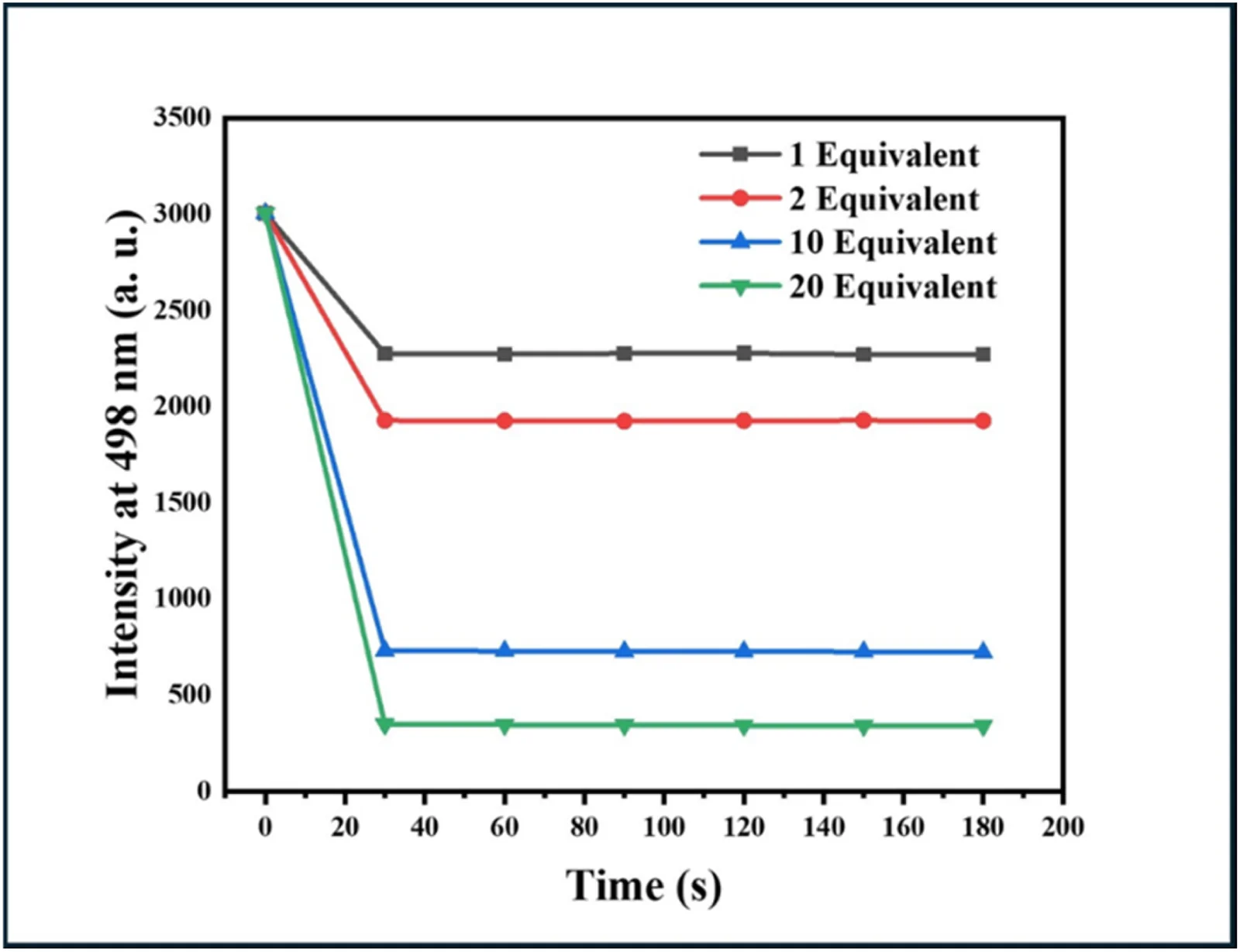
Time-dependent fluorescence response of sensor (2) (1 × 10^−4^ M) toward PA at different concentrations 1, 2, 10, and 20 equivalents.

The response time is a critical parameter for evaluating the sensing performance of a fluorescence probe. The time-dependent fluorescence response of sensor (2) toward PA was investigated at different PA concentrations (1, 2, 10, and 20 equivalents). As shown in Fig. 13, a rapid decrease in fluorescence intensity was observed immediately after the addition of PA, and the fluorescence response reached a nearly constant value within 30 s for all investigated concentrations. This indicates that the sensor (2) detection of PA was extremely rapid process.^84^

### 3.16 Paper-based sensors for the visual detection of picric acid

**Fig. 14:**
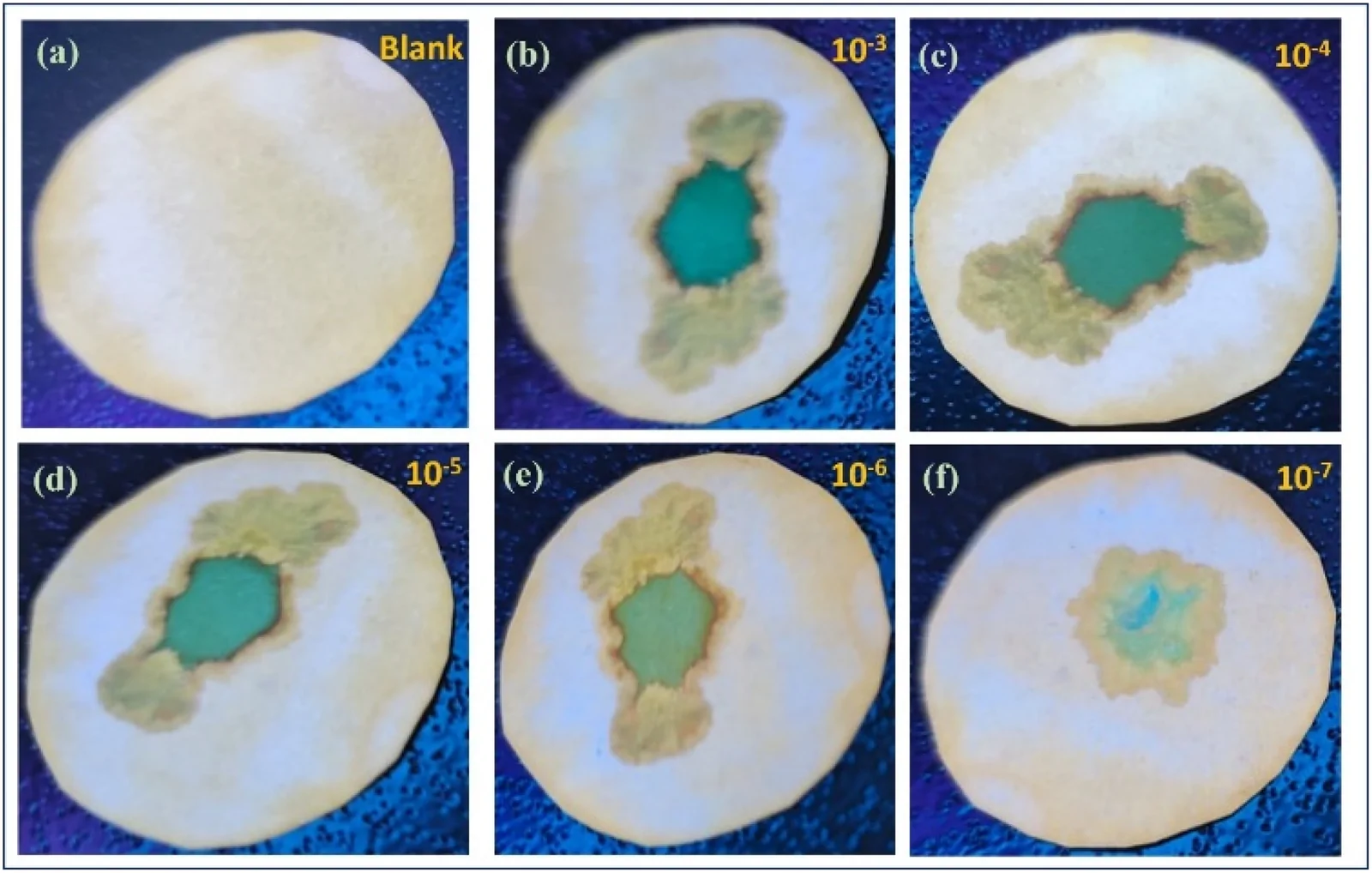
Fluorescence images (under 365 nm UV) of Whatman filter-paper discs impregnated with sensor (2): (a) blank, and (b–f) loaded with picric acid at 10^−3^, 10^−4^, 10^−5^, 10^−6^ and 10^−7^ M, respectively.

To evaluate the practical applicability of sensor (2) for explosive detection, paper-strip-based solid-state sensing experiments were conducted. Whatman filter paper strips were impregnated with a dichloromethane (DCM) solution of sensor (2) (0.2 mg mL^−1^) and allowed to dry completely.^85^ The fluorescence response of the resulting strips was examined under 365 nm UV irradiation, and the blank strip exhibited a uniform emission, as shown in Fig. 14a. Owing to its homogeneous brownish-yellow fluorescence without visible defects, the sensor (2)-coated paper was selected for solid-state picric acid (PA) detection. PA solutions with concentrations ranging from 10^−3^ to 10^−7^ M were prepared, and 5 µL aliquots of each solution were drop-cast onto the paper strips and dried to assess fluorescence quenching. The formation of darkened regions on the sensor-coated paper, corresponding to quenched emission, is clearly observed in Fig. 14b–f. Notably, a discernible quenched spot was detected even at a PA concentration as low as 10^−7^ M, highlighting the qualitative sensitivity of the sensor toward PA in the solid state. This solid-state value represents the lowest PA concentration at which quenching of the drop-cast spot could be visually discerned under UV illumination; it is therefore an operational (qualitative) detection limit and is not directly comparable with the calibrated solution-phase LOD (0.756 µM) obtained from the 3*σ*/slope method. The solid-state limit was assessed by image/intensity analysis. These results underscore the potential of the synthesized AIEE-active sensor (2) for practical, on-site explosive detection applications.^2,86–88^

### 3.17 Aggregation induced enhanced emission (AIEE)

**Fig. 15:**
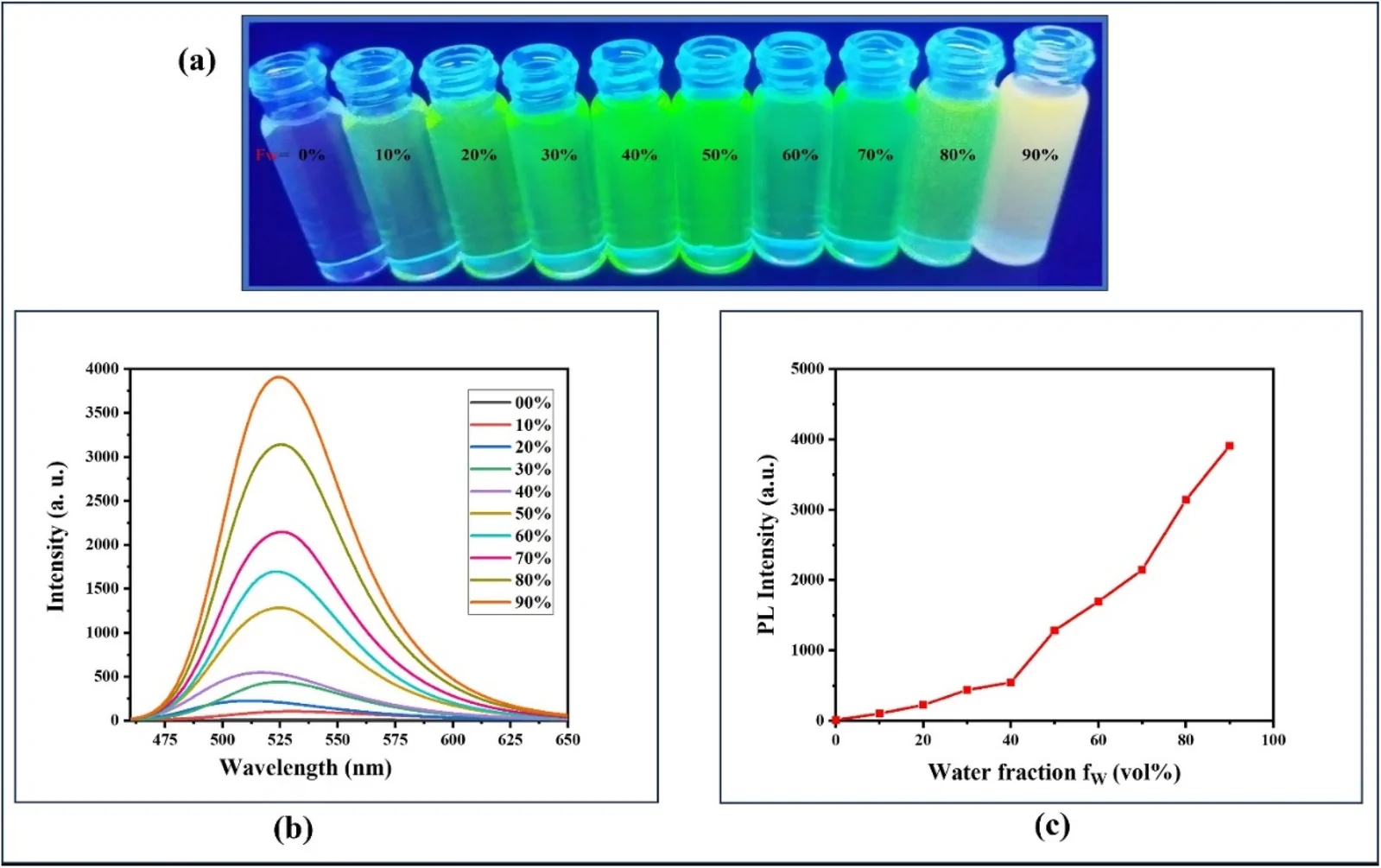
(a) Luminescence images of sensor (2) (irradiated at 365 nm by UV lamp) in water/MeOH mixed solvents; (b) PL spectra of sensor (2) in water/MeOH mixed solvents with different water fractions (*f*_w_) *λ*_ex_ = 448 nm, and (c) the changes of the PL peak intensity with different water fraction, (*f*_w_).

The aggregation induced enhanced emission (AIEE) behavior of sensor (2) was systematically investigated using water/methanol binary solvent mixtures, taking advantage of its good solubility in methanol and poor solubility in water. A series of photoluminescence (PL) measurements was performed by gradually increasing the water fraction in the solvent system. Two prominent features were observed. First, a gradual red shift in the emission maximum from ≈515 to 524 nm occurred as the water content increased from 0 to 90%. Second, a pronounced enhancement in emission intensity was recorded with increasing water fraction, reaching a maximum at 90% water content (Fig. 15). To elucidate the underlying mechanisms responsible for these changes in PL behavior, a range of complementary experiments was subsequently conducted.

Because sensor (2) is appreciably emissive even when molecularly dissolved in dilute methanol, the emission enhancement on increasing the water fraction is more accurately described as aggregation induced enhanced emission (AIEE) than as classical AIE.

**Fig. 16:**
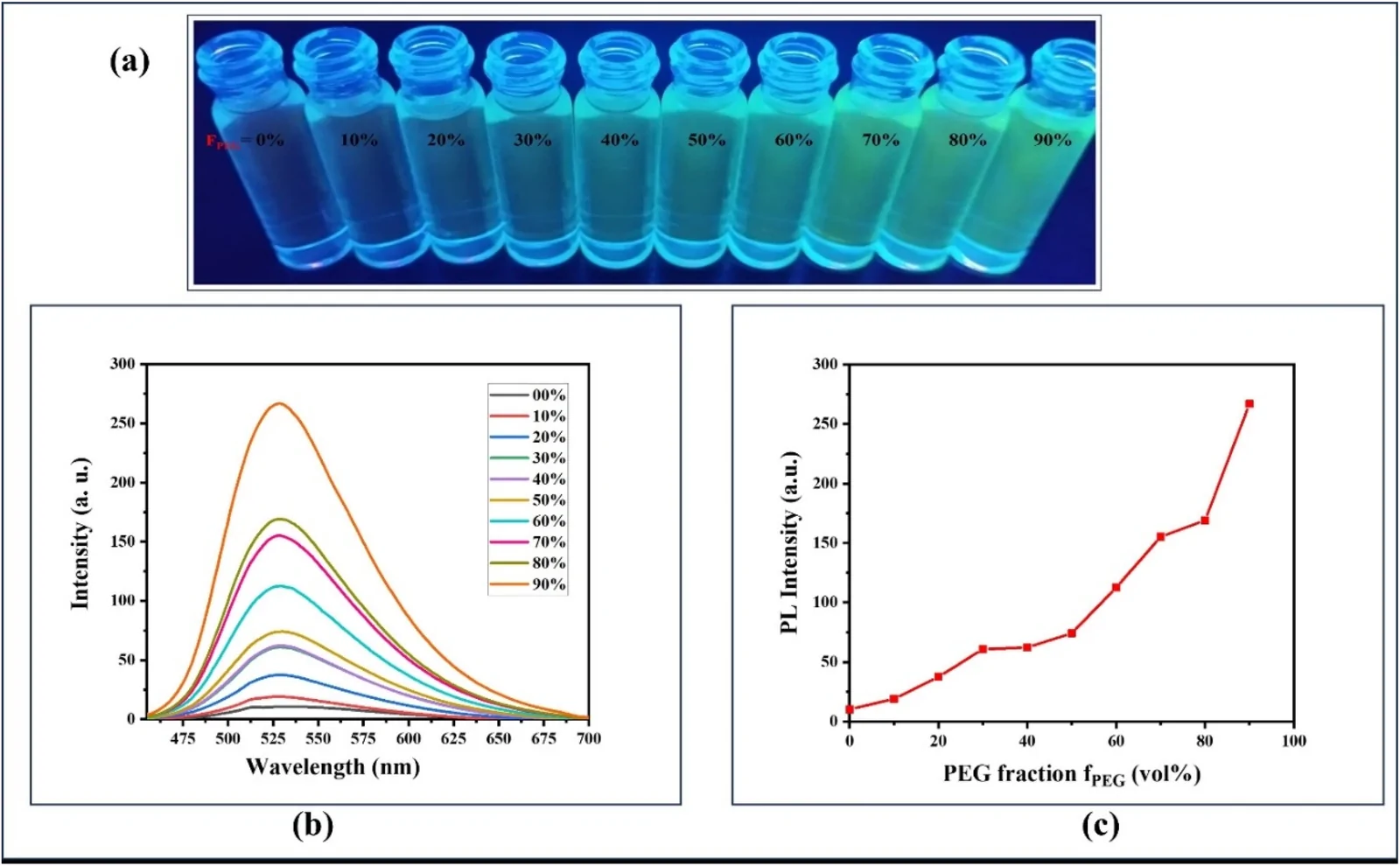
(a) Luminescence images of sensor (2) (irradiated at 365 nm by UV lamp) in MeOH/PEG mixed solvents; (b) PL spectra of sensor (2) in MeOH/PEG mixed solvents with different PEG fractions (*f*_PEG_) *λ*_ex_ = 448 nm, and (c) the changes of the PL peak intensity with different *f*_PEG_.

To further probe the origin of the emission enhancement, a series of solutions was prepared using binary mixtures of methanol and polyethylene glycol (PEG), a high-viscosity solvent, with PEG content varied from 0 to 90% while maintaining a constant methanol fraction (Fig. 16).^35,89^ A progressive increase in emission intensity was observed with increasing PEG concentration. This behavior indicates that restricted intramolecular rotation (RIR) plays a dominant role in governing the AIEE activity of sensor (2).^90^ In particular, the rotational freedom of the phenyl moieties is significantly suppressed in the viscous medium, effectively blocking non-radiative decay pathways and leading to enhanced radiative emission. The pronounced fluorescence enhancement is further supported by the presence of multiple short-range intermolecular interactions, which collectively contribute to the restriction of molecular motions in the aggregated state.^91–96^ The dynamic light scattering (DLS) analysis of sensor (2) in MeOH/H_2_O, with water fraction (*f*_w_) of 10% and 90%, discloses average aggregate sizes of approximately 354 days per nm and 820 days per nm, respectively (Fig. S21 in the SI file).

### 3.18 Computational details

**Fig. 17:**
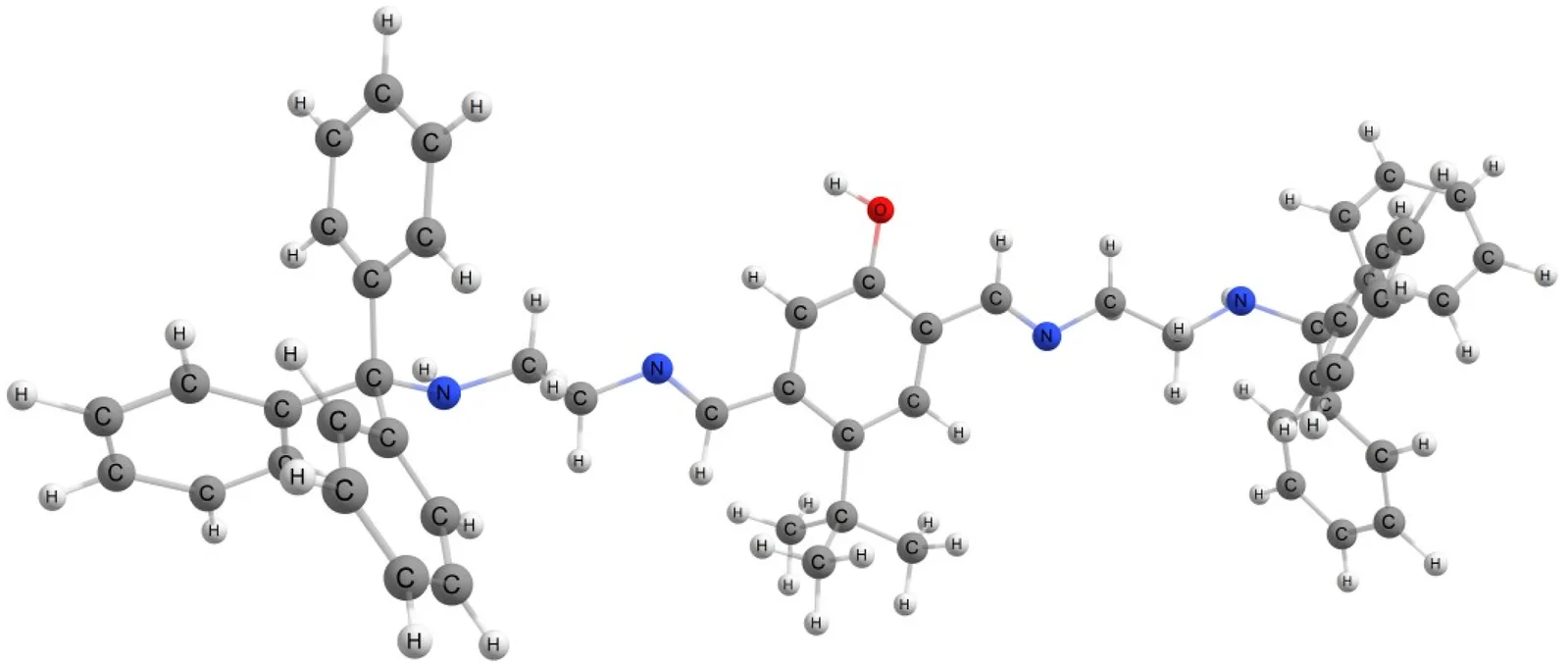
Optimized structure of sensor (2) at B3LYP/def2-SVP level of theory.

The structure of 4-(*tert*-butyl)-2-((*E*)-((2-(tritylamino)ethyl)imino)methyl)-6-((*E*)-((2-(tritylamino)ethyl)imino)methyl)phenol (2) was visualized using Avogadro Program Package^97^ and was auto-optimized through molecular mechanics (MM) using the same package. Density Functional Theory (DFT) ground state optimization with proper frequency checks was performed using the B3LYP hybrid functional.^98,99^ With the basis set def2-SVP in the solvent methanol (CPCM solvation model).^100^ The optimized structure of the molecule is shown in Fig. 17 and the optimized coordinates are shown in the Table S6 in the SI file.

**Fig. 18:**
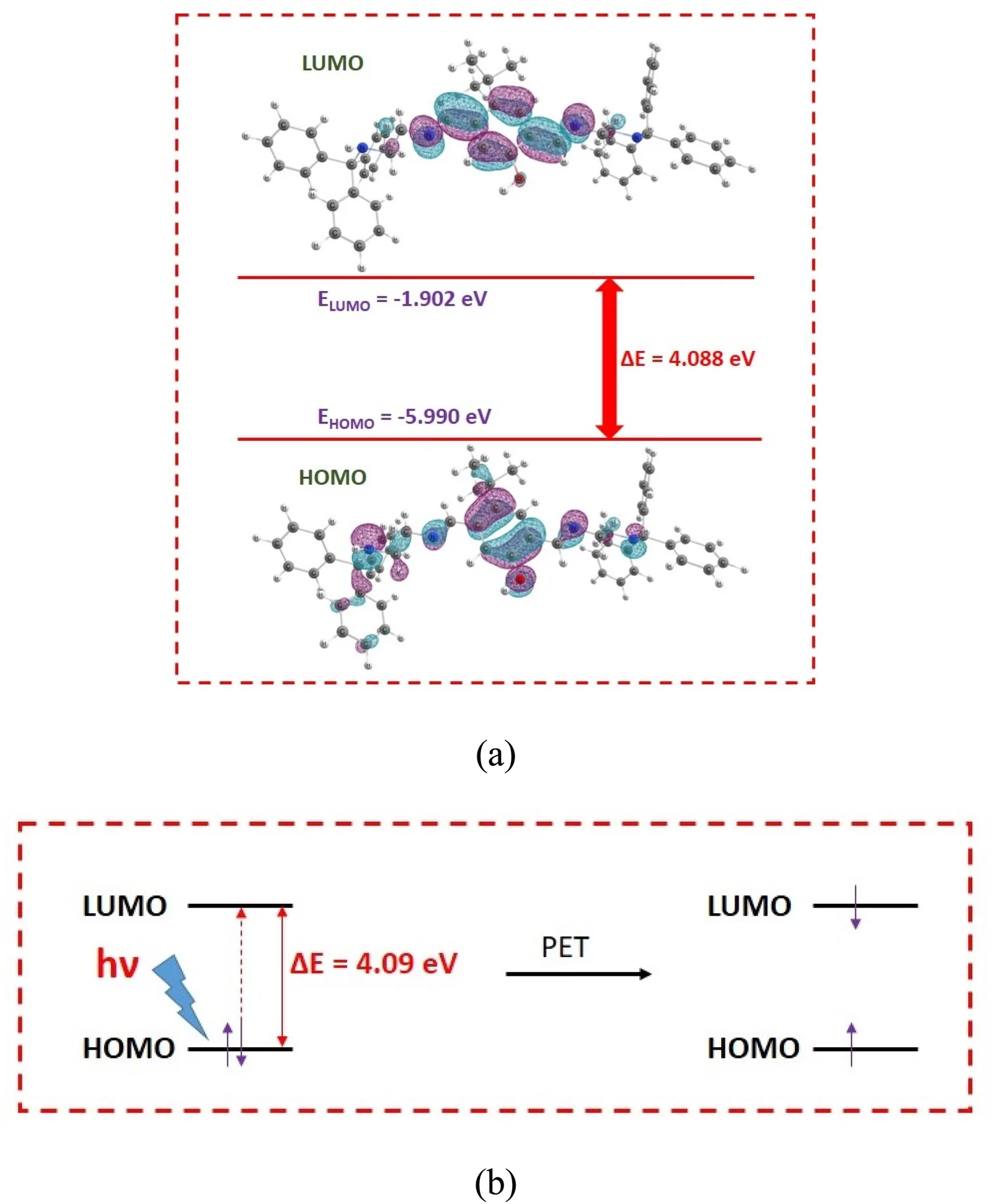
(a) Isodensity plot representing HOMO and LUMO of sensor (2) along with their energy values at B3LYP/def2-SVP level of theory, (b) HOMO → LUMO electron transfer showing PET.

The Frontier Molecular Orbitals (FMOs) of the molecule, along with their corresponding energy values and the HOMO–LUMO energy gap are presented in Fig. 18.

**Fig. 19:**
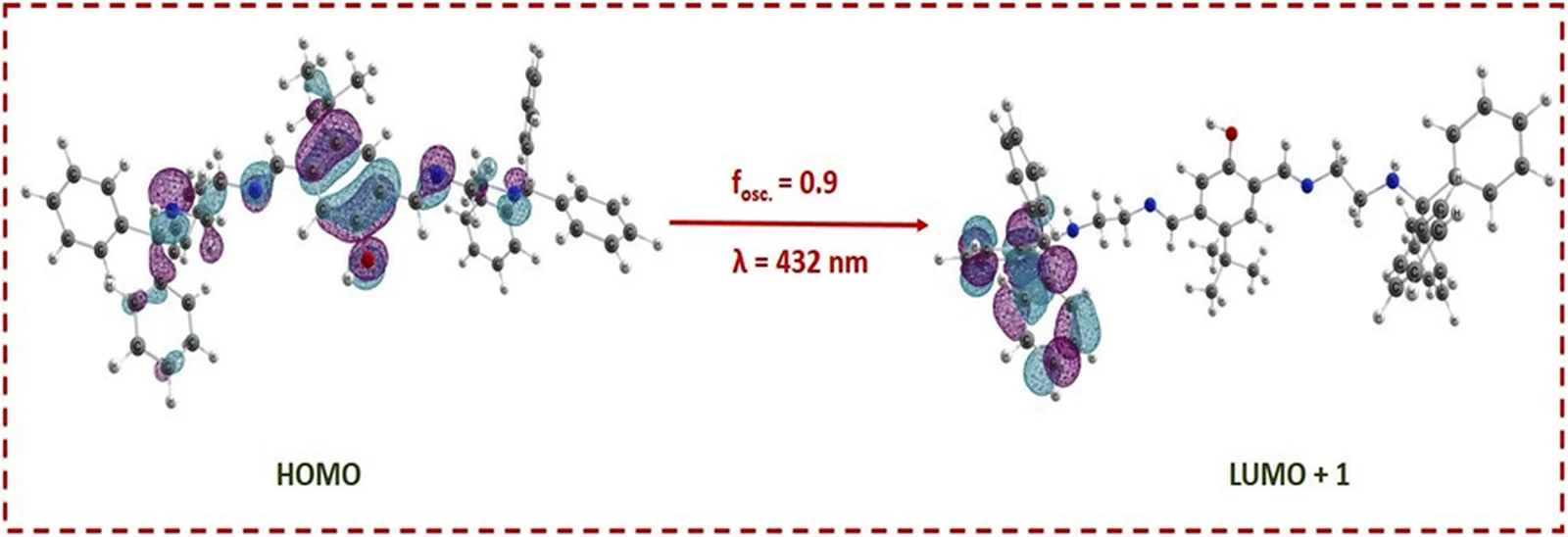
Isodensity plot representing absorption wavelengths of sensor (2) at B3LYP/def2-SVP level of theory, showing HOMO and LUMO+1 distributions.

FMO analysis reveals that the electron density of both the HOMO and LUMO are mainly centered in the middle of the molecule (*i.e.*, predominantly focused on the phenolic ring with some portions on the sides where *N*-atoms are attached). This distribution suggests the presence of strong π → π* transitions with various n → π* transitions accompanied by intramolecular charge transfer (ICT) in the molecule. The occurrence of charge transfer from one part of the molecule to another is a characteristic feature of Photoinduced Electron Transfer (PET).^101–103^ The HOMO–LUMO energy gap (Δ*E*) of 4.088 eV reflects a relatively stable electronic structure; however, this ground-state gap (≈303 nm) does not reproduce the experimentally observed absorption at 448 nm. To assign the prominent vertical excitations, time-dependent DFT (TD-DFT) calculations were performed at the same level of theory (B3LYP/def2-SVP). The calculations revealed that the HOMO → LUMO+1 transition is the prominent transition, with an oscillator strength (*f*_osc_) of 0.9 among all allowed transitions and an absorption wavelength (*λ*_abs_) of 432 nm, which closely matches the experimental value. As can be seen from the isodensity plot (Fig. 19), the HOMO is located on the central region and LUMO+1 is shifted toward the left side of the molecule (bearing three phenyl rings), suggesting significant Intramolecular Charge Transfer (ICT) character. While TD-DFT calculations successfully reproduce the absorption properties, excited-state redox potentials 
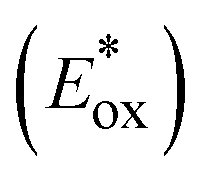
 and the one-electron reduction potential of PA were not computed in this work; the PET assignment is therefore consistent with the photophysical data and quenching kinetics but requires future theoretical validation.

### 3.19 Binding energy between the sensor and PA

**Fig. 20:**
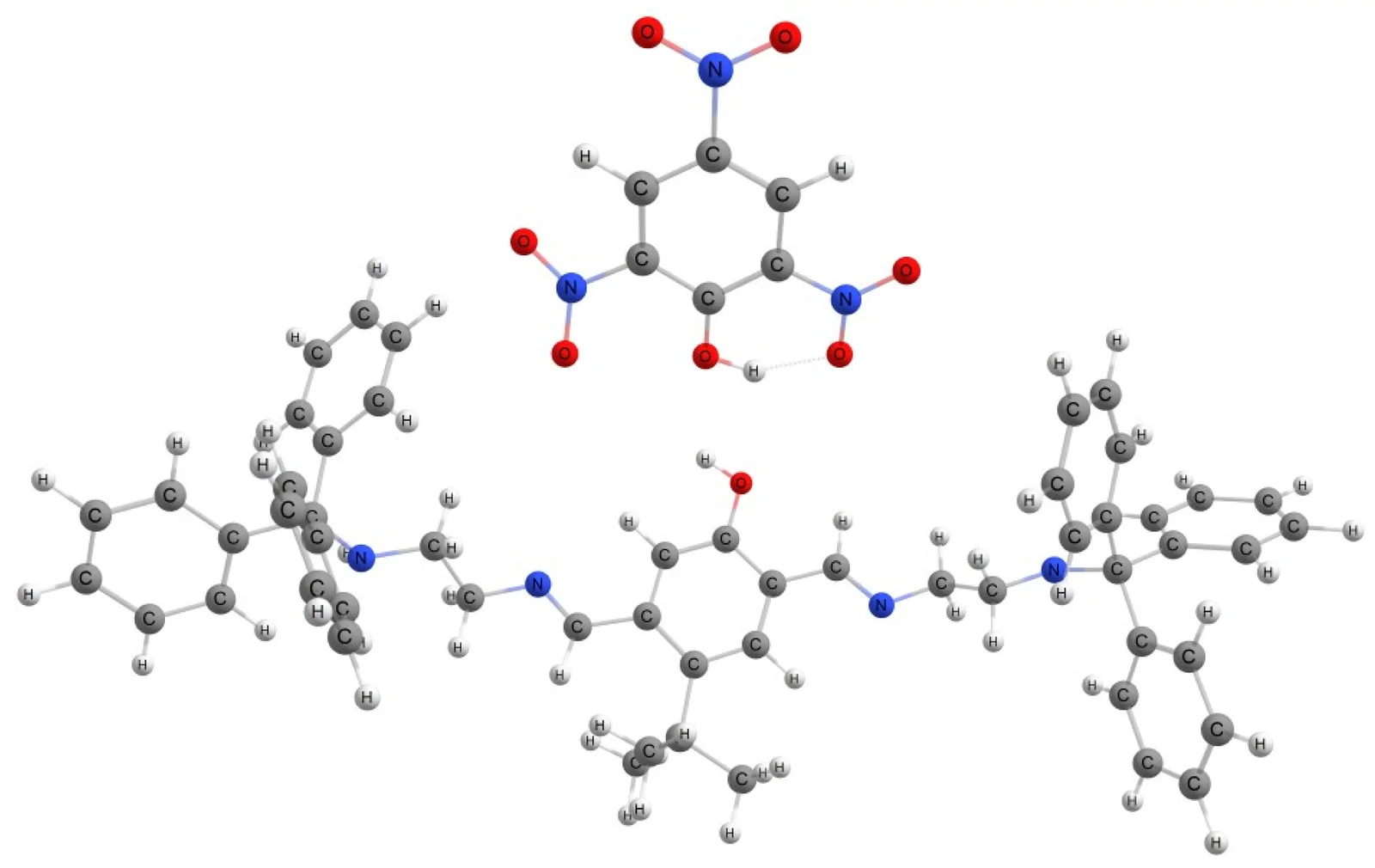
Optimized structure of sensor–PA complex at B3LYP/def2-SVP level of theory.

**Fig. 21:**
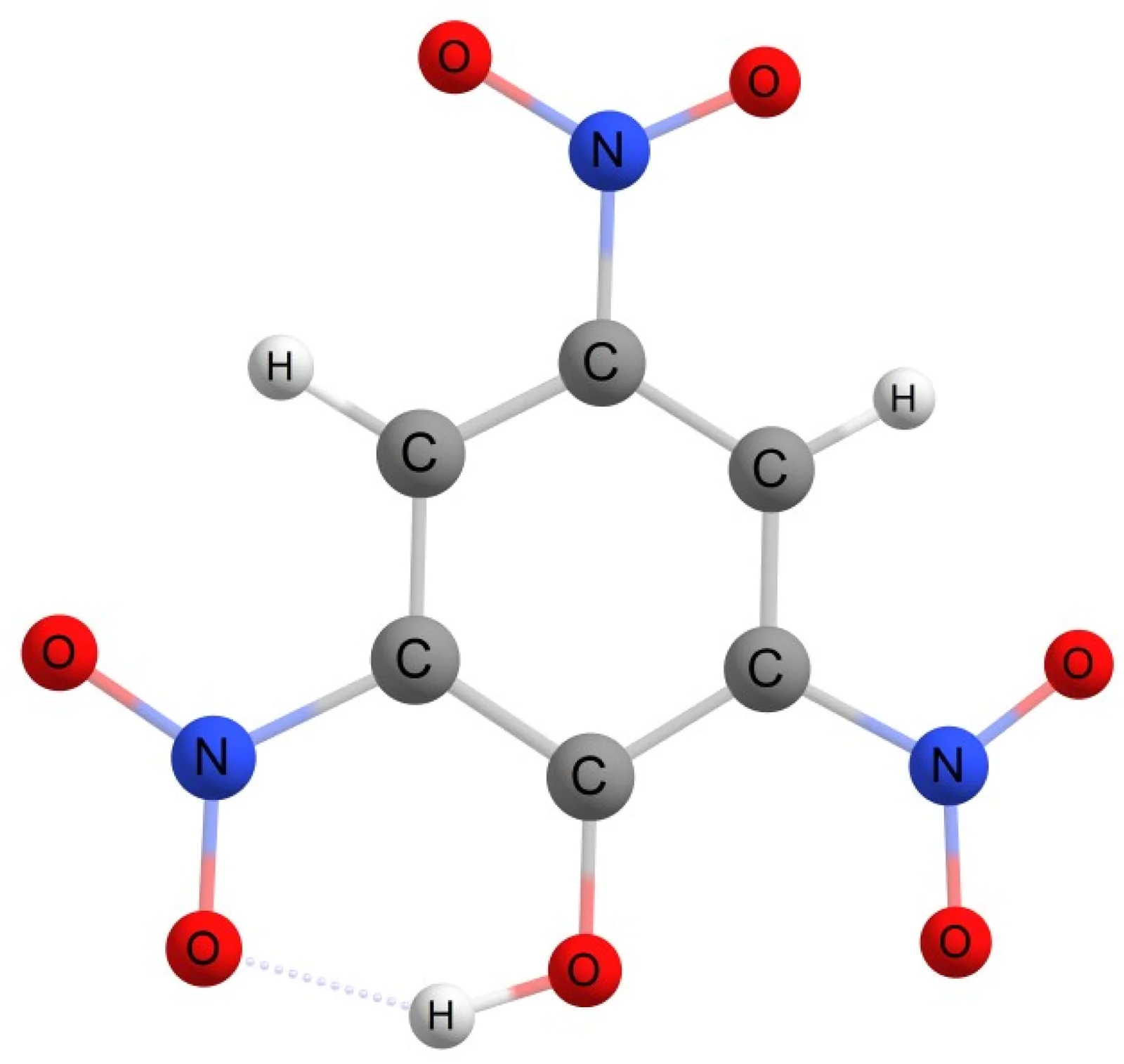
Optimized structure of PA at B3LYP/def2-SVP level of theory.

The interaction or binding energy (Δ*E*_int_) between the sensor and PA was calculated at the B3LYP/def2-SVP level of theory in methanol using the CPCM solvation model. The interaction energy was determined using the following equation:

7 Δ*E*_int_ = *E*_sensor–PA_ − (*E*_sensor_ + *E*_PA_)

where *E*_sensor–PA_, *E*_sensor_ and *E*_PA_ represent the optimized energies of sensor–PA complex, sensor and PA, respectively. The optimized structure of the sensor–PA complex is presented in Fig. 20, while its corresponding optimized Cartesian coordinates are provided in Table S7 in the SI file. The optimized structures of the isolated sensor and PA are shown in Fig. 17 and 21, respectively, with the optimized coordinates of PA provided in Table S8 in the SI file. The binding energy calculated using eqn (7) was found to be −31.51 kJ mol^−1^. The reasonably negative interaction energy indicates that the formation of the sensor–PA complex is energetically favorable, suggesting a stable binding interaction between the sensor and PA.

## 4 Conclusion

In summary, sensor (2) was successfully synthesized and demonstrated to function as a highly sensitive and selective fluorescent probe for the detection of picric acid (PA) in solution. The fluorescence response of sensor (2) was systematically evaluated in the presence of a range of nitroaromatic and non-aromatic analytes, including nitromethane (NM), nitrobenzene (NB), picric acid (PA), 2-nitrotoluene (2-NT), 3-nitrotoluene (3-NT), 4-nitrotoluene (4-NT), and 2,4-dinitrotoluene (2,4-DNT), among which PA induced the most pronounced quenching effect. The efficient sensing performance is attributed to the formation of a protonated sensor–picrate ion pair, facilitating strong intermolecular interactions. In methanolic media, the fluorescence of sensor (2) was quenched through a combination of static (ground-state ion-pair/charge-transfer complexation) and dynamic (collisional, PET-driven) processes. This assignment is supported by the upward-curving Stern–Volmer plot, a bimolecular quenching constant well above the diffusion limit, the measurable Benesi–Hildebrand association constant, and the reduction in fluorescence lifetime upon PA addition.

The positive deviation observed in the Stern–Volmer plots is consistent with this combined static-and-dynamic (sphere-of-action) mechanism and agrees with the lifetime measurements. Quantitative analysis yielded a Stern–Volmer quenching constant (*K*_SV_) of 6.309 × 10^4^ M^−1^ and a low detection limit of 0.756 µM for PA. Beyond solution-phase sensing, sensor (2) was successfully implemented for rapid, visual, on-site detection of PA using a portable filter paper platform, underscoring its practical applicability. Moreover, sensor (2) exhibits pronounced aggregation induced enhanced emission (AIEE) behavior, further enhancing its utility in solid-state sensing applications. Collectively, these findings establish sensor (2) as a robust and versatile AIEE-active fluorescent probe with significant potential for real-world explosive detection.

## Author contributions

Amarsinh R. Mainak: methodology, formal analysis, investigation, validation, visualization, software, data curation, writing – original draft, writing – review & editing. Prabhakar A. Chandanwale: methodology, investigation, validation, formal analysis, writing – review & editing, investigation, data curation. Fatemah M. Al-Douseri: investigation, validation, software, writing – review & editing, resources. Aabid Hamid: methodology, formal analysis, software, writing – original draft, writing – review & editing, investigation, data curation. Pranjali S. Kewate: formal analysis, data curation, software, writing – original draft, investigation. Radhakrishnan M. Tigote: methodology, investigation, validation, formal analysis. Noorullah Baig: conceptualization, supervision, validation, data curation, writing – review & editing, visualization. Sheik Saleem Pasha: supervision, methodology, validation, investigation, data curation, writing – review & editing, visualization. Ali Shuaib: supervision, conceptualization, methodology, validation, investigation, data curation, writing – review & editing, visualization, project administration, funding acquisition. Sidram R. Pujari: supervision, conceptualization, resources, formal analysis, methodology, data curation, writing – original draft, writing – review and editing, visualization, investigation and validation.

## Conflicts of interest

The authors declare no conflicts of interest.

## Supplementary Material

RA-OLF-D6RA05626A-s001

## Data Availability

Supplementary information (SI): experimental procedures, the ^1^H and ^13^C-NMR, FTIR, ESI-MS, UV-vis and fluorescence data, and copies of spectra. See DOI: https://doi.org/10.1039/d6ra05626a.
