# Supplementary material for: An aggregation induced enhanced emission-active salicylaldehyde Schiff base: a selective fluorescent sensor for picric acid
Source: RSC Adv. 2026 Sep 4;16(43):52905–25. doi: 10.1039/d6ra05626a (PMC13542892; doi:10.1039/d6ra05626a)
Supplement: Supplementary file 1 [file RA-016-D6RA05626A-s001.pdf]

Supplementary Information (SI) for RSC Advances.
This journal is © The Royal Society of Chemistry 2026

Supporting Information

An aggregation induced enhanced emission-active salicylaldehyde Schiff base: a selective fluorescent sensor for picric acid

Amarsinh R. Mainak[1], Prabhakar A. Chandanwale[2], Fatemah M. Al-Dousari[3], Aabid Hamid[4], Pranjali S. Kewate[1], Radhakrishnan M. Tigote[5], Noorullah Baig*[3], Sheik Saleem Pasha*[6], Ali Shuaib*[7], and Sidram R. Pujari*[1]

[1]Department of Chemistry, D. B. F. Dayanand College of Arts and Science, Solapur, Maharashtra, 413002, India.

[2]Department of Chemistry, Sangola Mahavidyalaya, Sangola, Maharashtra, 413002, India.

[3]Department of Physics, College of Science, Kuwait University, Sabah Al Salem University City, P.O. Box 5969, Safat 13060, Shadadiya, Kuwait.

[4]Department of Chemistry, University of Kashmir, Hazratbal – 190006, Jammu & Kashmir, India.

[5]Department of Chemistry, Dr. Babasaheb Ambedkar Marathwada University, Sub Campus, Dharashiv, Maharashtra, 413501, India.

[6]CSIR-Central Salt and Marine Chemicals Research Institute, Bhavnagar, Gujarat, 364002, India.

[7]Biomedical Engineering Unit, Department of Physiology, College of Medicine, Kuwait University, P.O. Box 24923, Safat-13110, Kuwait.

*Corresponding author Email:
Sidram R. Pujari: pujari_aarush@yahoo.co.in
Ali Shuaib: ali.shuaib@ku.edu.kw
Sheik Saleem Pasha: saleemifplabc@gmail.com
Noorullah Baig: noor.at.gust@gmail.com

Contents

Synthesis of compound **(1)**	(i)
^{1}H NMR spectra of compound (**1**)	Fig. S1
^{13}C NMR spectra of compound (**1**)	Fig. S2
IR spectra of compound (**1**)	Fig. S3
^{1}H NMR spectra of compound (**2**)	Fig. S4
^{13}C NMR spectra of compound (**2**)	Fig. S5
HRMS spectra of compound (**2**)	Fig. S6
IR spectra of compound (**2**)	Fig. S7
Fluorescence titration study of sensor (**2**) (10^{-4} M) with different amounts of NM in MeOH at λ_{ex}=448 nm	Fig.S8
Fluorescence titration study of sensor (**2**) (10^{-4} M) with different amounts of NB in MeOH at λ_{ex}=448 nm	Fig. S9
Fluorescence titration study of sensor (**2**) (10^{-4} M) with different amounts of 2-NT in MeOH at λ_{ex}=448 nm	Fig. S10
Fluorescence titration study of sensor (**2**) (10^{-4} M) with different amounts of 3-NT in MeOH at λ_{ex}=448 nm	Fig. S11
Fluorescence titration study of sensor (**2**) (10^{-4} M) with different amounts of 4-NT in MeOH at λ_{ex}=448 nm	Fig.S12
Fluorescence titration study of sensor (**2**) (10^{-4} M) with different amounts of 2,4-DNT in MeOH at λ_{ex}=448 nm	Fig.S13
Emission spectra of Sensor (**2**) in different pH buffer solutions.	Fig.S14
Variation in the pH of the Sensor (**2**) solution with increasing PA concentration ($0–6.0 \times 10^{-4}$ M).	Fig.S15
pH of Sensor (**2**)–PA systems before and after addition of PA	Table S1
Fluorescence response of the proposed probe toward different concentrations of picric acid ($0–6.0 \times 10^{-4}$ M) in (a) river water and (b) tap water.	Fig.S16
Quenching efficiency (%) of Sensor **(2)-**PA systems	Table S2
Fluorescence response of Sensor (**2**) under acidic conditions.	Fig.S17

The fluorescence lifetime values of sensor (**2**) with different amounts of PA	Table S3
Job's plot data for the Sensor (**2**)–PA system.	Table S4
ESI-MS spectra of Sensor (**2**)	Fig. S18a
ESI-MS spectra of PA	Fig. S18b
ESI-MS spectra of Sensor (**2**) upon addition of PA	Fig. S18c
(a) UV–Vis absorption spectra of Sensor (**2**) upon addition of increasing concentrations of PA (0–6.0 × 10^{-4} M).	Fig. S19
Inner Filter Effect (IFE)	(iii)
Observed and inner-filter-effect (IFE)-corrected fluorescence responses of Sensor (**2**) in the presence of increasing concentrations of picric acid (PA) (0-6 x 10^{-4} M).	Fig. S20
Evaluation and correction of the inner filter effect on the fluorescence response of Sensor (**2**) toward increasing concentrations of picric acid (PA).	Table S5
Dynamic light-scattering (DLS) spectra of sensor (**2**) in MeOH/water, at specific f_w ratio.	Fig. S21
Optimized coordinates of Sensor (**2**) at B3LYP/def2-SVP level of theory	Table S6
Optimized coordinates of Sensor-PA complex at B3LYP/def2-SVP level of theory.	Table S7
Optimized coordinates of PA at B3LYP/def2-SVP level of theory.	Table S8

(i) Synthesis

*Synthesis of N[1]-tritylethane-1,2-diamine (**1**):*

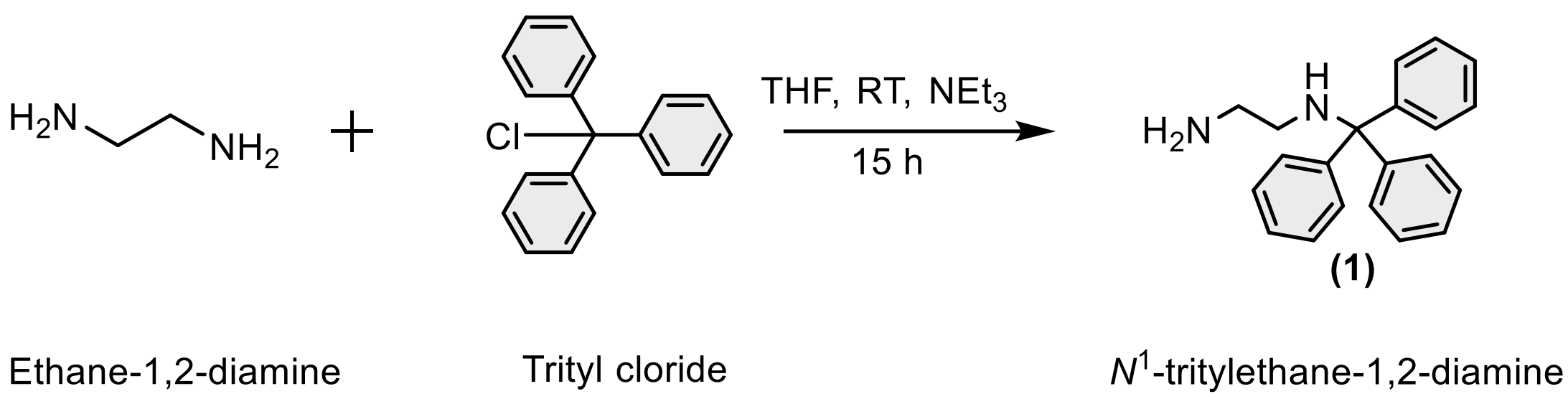

Scheme S1: Synthesis of compound (**1**)[1]

In a two-necked round-bottom flask, trityl chloride (100 mg, 0.359 mmol), triethylamine NEt_3 (0.050 mL, 0.359 mmol) and ethylenediamine (27.0 mL, 0.359 mmol) were dissolved in 10 mL THF and stirred for 15h at room temperature. After the solvent was evaporated under reduced pressure the crude product was collected and purified by column chromatography (60–120 mesh of silica gel). The yield was 70%. (Scheme S1).^{1}H NMR (400 MHz, $CDCl_3$): δ 7.46 – 7.44 (m, 6H), 7.24-7.22 (m, 6H), 7.15 (t, J = 7.2 Hz, 3H), 2.77 (t, J = 5.9 Hz, 2H), 2.18 (t, J = 6.0 Hz, 2H); ^{13}C NMR (101 MHz, $CDCl_3$): δ 146.25, 128.78, 127.93, 126.38, 70.78, 46.67, 42.93.

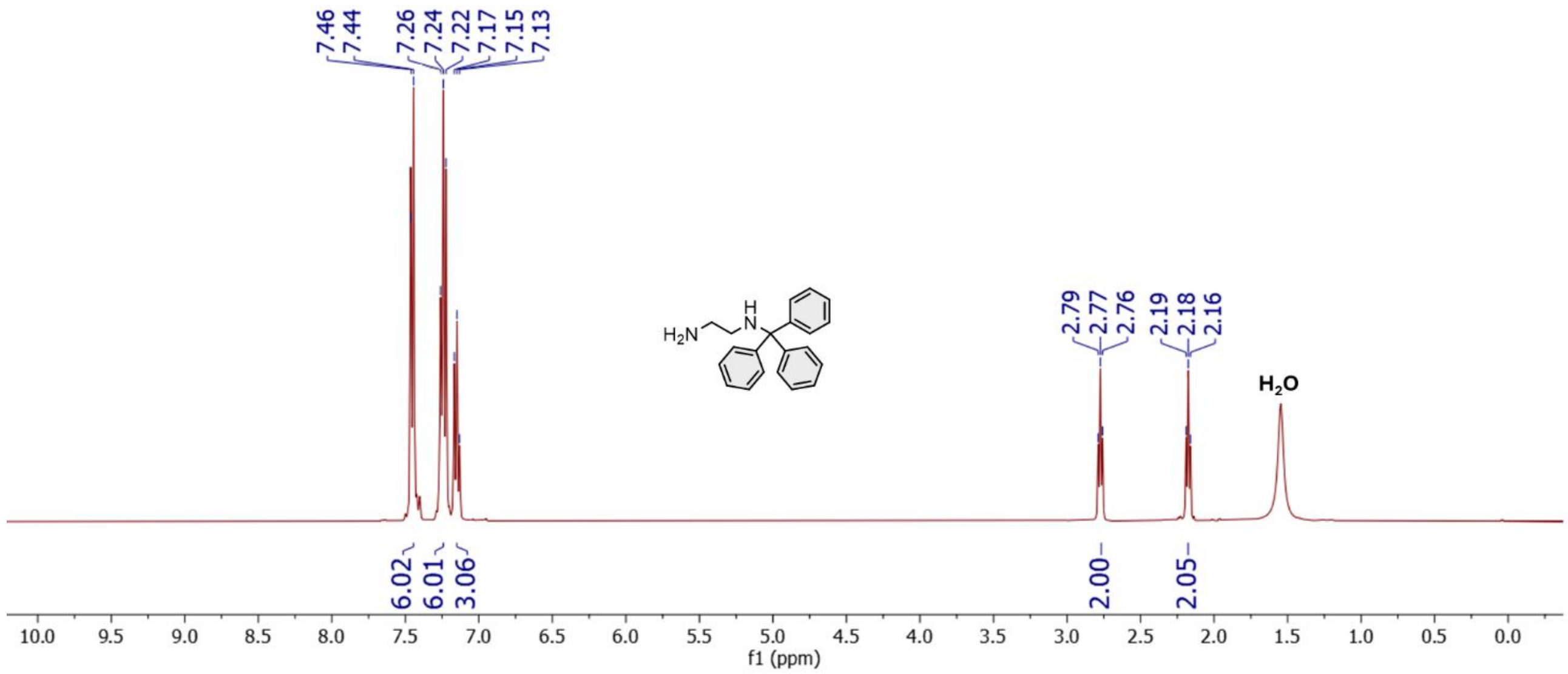

Fig. S1:^{1}H NMR spectra of compound (**1**) (400 MHz, $CDCl_3$)

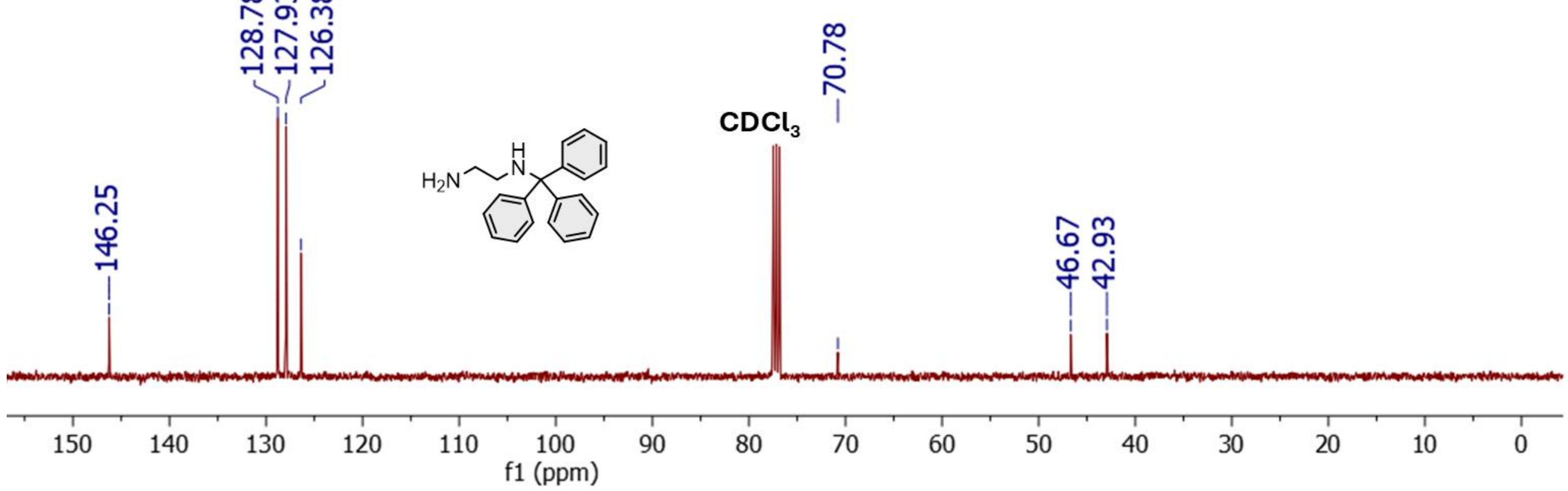

Fig. S2:^{13}C NMR spectra of compound (**1**) (101 MHz, $CDCl_3$)

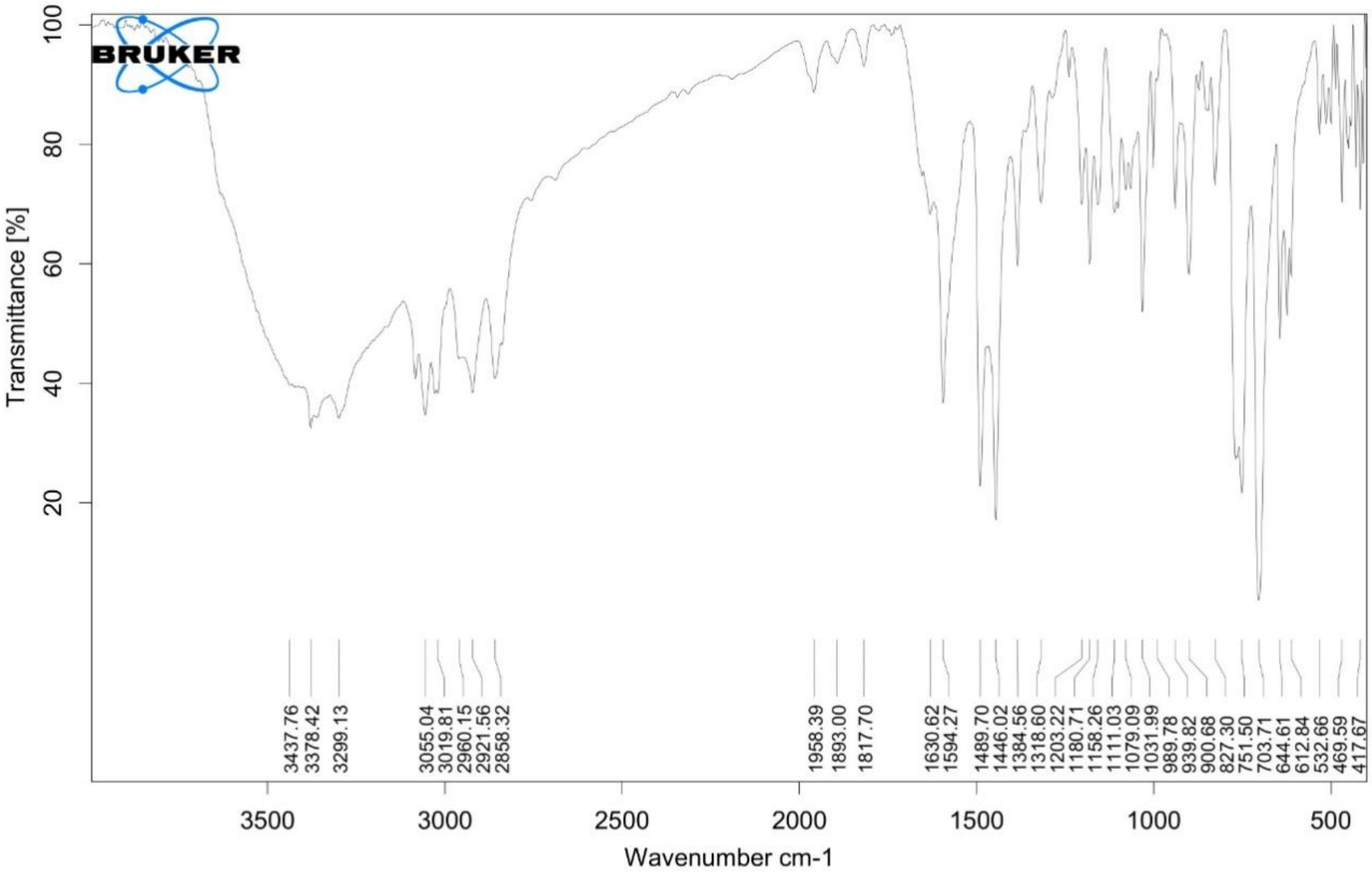

Fig. S3: IR spectra of compound (**1**)

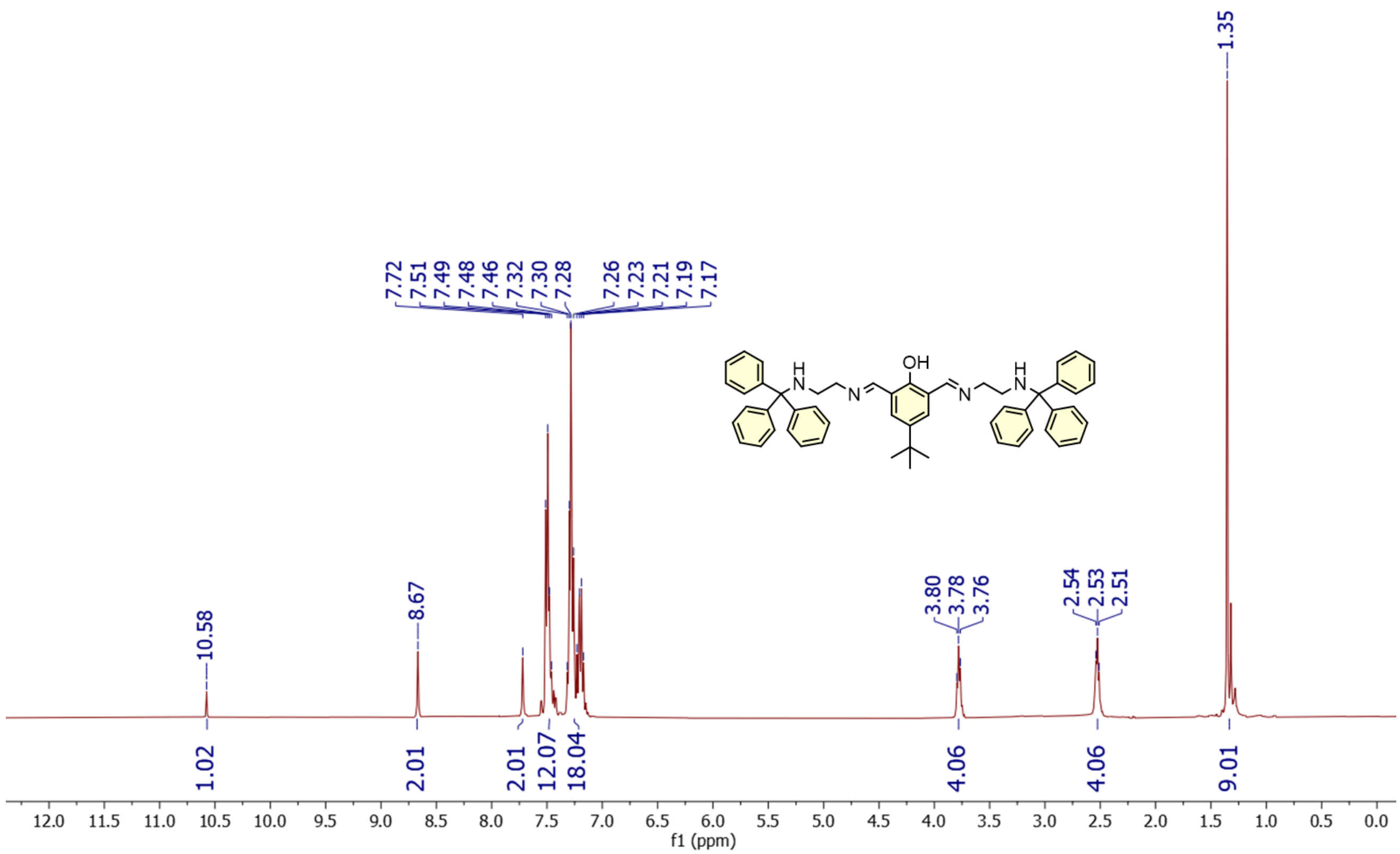

Fig. S4: ^{1}H NMR spectra of compound (**2**) (400 MHz, $CDCl_3$)

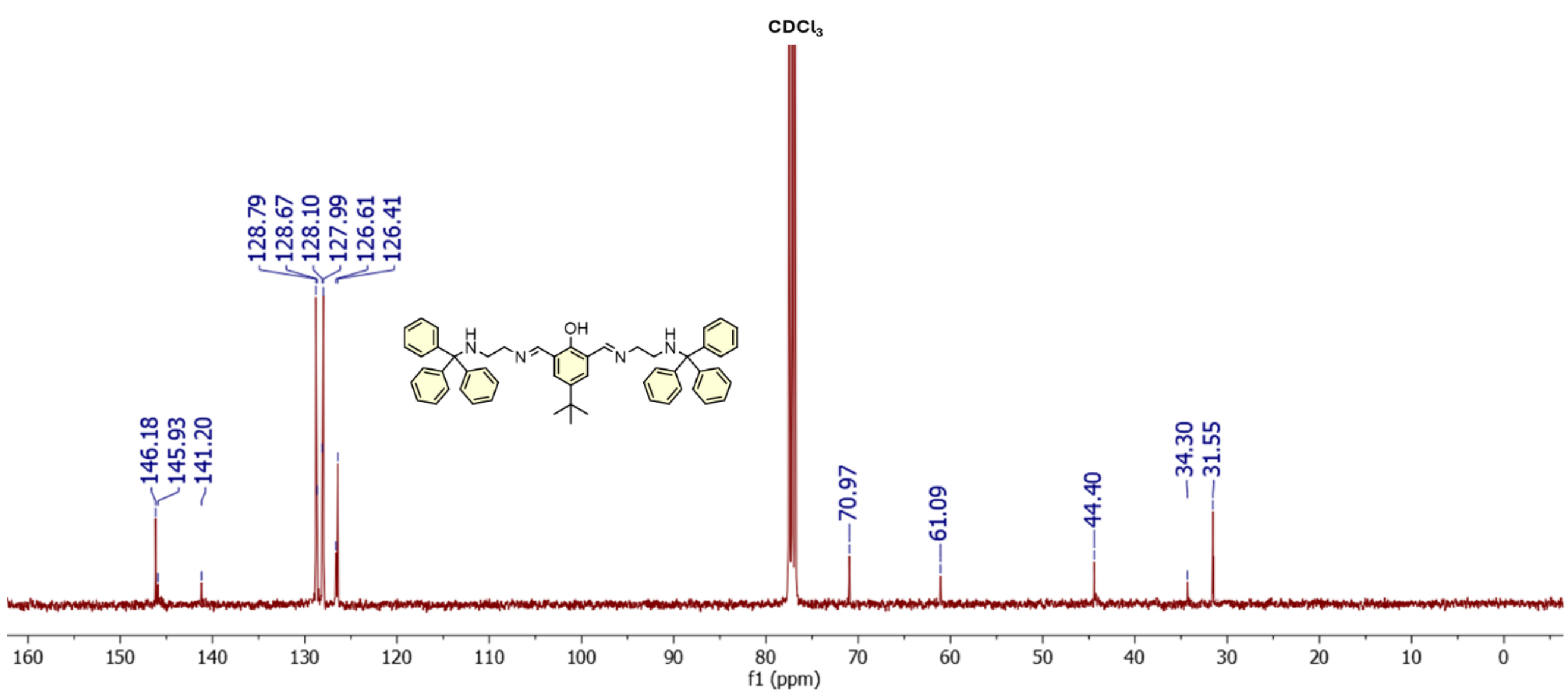

Fig. S5: ^{13}C NMR spectra of compound (**2**) (101 MHz, $CDCl_3$)

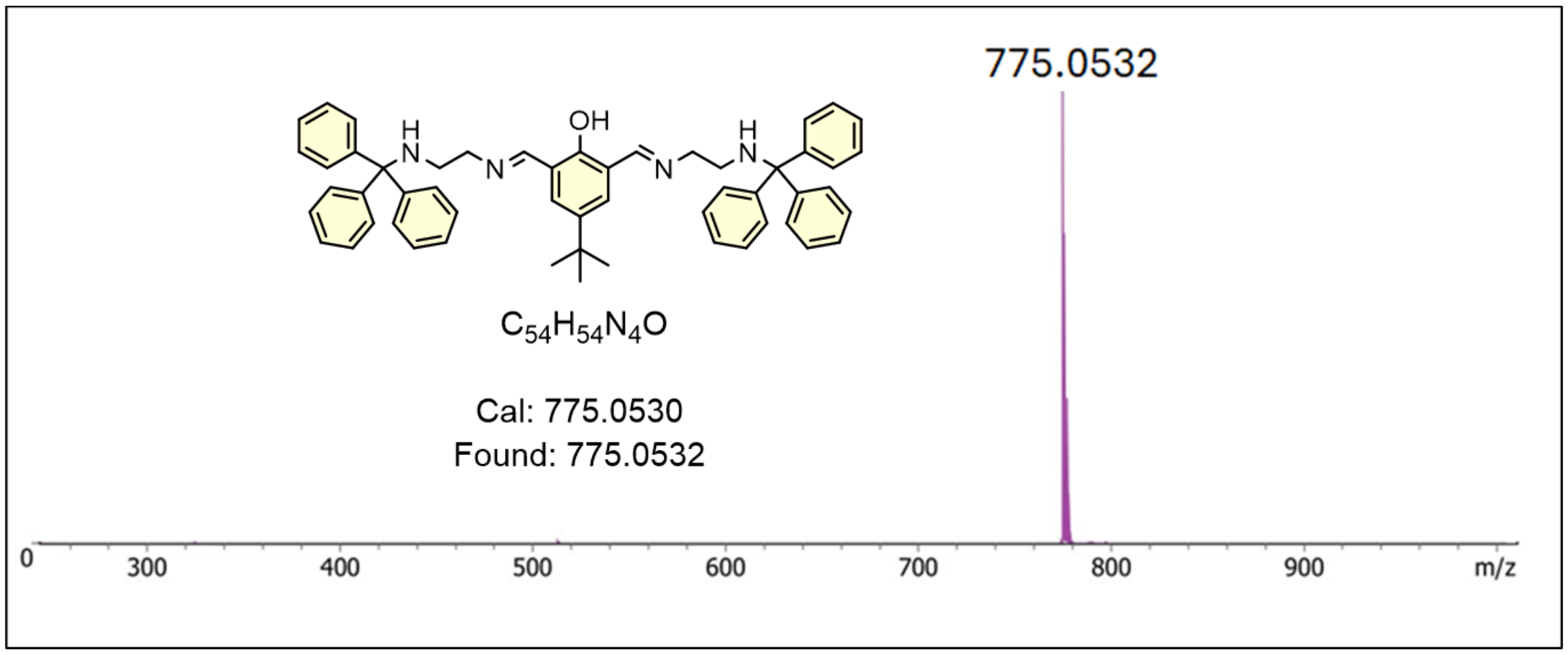

Fig. S6: HRMS spectra of compound (**2**)

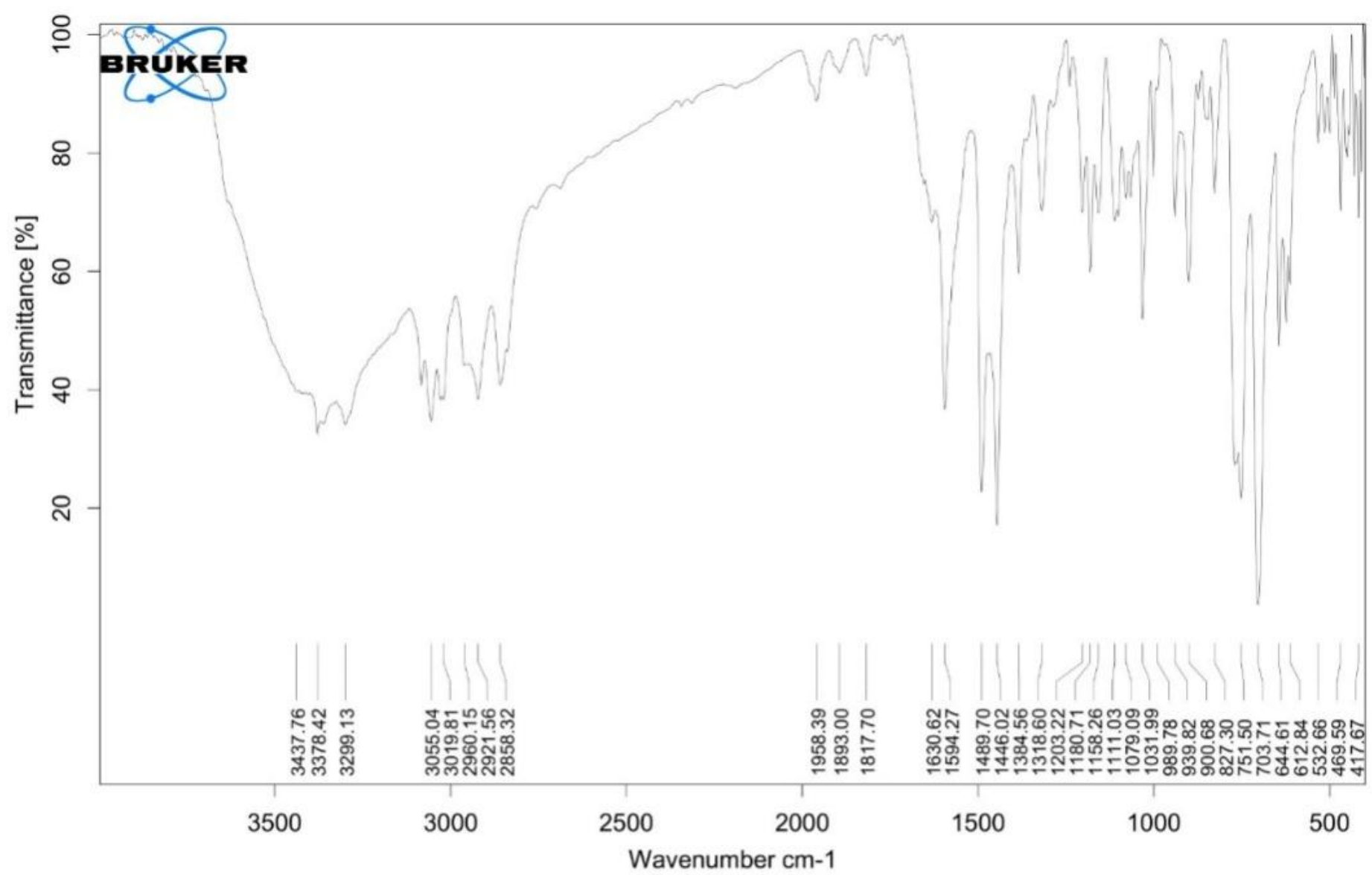

Fig. S7: IR spectra of compound (**2**)

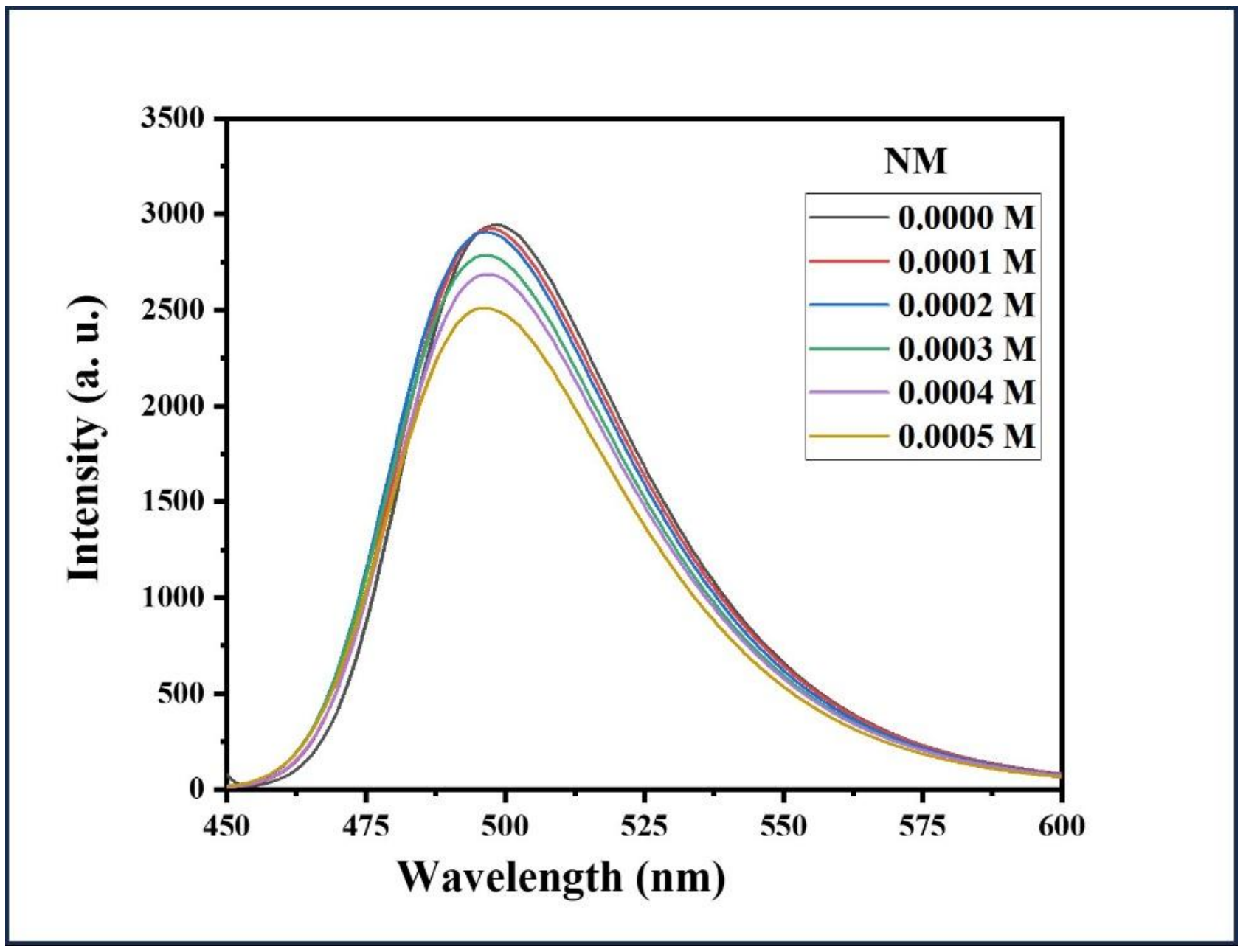

Fig.S8: Fluorescence titration study of sensor **(2)** (10^{-4} M) with different amounts of NM in MeOH at $\lambda_{ex} = 448$ nm.

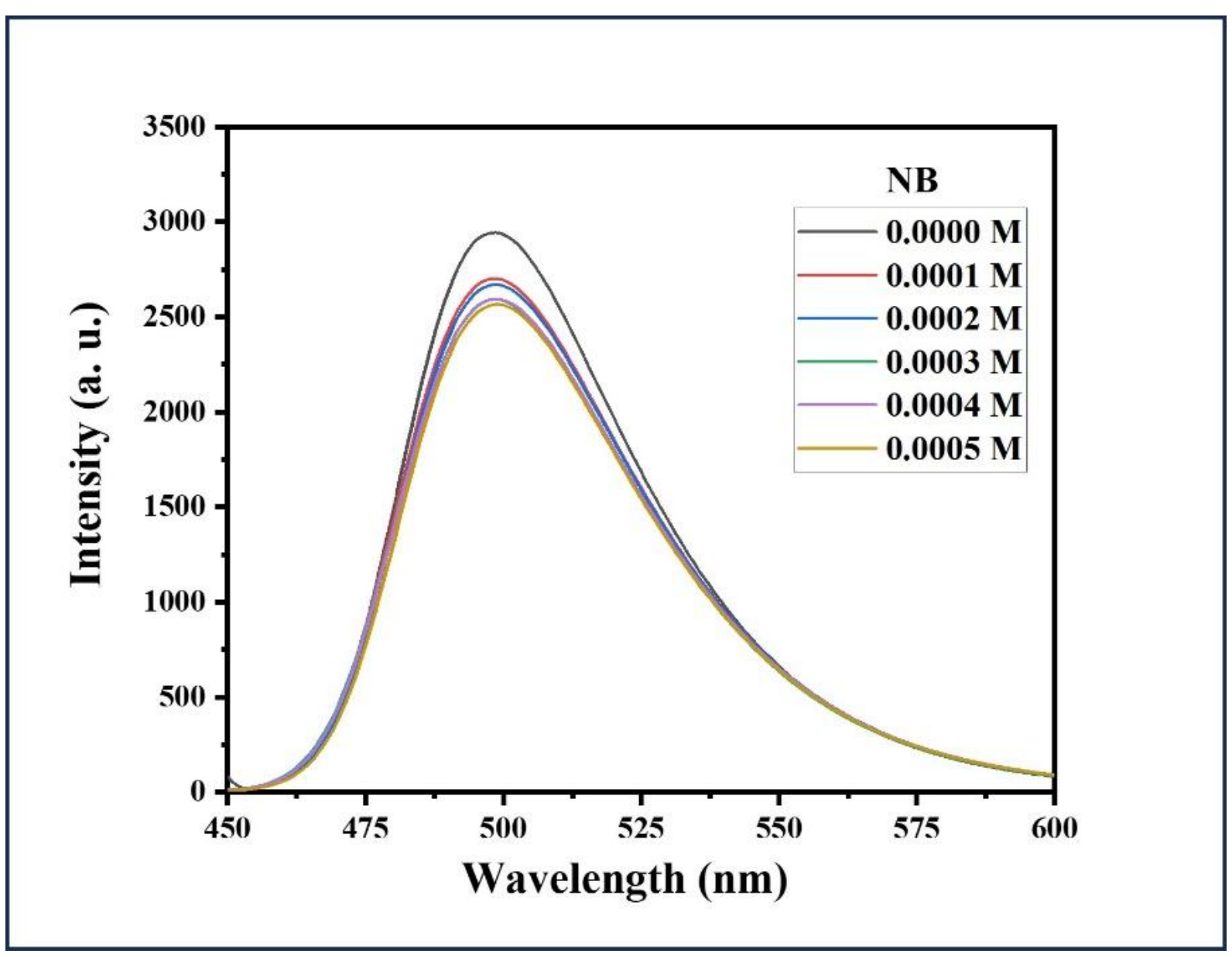

Fig.S9: Fluorescence titration study of sensor **(2)** (10^{-4} M) with different amounts of NB in MeOH at $\lambda_{ex} = 448$ nm.

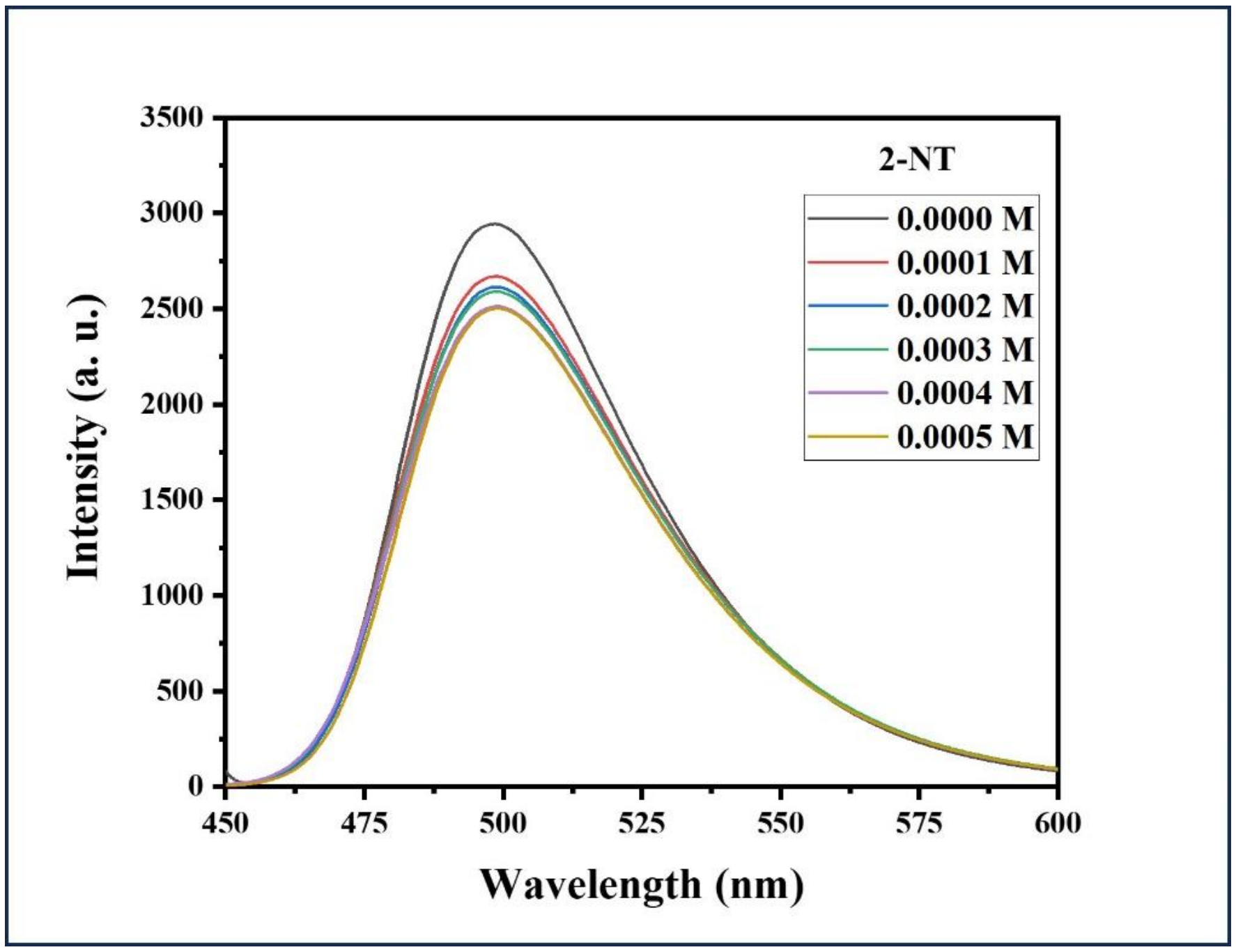

Fig.S10: Fluorescence titration study of sensor **(2)** (10^{-4} M) with different amounts of 2-NT in MeOH at λ_{ex}=448 nm.

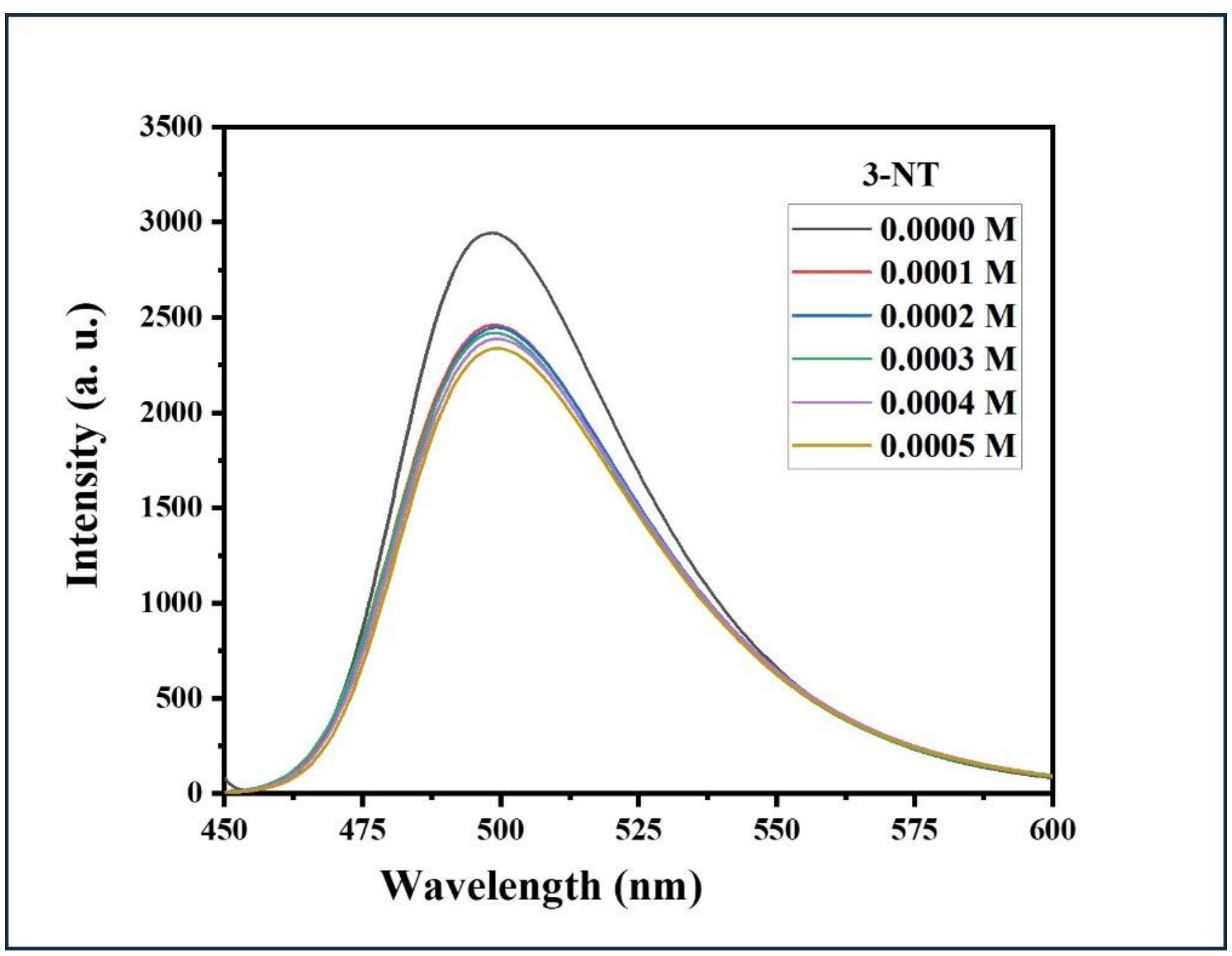

Fig.S11: Fluorescence titration study of sensor **(2)** (10^{-4} M) with different amounts of 3-NT in MeOH at λ_{ex} = 448 nm.

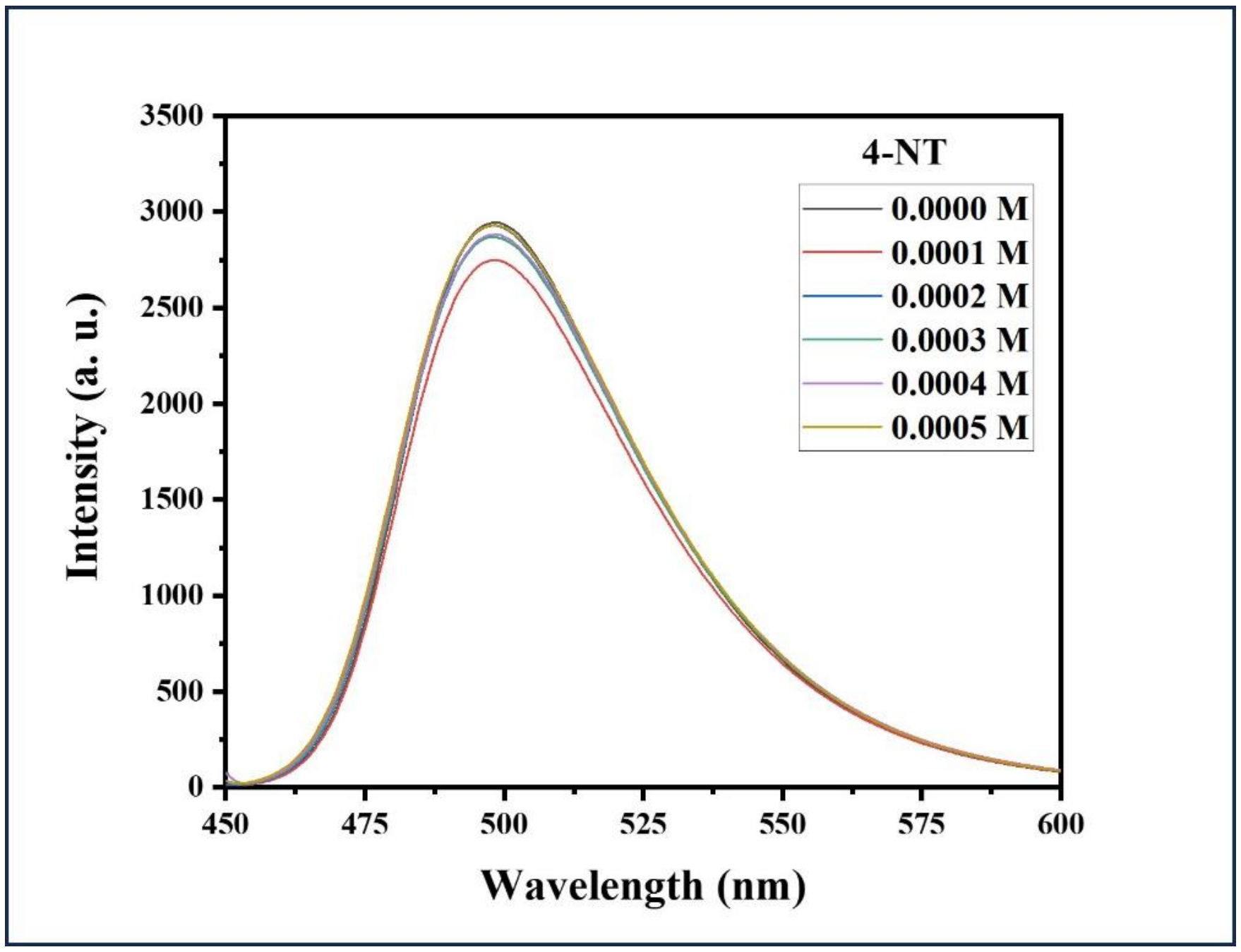

Fig.S12: Fluorescence titration study of sensor **(2)** (10^{-4} M) with different amounts of 4-NT in MeOH at λ_{ex} = 448 nm.

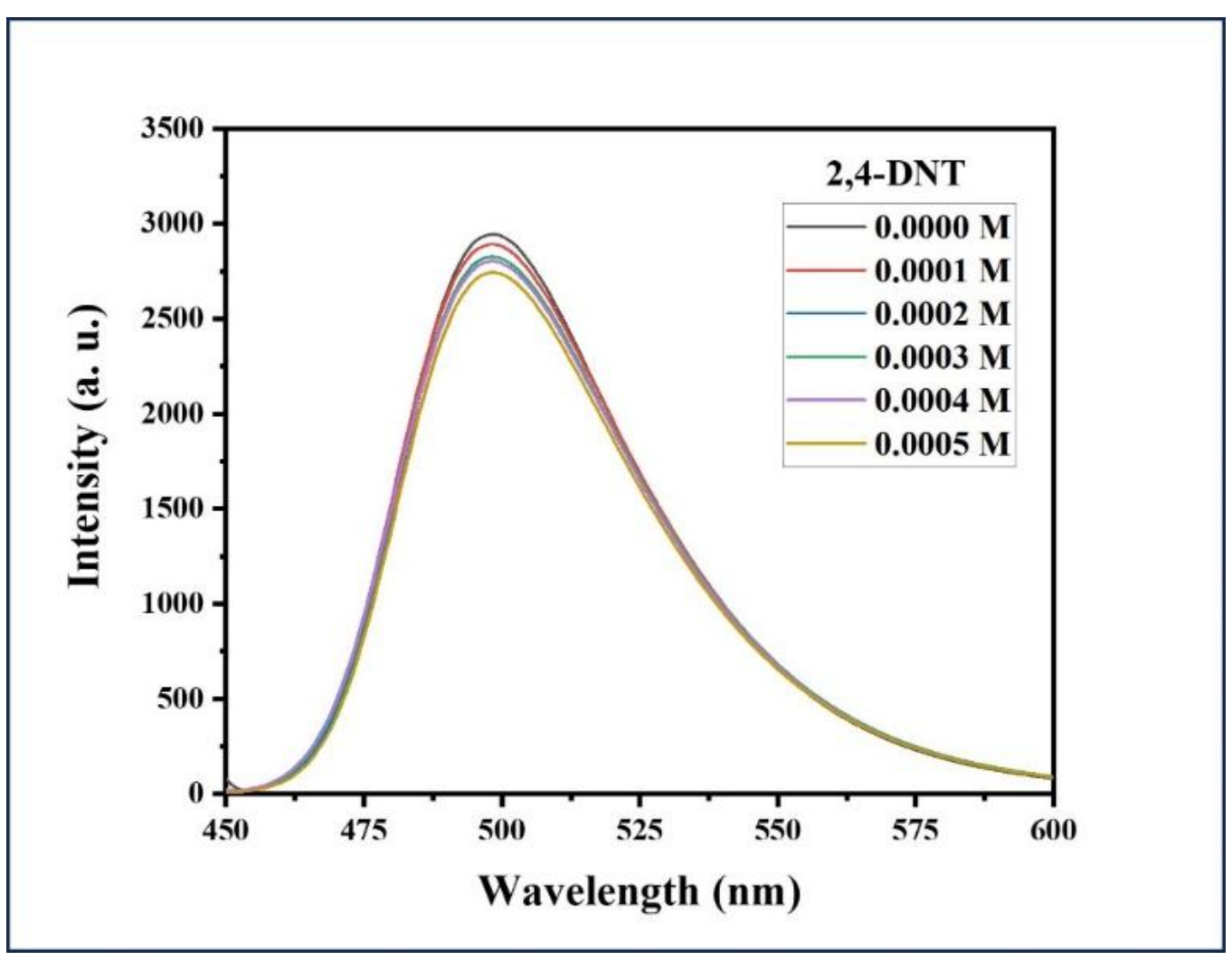

Fig.S13: Fluorescence titration study of sensor **(2)** (10^{-4} M) with different amounts of 2,4-DNT in MeOH at λ_{ex} = 448 nm.

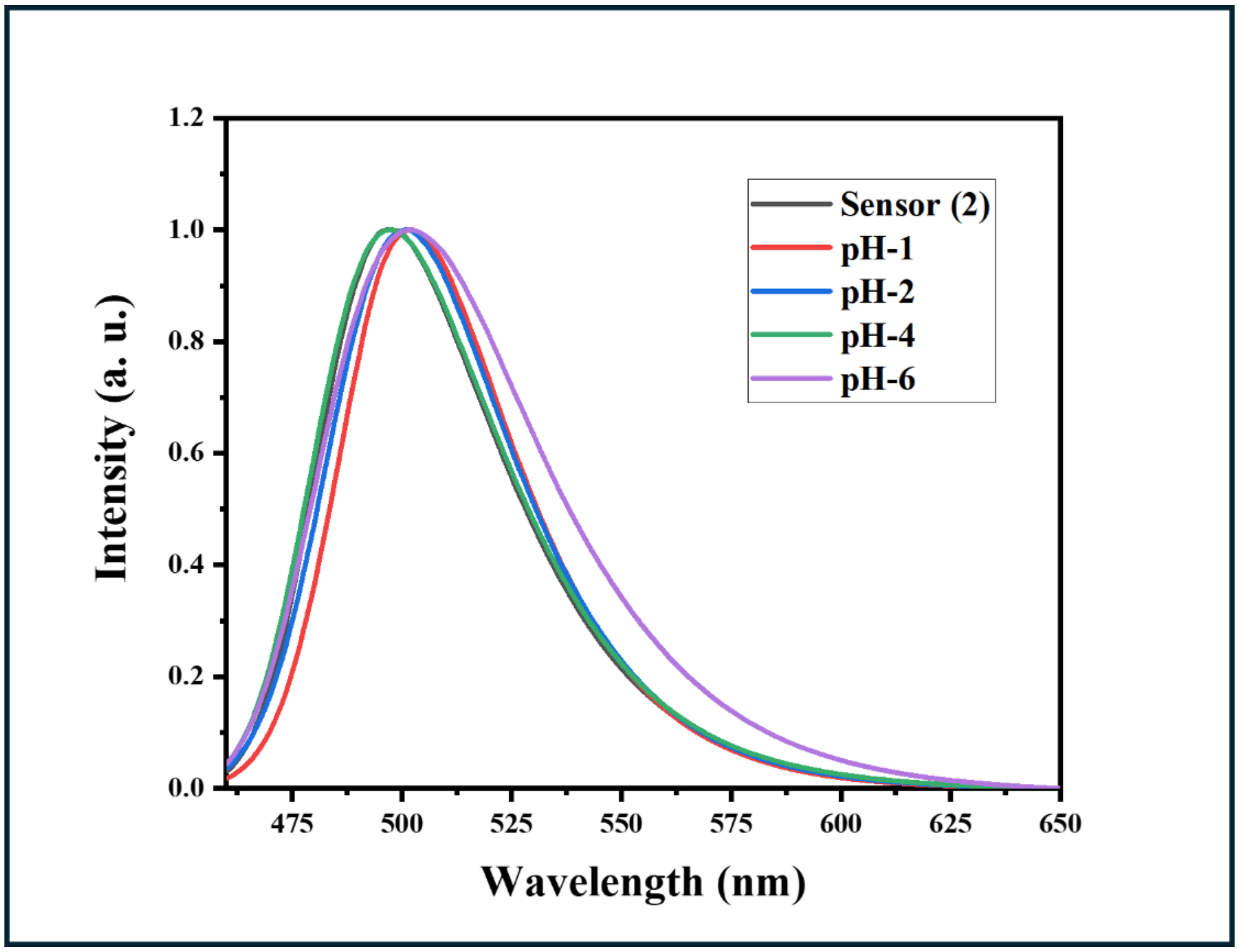

Fig. S14: Emission spectra of Sensor (**2**) in different pH buffer solutions.

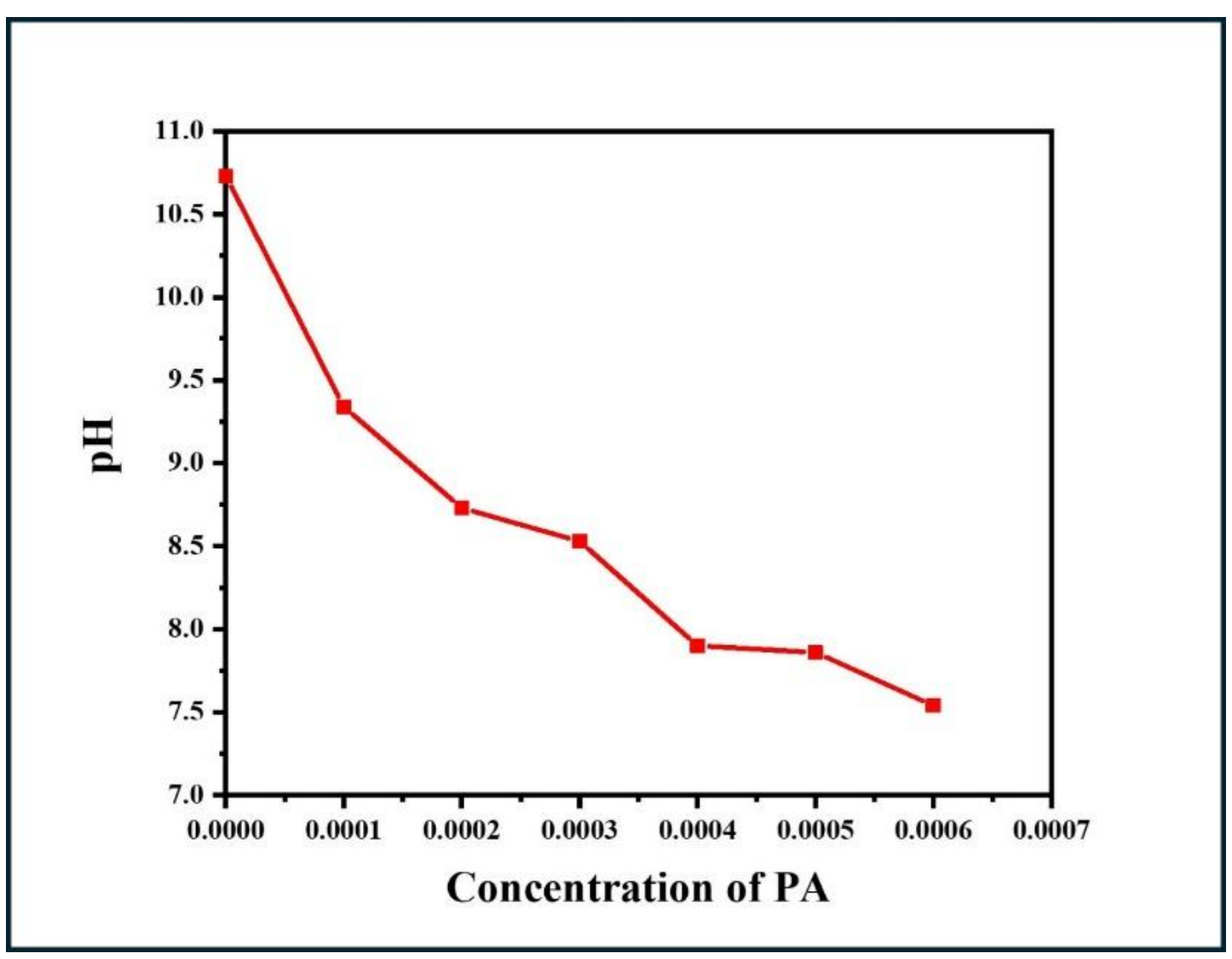

Fig. S15: Variation in the pH of the Sensor (**2**) solution with increasing PA concentration (0–6.0 × 10^{-4} M).

Table S1: pH of Sensor (**2**)–PA systems before and after addition of PA

Sr. No.	Sample	pH
1	Sensor (2)	10.73
2	0.0001 M	9.34
3	0.0002 M	8.73
4	0.0003 M	8.53
5	0.0004 M	7.90
6	0.0005 M	7.86
7	0.0006 M	7.54

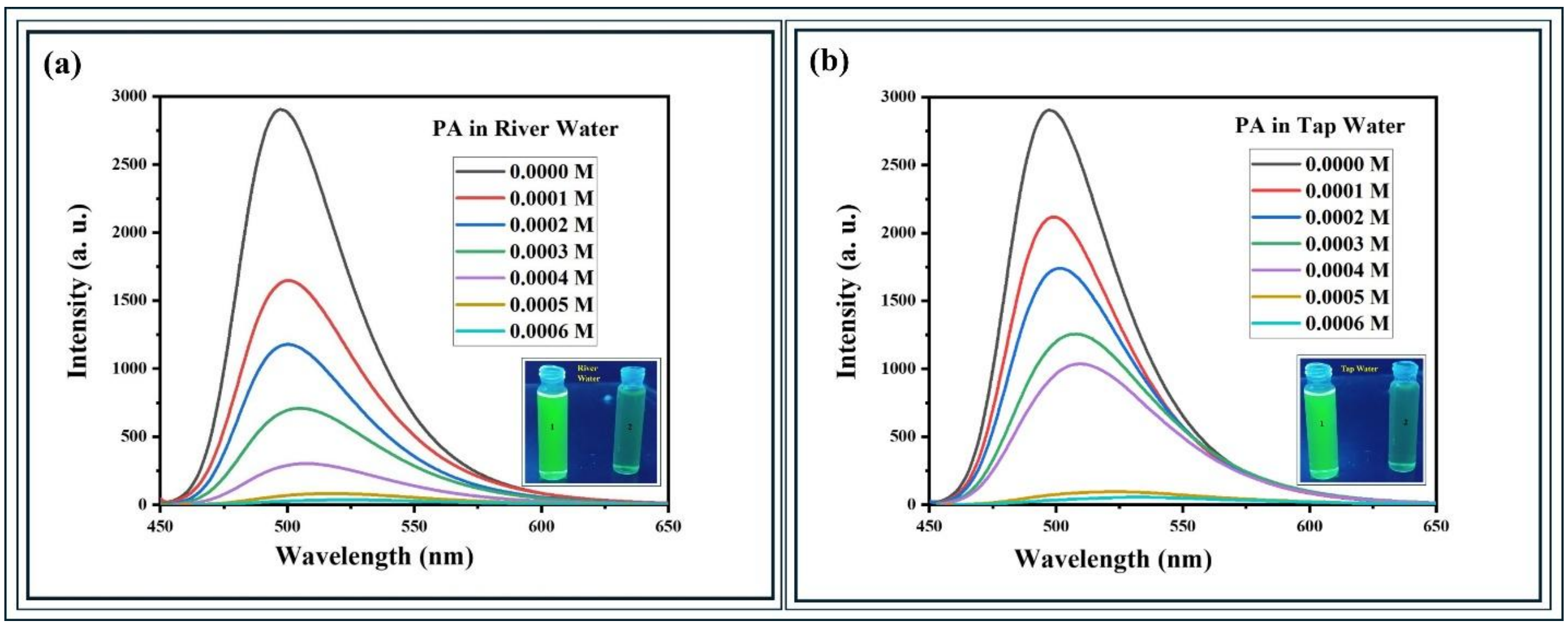

Fig.S16: Fluorescence response of the proposed probe toward different concentrations of picric acid (0–6.0 × 10^{-4} M) in (a) river water and (b) tap water. Insets show the corresponding fluorescence photographs of the probe before and after addition of PA.

Table No S2: Quenching efficiency (%) of Sensor (**2)-**PA systems

Entry	Solvent	Quenching efficiency (%)
1	Methanol	98.80
2	Tap Water	87.40
3	River Water	96.95

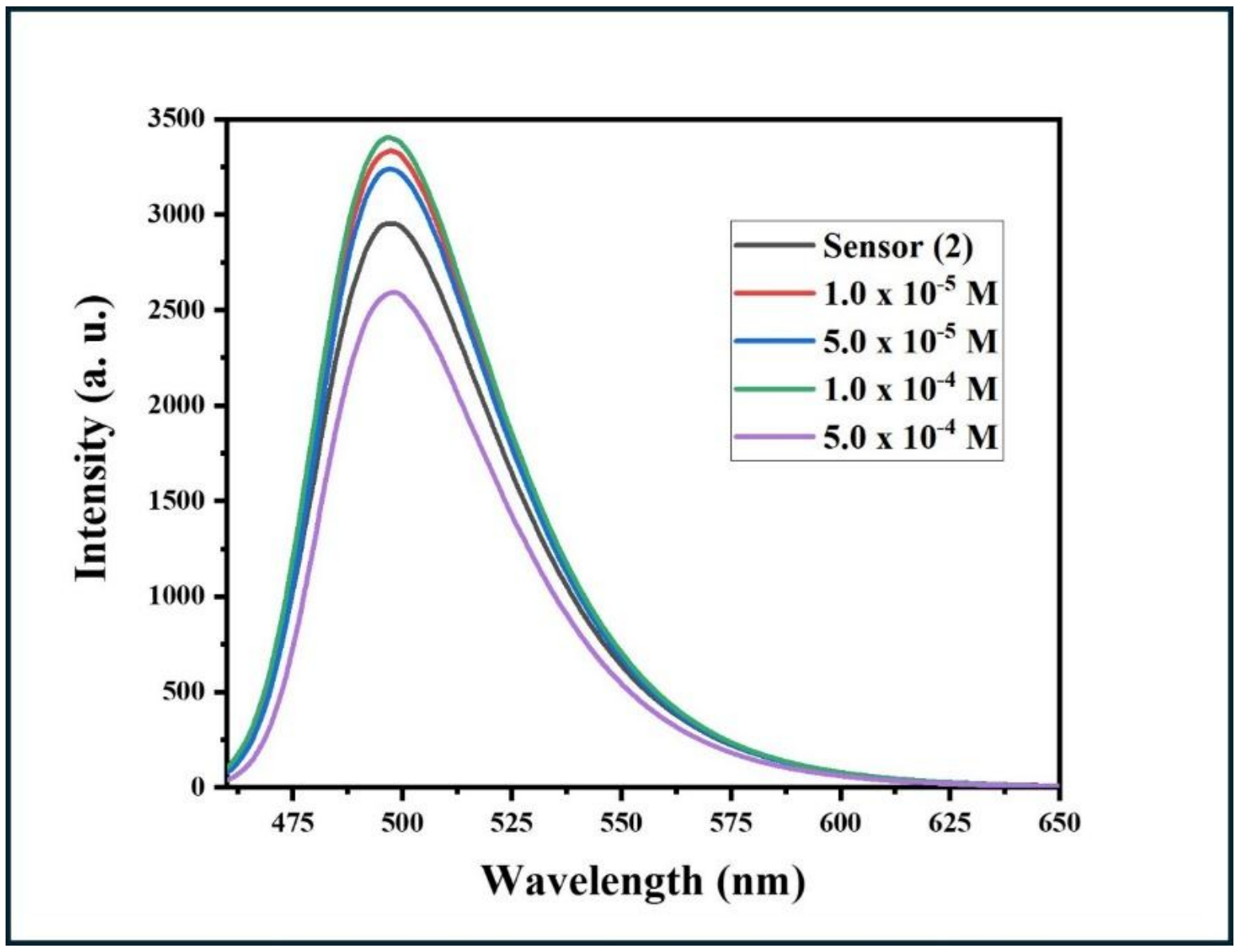

Fig.S17: Fluorescence response of Sensor (**2**) under acidic conditions. Emission spectra of Sensor (**2**) recorded in the presence of different concentrations of acetic acid (1.0×10^{-5}–5.0×10^{-4} M).

Table S3: The fluorescence lifetime values of sensor **(2)** with different amounts of PA.

**Sample**	**A_1**	**A_2**	**τ_1**	**τ_2**	**Lifetime (ns)**	**Adj. R^2 Value**
Pure (sensor **(2)**)	1033132.825	1002966.445	1.08747	1.08742	1.08744537	0.99927
Pure + 0.0001 M	2291703.234	2939.44325	1.05977	4.20208	1.063795307	0.99982
Pure + 0.0002 M	2333434.409	1409.19542	1.05317	4.65335	1.05534289	0.99988
Pure + 0.0003 M	2448704.859	66619.92623	0.90766	3.84681	0.985505198	0.99961
Pure + 0.0004 M	99880.1875	636.9582	0.44486	4.1750	0.468497194	0.99890
Pure + 0.0005 M	2.24E+10	94404.1048	0.24433	4.05137	0.244346015	0.99555
Pure + 0.0006 M	1.25E+12	35703.79516	0.19003	3.54985	0.190030096	0.98885

Table S4: Job's plot data for the Sensor (**2**)–PA system.

**Sensor (2) volume (mL)**	**PA volume (mL)**	$X_{Sen(2)}$	**Fluorescence Intensity (F)**	$\Delta F = F_0 - F$	$X_{Sen(2)}\Delta F$
5.0	0.0	1.0	1317.184	0	0
4.5	0.5	0.9	910.1461	407.0379	366.33411
4.0	1.0	0.8	783.4108	533.7732	427.01856
3.5	1.5	0.7	676.3223	640.8617	448.60319
**3.0**	**2.0**	**0.6**	**531.1057**	**786.0783**	**471.64698**
2.5	2.5	0.5	438.4344	878.7496	439.3748
2.0	3.0	0.4	352.4728	964.7112	385.88448
1.5	3.5	0.3	246.0937	1071.0903	321.32709
1.0	4.0	0.2	143.6667	1173.5173	234.70346
0.5	4.5	0.1	92.84721	1224.33679	122.433679
0.0	5.0	0.0	7.51725	1309.66675	0

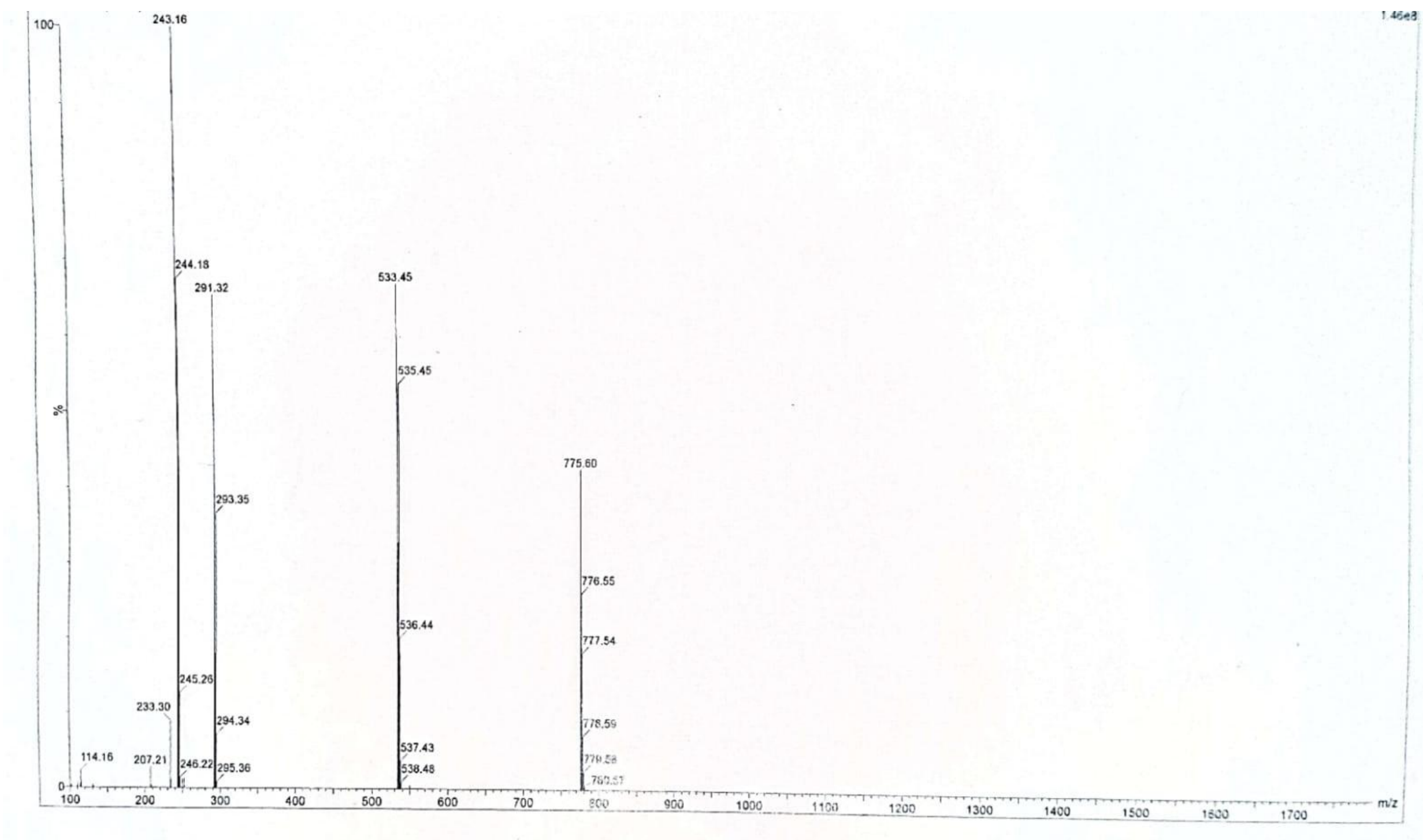

Fig. S18a: ESI-MS spectra of Sensor (**2**)

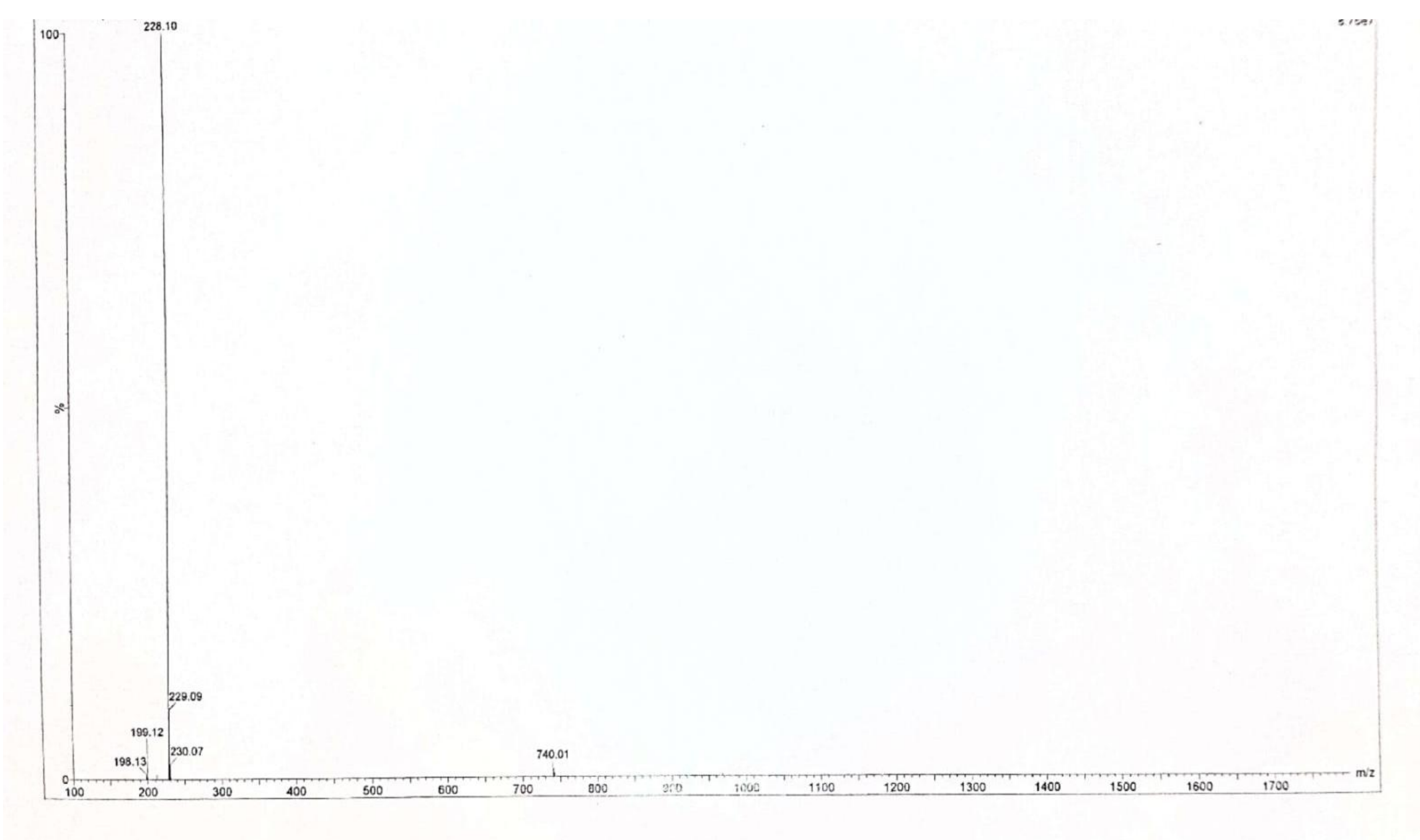

Fig. S18b: ESI-MS spectra of PA

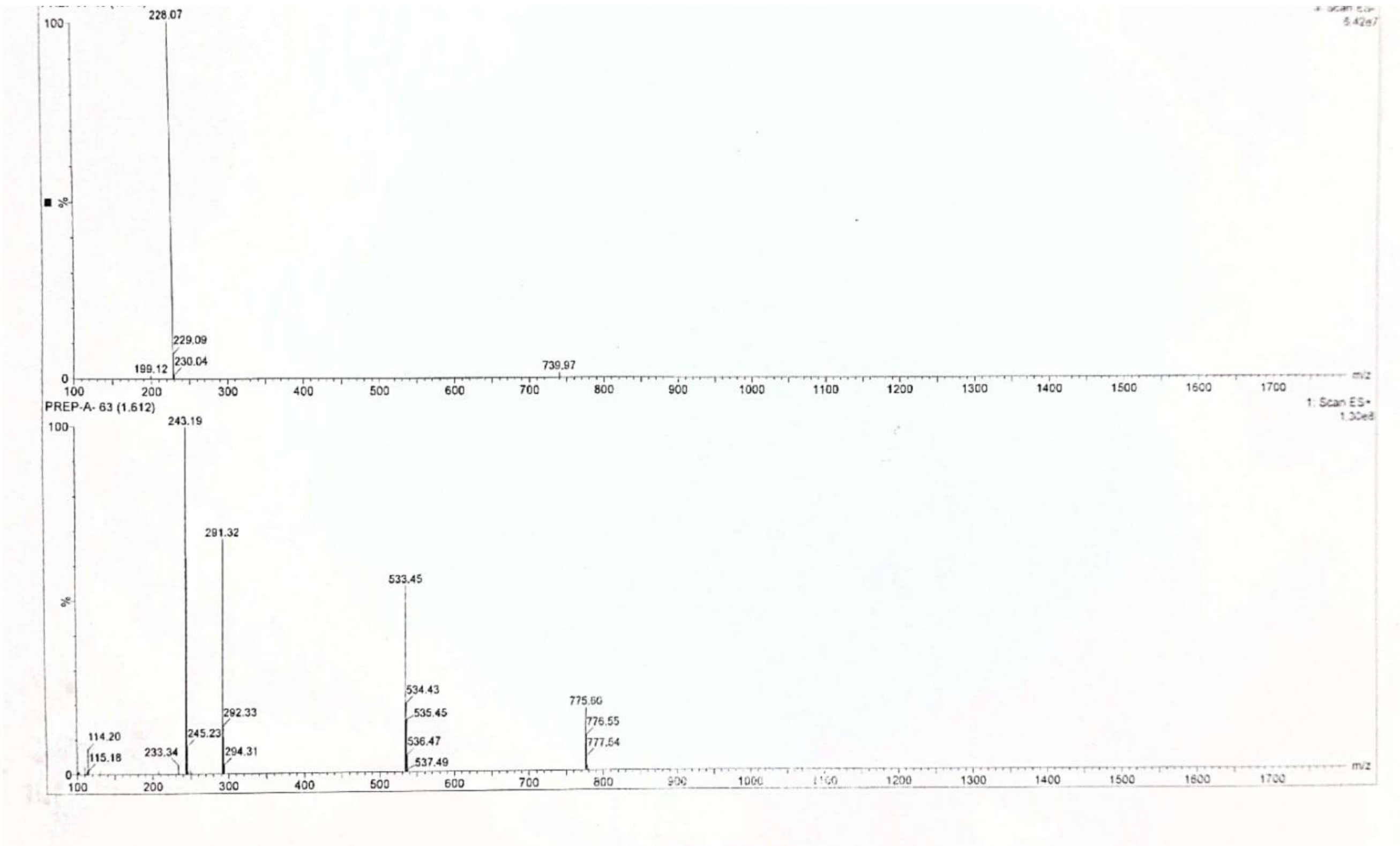

Fig. S18c: ESI-MS spectra of Sensor (**2**) upon addition of PA

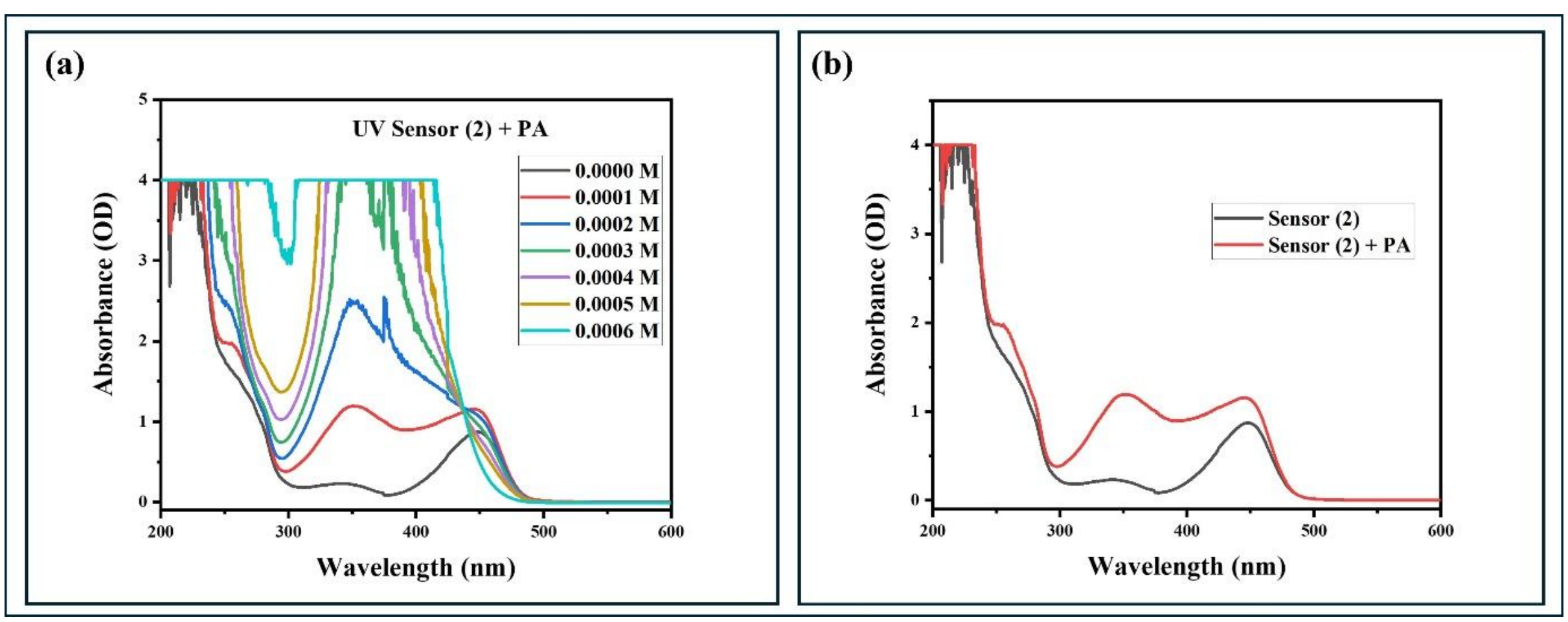

Fig. S19: (a) UV–Vis absorption spectra of Sensor (**2**) upon addition of increasing concentrations of PA (0–6.0 × 10^{-4} M). (b) Comparison of the UV–Vis absorption spectra of Sensor (**2**) before and after addition of PA.

(iii) Inner Filter Effect (IFE):

To evaluate the possible contribution of the inner filter effect (IFE) to the fluorescence quenching of Sensor (**2**) by picric acid (PA), UV–Vis absorption spectra of Sensor (**2**) in the absence and presence of increasing concentrations of PA (1×10^{-4}–6×10^{-4} M) were recorded. The absorbance values at the excitation (448 nm) and emission (498 nm) wavelengths were used to correct the measured fluorescence intensities for the IFE according to the equation:

$$F_{corr} = F_{obs} \times 10^{(A448+A498)/2}$$

where F_{obs} and F_{corr} represent the observed and IFE-corrected fluorescence intensities, respectively, while A_{448} and A_{498} are the absorbances at 448 and 498 nm, respectively. The calculated correction factors were 3.833, 3.512, 3.083, 2.624, 2.363, and 1.908 for PA concentrations of 1×10^{-4}, 2×10^{-4}, 3×10^{-4}, 4×10^{-4}, 5×10^{-4}, and 6×10^{-4} M, respectively. The comparison of the observed and IFE-corrected fluorescence intensities revealed that the IFE contributes to the observed fluorescence decrease; however, substantial quenching persists after correction, particularly at higher PA concentrations. For example, at 6×10^{-4} M PA, the observed fluorescence intensity decreased from 2750.28 to 35.14 a.u., whereas the IFE-corrected fluorescence intensity was 67.20 a.u. These results demonstrate that the observed fluorescence quenching cannot be attributed only to the inner filter effect and indicate that a genuine interaction between Sensor (**2**) and PA is responsible for a significant portion of the fluorescence response[2-6]. The IFE-correction analysis is presented in Fig. S11 and Table S1.

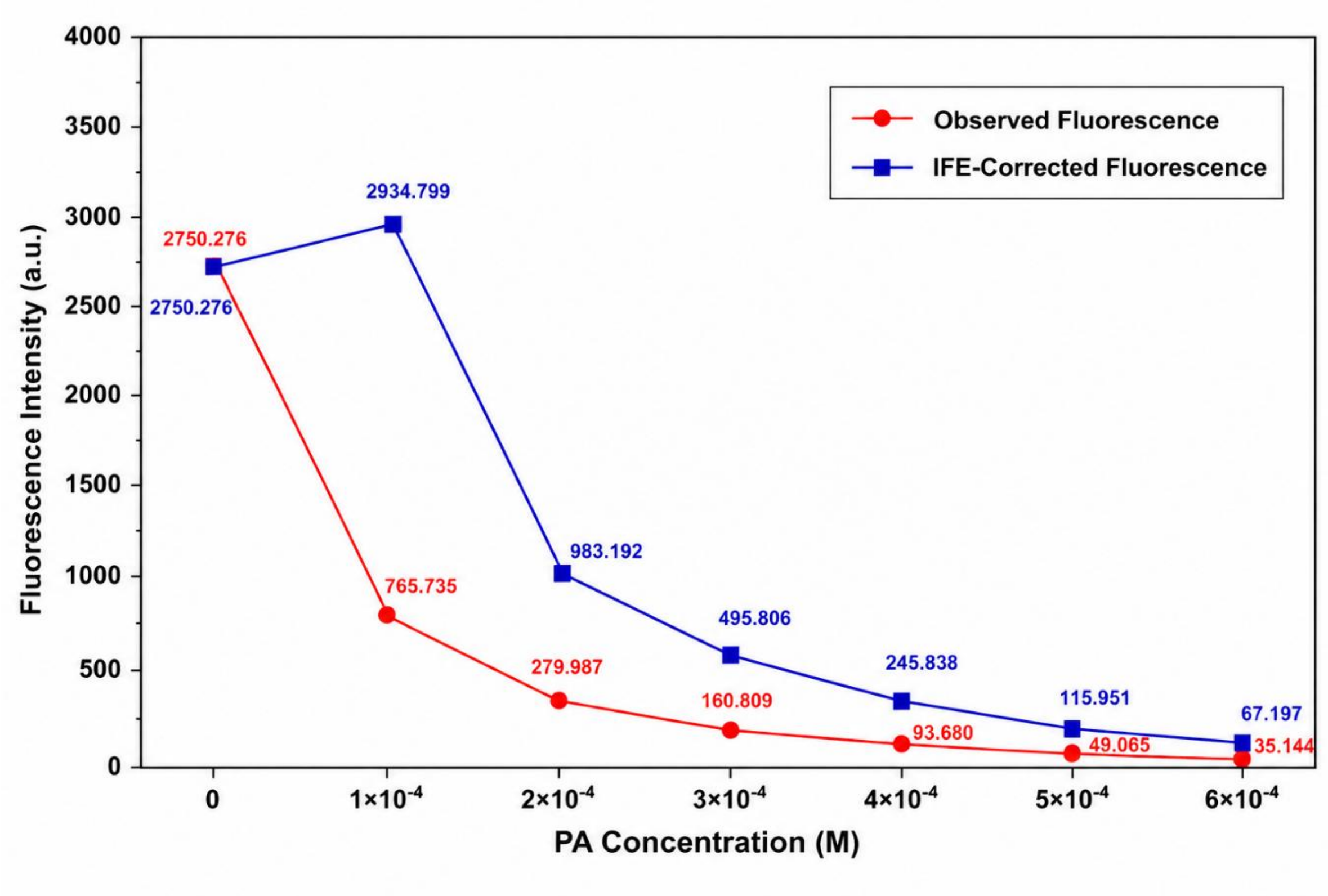

Fig. S20: Observed and inner-filter-effect (IFE)-corrected fluorescence responses of Sensor (**2**) in the presence of increasing concentrations of picric acid (PA) (0-6 x 10^{-4} M). The fluorescence intensities were corrected using the absorbance values at the excitation (448 nm) and emission (498 nm) wavelengths.

Table S5: Evaluation and correction of the inner filter effect on the fluorescence response of Sensor (**2**) toward increasing concentrations of picric acid (PA).

PA concentration (M)	A_{448}	A_{498}	F_{obs} (a.u.)	IFE correction factor $10^{(A448+A498)/2}$	F_{corr} (a.u.)
0 M	-	-	2750.28	-	2750.28
0.0001 M	1.149	0.018	765.73	3.833	2934.80
0.0002 M	1.084	0.007	279.99	3.512	983.19
0.0003 M	0.972	0.006	160.81	3.083	495.81

0.0004 M	0.832	0.006	93.68	2.624	245.84
0.0005 M	0.743	0.004	49.07	2.363	115.95
0.0006 M	0.568	<0.01	35.14	1.912	67.20

A_{448} and A_{498} represent the absorbance values at the excitation (448 nm) and emission (498 nm) wavelengths, respectively; F_{obs} and F_{corr} represent the observed and IFE-corrected fluorescence intensities, respectively.

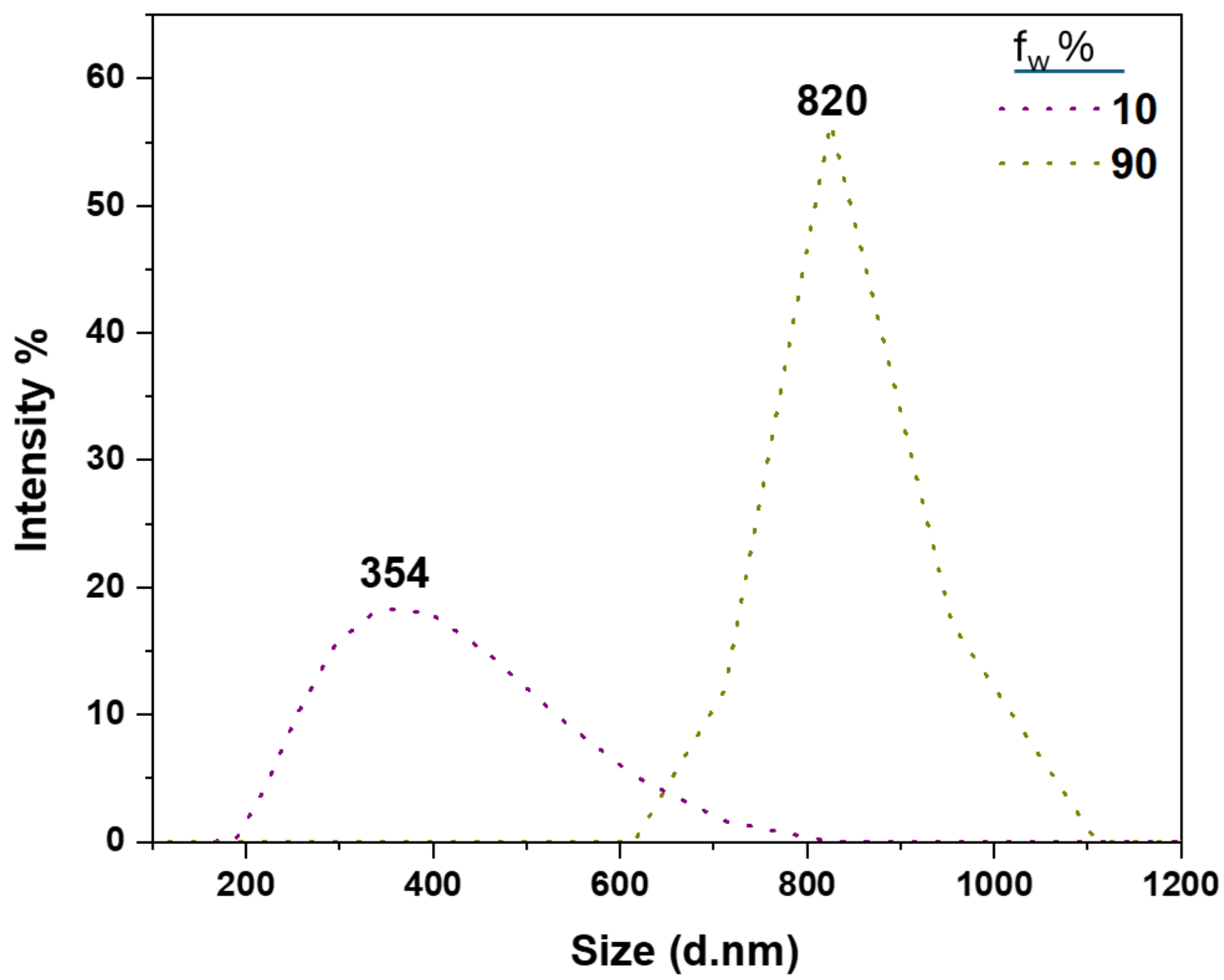

Fig. S21: Dynamic light-scattering (DLS) spectra of sensor (**2**) in MeOH/water, at specific f_w ratio.

Table S6: Optimized coordinates of Sensor (**2**) at B3LYP/def2-SVP level of theory.

C -10.26660511793252 -4.91596606835588 -6.27062486993376

C	-10.90903843024897	-4.57881674668469	-7.47716054930416
H	-11.96944669266645	-4.31656625319310	-7.47145601043709
C	-10.21812696016525	-4.57234263006012	-8.69250673331242
H	-10.74478584997277	-4.29869577799476	-9.61123181386846
C	-8.86110823074297	-4.91258311329896	-8.73463193076880
H	-8.31719822916795	-4.90388288099137	-9.68321859780494
C	-8.21256511977126	-5.27100188446966	-7.54963078853417
H	-7.15574807062374	-5.55238887662533	-7.56494507692928
C	-8.90985301809157	-5.27782232238161	-6.33503307152228
H	-8.38068304750480	-5.57307620240959	-5.42736861620627
C	-11.09747286254111	-5.06841462392582	-4.96842319943217
H	-10.13835213447998	-5.96588071430995	-0.42972696068678
C	-9.88795885245779	-5.37158390113938	-1.31320580080173
C	-10.56429466299834	-5.60580055981379	-2.51593661387937
H	-11.34329436383196	-6.36832745976909	-2.56057615530819
C	-10.26623350190952	-4.86703655403601	-3.67317995126207
C	-9.27990607244496	-3.86883755653667	-3.57987502572412
H	-9.02840167671525	-3.26359470265525	-4.45261975444243
C	-8.60412516599975	-3.62999710848537	-2.37868768916831
H	-7.83980539329902	-2.84875968809009	-2.33740555766492
C	-8.90179775937774	-4.38320531626952	-1.23774588848232
H	-8.37206567069080	-4.19881953760902	-0.29907468931562
C	-12.22630622592846	-4.00513290816489	-4.88148380498143
C	-13.50641345724072	-4.33688193307612	-4.40905961943378
C	-11.96947368549299	-2.65867403863007	-5.19970315615878
C	-14.50076321478233	-3.35952118300326	-4.27080089779283
C	-14.23322040894182	-2.02722569304681	-4.59468858643702
C	-12.95828639306536	-1.68156733769616	-5.05942628361055
H	-12.73053206670451	-0.64343012915225	-5.31752754725081
H	-10.98555168517069	-2.36324954282159	-5.56955111121291
H	-13.72837264754092	-5.36985230272717	-4.13935867720550

H	-15.49008353862037	-3.64789118489793	-3.90398555880996
H	-15.00834854212103	-1.26357333270218	-4.48639675699846
N	-11.72697208947726	-6.40518873988082	-4.96018462469739
C	-10.86968971444292	-7.57392738396114	-5.11653818232530
H	-12.45287129511152	-6.42445847030925	-5.67670006452902
C	-11.67218060190110	-8.85132049782779	-4.83797184104913
H	-10.03652841750232	-7.51166912303715	-4.39871968746947
H	-10.41217142947836	-7.65721418269970	-6.12210867284655
N	-10.87384512675145	-10.02968554857023	-5.10275801805090
H	-12.54133408627152	-8.86818006409431	-5.52354955747372
H	-12.07091297924274	-8.82701119805646	-3.80383948177165
C	-10.74414442838727	-10.90004338131833	-4.17831860474705
C	-9.93821676747143	-12.13851133405549	-4.35367283371616
C	-9.86508445271018	-13.19826863254455	-3.39289122876530
C	-9.05421785852091	-14.28753657104916	-3.73964097288491
H	-8.95138829040541	-15.13311527399875	-3.06332312636970
C	-8.32651659858849	-14.39092136694618	-4.93737431424362
C	-8.41567347505477	-13.33125565042219	-5.86547045709665
C	-9.21742849243091	-12.23354871202492	-5.55711262113322
H	-9.29713290970428	-11.40840163740549	-6.26858662059867
H	-11.23819627401234	-10.74403860364904	-3.20896950193179
O	-7.71971564827467	-13.41633681301329	-7.02493234443859
H	-7.86989257471434	-12.62218663359704	-7.56099295001855
C	-10.61425813544441	-13.20899177857488	-2.03224892208962
C	-12.14774759290410	-13.13614423131569	-2.24609648258786
H	-12.47827637628342	-12.22122858148734	-2.75580075003410
H	-12.66316990121107	-13.17602674708489	-1.27236286299941
H	-12.49281534924548	-13.99443812870286	-2.84544946150862
C	-10.35257557667733	-14.51330902122769	-1.24592109772902
H	-10.90907973902289	-14.47641461754922	-0.29627923503202
H	-9.28773141592910	-14.64514194230241	-0.99921329216562

H	-10.69286904079610	-15.40501039934095	-1.79481568349640
C	-10.11950817667436	-12.05294507714881	-1.12535270382195
H	-10.29537728133584	-11.05482784290870	-1.54855620864602
H	-9.03736565611936	-12.14848540483458	-0.93926287070835
H	-10.63419500995914	-12.09383028142730	-0.15116989736765
C	-7.48875135896428	-15.56749543517044	-5.22721205897767
N	-7.39302235975394	-16.56753647298674	-4.43863266621653
H	-6.93861166093509	-15.53946992323115	-6.18557863932256
C	-6.54350926971872	-17.67361984205680	-4.82640321285897
C	-5.55229334273211	-18.01734223298143	-3.70801110353577
H	-5.99076482015036	-17.48095747058011	-5.76830569181588
H	-7.18952581922079	-18.55635116792870	-5.00100146394113
N	-4.73540268316918	-19.15416113270866	-4.10784677319659
H	-6.12454369469737	-18.18216797753477	-2.77177284788887
H	-4.90723946743644	-17.14235315038384	-3.53187000095576
C	-3.77782019574215	-19.74183043518805	-3.15002611678988
H	-5.33296837277502	-19.89041658708343	-4.48170424985445
C	-3.07462153833581	-20.92830473830018	-3.87444242247888
C	-2.32875594801771	-21.87905618413108	-3.15394615070676
H	-2.25472634478976	-21.80794591097761	-2.06796790653456
C	-1.67107650843068	-22.92833087820133	-3.80227840539814
H	-1.09940155716103	-23.65076355209490	-3.21274999075341
C	-1.74639048198840	-23.05902621167983	-5.19377500567006
H	-1.23745249238830	-23.88311052426908	-5.70137577196577
C	-2.47966680483845	-22.12084614461482	-5.92414079565182
H	-2.54617729112971	-22.20187955541049	-7.01296639961598
C	-3.13103182360613	-21.06583292454251	-5.27211028225010
H	-3.67903986190961	-20.32988115482697	-5.86044163712883
C	-4.47729720219847	-20.31544224063249	-1.87848792348891
C	-3.83139420858245	-20.40752149047528	-0.63235335121349
H	-2.81849721558525	-20.01902732126926	-0.51198855910176

C	-4.46031773609031	-20.99397980156683	0.47231543658547
H	-3.92926210127704	-21.04981998443832	1.42685859474022
C	-5.75722782234965	-21.50372395878904	0.35898599629159
H	-6.25154522802922	-21.95833287497033	1.22190772575314
C	-6.41333560523707	-21.42404298239507	-0.87349430951840
H	-7.42746292391908	-21.81857518061249	-0.98315512748136
C	-5.77840362053518	-20.83905502453698	-1.97433070621410
H	-6.31556636692386	-20.79764320073750	-2.92446153739885
C	-2.74189460531580	-18.63701666193682	-2.80006972612005
C	-3.01735963986918	-17.67175870685374	-1.81363477131194
H	-3.94046313625313	-17.73359194690021	-1.23488761079689
C	-2.12580548773971	-16.62673824730820	-1.54921291507363
H	-2.36861449114230	-15.89457381713515	-0.77391079990811
C	-0.93299012724349	-16.51756033335576	-2.27141808619800
H	-0.23286509349584	-15.70354714818858	-2.06403820330990
C	-0.64804889175343	-17.46215972620542	-3.26175155016766
H	0.27862651271187	-17.39154706761872	-3.83836637666957
C	-1.54323552240999	-18.50651499874254	-3.52181046803918
H	-1.29651427756776	-19.22878374105509	-4.30127361882365

Table S7: Optimized coordinates of **Sensor-PA complex** at B3LYP/def2-SVP level of theory.

C	-9.77221847706080	-4.99882910722291	-6.03517012864478
C	-10.27009004430092	-4.44084373157000	-7.22830111785694
H	-11.28910918791693	-4.04901630610077	-7.25932583394011
C	-9.48548661738391	-4.37501145229040	-8.38396782086468
H	-9.89975838746273	-3.92837218551381	-9.29232119837115
C	-8.17792110541028	-4.87374076375398	-8.37857400318575
H	-7.56032929305229	-4.81765310901131	-9.27936795891040
C	-7.67464013337782	-5.45325987330905	-7.21057745392978
H	-6.65931694466353	-5.85633890304821	-7.18376612802610
C	-8.46648595687650	-5.51975081729983	-6.05851045286726

H	-8.05021105293550	-5.99085366324746	-5.16625522262580
C	-10.71235343369085	-5.19568294072363	-4.81564715752155
H	-10.25717701857916	-6.55484150679483	-0.31586947810499
C	-9.86141431177559	-5.93140187677387	-1.12280210555414
C	-10.45616325270612	-5.98196335996002	-2.38828955496109
H	-11.31672605705987	-6.63052096519292	-2.55863890864981
C	-9.97164031890548	-5.20097841028705	-3.45129683931911
C	-8.88260618692535	-4.34830057380750	-3.19744481421165
H	-8.48611303257432	-3.71428628424060	-3.99234047024832
C	-8.28792640407365	-4.29257507084510	-1.93232464079026
H	-7.44012609661463	-3.62205361810931	-1.76528341320799
C	-8.77108015720531	-5.08782359521452	-0.88774533039397
H	-8.30450194300361	-5.04689180597602	0.10036742325818
C	-11.74425763280431	-4.04091468604392	-4.69914836414494
C	-13.09422815404195	-4.28894306660333	-4.40425776757375
C	-11.32936780616016	-2.70118686196788	-4.81440914977233
C	-14.00292459815308	-3.23440393287748	-4.24235143242624
C	-13.57827153908597	-1.90927334093670	-4.36436959760662
C	-12.23239503304239	-1.64810096811084	-4.65048074624775
H	-11.88178060063887	-0.61679769327176	-4.74875646823937
H	-10.28614822494017	-2.47258306810386	-5.04163849511379
H	-13.44035280998379	-5.31691801452394	-4.29223853204357
H	-15.04976237051111	-3.45743771392013	-4.01681953279556
H	-14.28655643804058	-1.08588862442132	-4.23742345925696
N	-11.45662479289370	-6.46005911068974	-4.98679066844078
C	-10.69735748068885	-7.69046840979782	-5.16547042284907
H	-12.10722711306937	-6.35120389137506	-5.76496827702217
C	-11.63078904089988	-8.90481333253556	-5.06702288238876
H	-9.93412636105530	-7.76474844146831	-4.37489089402032
H	-10.15429239372729	-7.74170070832920	-6.12991086821566
N	-10.89656907727018	-10.12768204427473	-5.31342957857105
H	-12.41018277902673	-8.80663568088225	-5.84672203796790
H	-12.14430626871135	-8.90408052059864	-4.08449626713703
C	-10.91097799214940	-11.03888239912308	-4.41990551992593
C	-10.15410189704313	-12.31159483369938	-4.56451578041910
C	-10.22388719503098	-13.40387133980180	-3.64111714829090
C	-9.40762174123902	-14.50596641376958	-3.92968926366307
H	-9.39997510679510	-15.37148313695922	-3.27092799282053

C	-8.55520358716083	-14.59686534080771	-5.04321439919298
C	-8.51897499308488	-13.51249768943910	-5.94591293488661
C	-9.31364506158565	-12.39706304930981	-5.68851492240671
H	-9.28675055211309	-11.54618269268189	-6.37306930954343
H	-11.48831529466051	-10.88803786895259	-3.49684908953123
O	-7.70503051898605	-13.58603301055274	-7.02703692118495
H	-7.78598496887710	-12.77701915399536	-7.55579537175572
C	-11.13149260073366	-13.43686751531137	-2.38075789009305
C	-12.62228712459896	-13.27602401530458	-2.77256158663637
H	-12.85031201320837	-12.31619083097739	-3.25515337193826
H	-13.25550628574746	-13.35026369905079	-1.87304744327762
H	-12.92544354836968	-14.07836752911718	-3.46484022046014
C	-11.02748643639215	-14.78756141344170	-1.63714264854580
H	-11.69652717862081	-14.76659704282544	-0.76256885457376
H	-10.00928799780750	-14.98364284884201	-1.26729004633811
H	-11.33510601526541	-15.63471216457313	-2.26985984932317
C	-10.69923017838820	-12.34946232914268	-1.36431887996547
H	-10.77874837741375	-11.32595279756843	-1.75466628402946
H	-9.65293138771664	-12.50769420882257	-1.05625945732978
H	-11.32922508329755	-12.40810010770003	-0.46128533539779
C	-7.70887215799498	-15.78114249616051	-5.26691741041943
N	-7.66185720474083	-16.76882997692668	-4.45849121781775
H	-7.09871186566995	-15.76685700409044	-6.18866117827578
C	-6.77109692928647	-17.86740487936587	-4.76770120151919
C	-5.72627436278979	-18.04023702214424	-3.65695548832661
H	-6.25277054959425	-17.73842469311505	-5.73935340316376
H	-7.37298019470575	-18.79415417931986	-4.83349848735501
N	-4.85995146567698	-19.16893951694954	-3.96297216832696
H	-6.25346838000748	-18.12618952014860	-2.68420265996738
H	-5.12373957227772	-17.12102289041904	-3.60684733727354
C	-3.73167394423525	-19.48874533690716	-3.06635622239126
H	-5.42477482602775	-20.00416819986039	-4.11259440675218
C	-3.00014958763557	-20.72674961295640	-3.66744083902038
C	-2.03010177071648	-21.42463344368131	-2.92459135487772
H	-1.79428410882332	-21.11548271247867	-1.90543444657672
C	-1.34988871865745	-22.51912781179901	-3.46537455890106
H	-0.60077223767849	-23.03852737292481	-2.86101073509073
C	-1.62627044010817	-22.95164019457163	-4.76772455681328

H	-1.09903474855000	-23.81174089408582	-5.18959761689577
C	-2.58328723083886	-22.26707122662156	-5.52006984192366
H	-2.80956439441613	-22.58441921824950	-6.54214810802334
C	-3.25725192644214	-21.16485386153591	-4.97774662170223
H	-3.98167783724317	-20.62905278015866	-5.59088262686857
C	-4.19641971036512	-19.87535757685380	-1.62819928726563
C	-3.38282674961936	-19.70145815350585	-0.49359260265459
H	-2.40522489633407	-19.22552531233787	-0.58889688736630
C	-3.79818449067435	-20.12810901968136	0.77323827222851
H	-3.14049471384041	-19.97669141800176	1.63390465968004
C	-5.04337690271875	-20.74185319388217	0.93949709917627
H	-5.37084865647698	-21.07244792422353	1.92899932674479
C	-5.86390523483594	-20.92669433776442	-0.17765163194827
H	-6.84076030525382	-21.40642739210561	-0.06927690644973
C	-5.44233876489055	-20.49948842729723	-1.44142539957723
H	-6.10361758953891	-20.66492115642872	-2.29472773369534
C	-2.78547959809171	-18.25541714164537	-3.07402835288120
C	-2.98650961635563	-17.17048297948629	-2.20133072755946
H	-3.77278261280386	-17.21570487808520	-1.44598224830821
C	-2.19688439336269	-16.01769484414246	-2.27973692250510
H	-2.38037818437118	-15.19203402198457	-1.58636829849470
C	-1.18578670106792	-15.91840521459845	-3.24021645351460
H	-0.57689267201399	-15.01735432074613	-3.30897883741148
C	-0.97821064297632	-16.98460477850044	-4.12099226682503
H	-0.19411354695619	-16.92437740041531	-4.88116210397251
C	-1.76912442749298	-18.13586580838864	-4.03760889409612
H	-1.58845452352919	-18.95046503473306	-4.74084374339444
C	-3.28816107955721	-9.83453989468129	-5.44110800261119
C	-2.04122006332106	-10.49466091218469	-5.20822914888278
C	-3.21462513592540	-8.42100260094450	-5.60339583419774
C	-0.83304133242088	-9.80758837281125	-5.12980021202908
C	-0.83453546648854	-8.42979081768148	-5.29700524259060
C	-2.01465468460615	-7.72978088841669	-5.54765905786841
H	0.09254611908231	-10.35068824188481	-4.94617838776750
H	-1.99833963133779	-6.65079027127421	-5.69536386647744
O	-4.43034529804687	-10.46511352455714	-5.53693474228587
H	-4.24440834163855	-11.43776502745658	-5.43445873574682
N	-4.42927387006002	-7.62897798930295	-5.85295353565335

O	-5.47908884610877	-8.01240243162780	-5.36934390724893
O	-4.29750799172820	-6.61434076905120	-6.52165872528657
N	-1.99921665736714	-11.94700349514478	-5.05804725656533
O	-3.06617725228376	-12.56898289101287	-5.15304106614020
O	-0.93212395119606	-12.48279032315451	-4.85552177063355
N	0.43493836886646	-7.69404446973979	-5.22007350704998
O	1.45296371610418	-8.33936808477945	-5.02723895995153
O	0.39468985030771	-6.48138035221774	-5.35542704034875

Table S8: Optimized coordinates of PA at B3LYP/def2-SVP level of theory.

C	-2.75231994998661	-9.71632531924071	-4.97943598998225
C	-1.49401331180902	-10.40278744104445	-4.95668834418656
C	-2.68936601858878	-8.32508526152635	-5.29783093979798
C	-0.28602750575450	-9.77062147686280	-5.22948054618705
C	-0.29630842071501	-8.41694676657759	-5.53627472338876
C	-1.48468691926176	-7.69095943262760	-5.56975500078543
H	0.64469434685899	-10.33483957310457	-5.20105718442232
H	-1.48085477556734	-6.62929794359042	-5.80925809800711
O	-3.88412281773580	-10.31714300507844	-4.72183006749695
H	-3.67273518620474	-11.27456442524330	-4.53498845011673
N	-3.90229014978693	-7.48537506715531	-5.35818435248643
O	-4.98751879171132	-8.01099730504943	-5.18727418103667
O	-3.74369091171293	-6.29413457519572	-5.58319398289758
N	-1.44162556355253	-11.82663762378032	-4.63460124197885
O	-2.51289200956743	-12.41287003495135	-4.41771794341292
O	-0.36552049284124	-12.38214830168919	-4.59203017547837
N	0.97181238250936	-7.73608989616402	-5.83015397480665
O	1.99413486769120	-8.40265252165320	-5.79399049463764
O	0.92762122773641	-6.54485402946514	-6.09389430889370

References:

1. S. S. Pasha, H. R. Yadav, A. R. Choudhury and I. R. Laskar, *Journal of materials chemistry C*, 2017, **5**, 9651-9658.
2. H. Li, B. Fu, W. Yang, L. Ding, Y. Yang, J. Dong, F. Wang and Q. Pan, *Spectrochimica Acta Part A: Molecular and Biomolecular Spectroscopy*, 2020, **226**, 117575.
3. S. Halder, A. Bhattacharjee, A. Roy, S. Chatterjee and P. Roy, *RSC Adv.*, 2016, **6**.
4. A. S. Tanwar, S. Patidar, S. Ahirwar, S. Dehingia and P. K. Iyer, *Analyst*, 2019, **144**, 669-676.
5. J. R. Lakowicz, *Principles of fluorescence spectroscopy*, Springer, 2006.
6. S. Panigrahi and A. Mishra, *Journal of Photochemistry and Photobiology C Photochemistry Reviews*, 2019, **41**, 100318.
